# Elucidating the linagliptin and fibroblast activation protein binding mechanism through molecular dynamics and binding free energy analysis

**DOI:** 10.1016/j.isci.2024.111368

**Published:** 2024-11-13

**Authors:** Mingsong Shi, Fang Wang, Zhou Lu, Yuan Yin, Xueting Zheng, Decai Wang, Xianfu Cai, Meng Jing, Jianjun Wang, Junxian Chen, Xile Jiang, Wenliang Yu, Xiaoan Li

**Affiliations:** 1 NHC Key Laboratory of Nuclear Technology Medical Transformation, Mianyang Central Hospital, School of Medicine, University of Electronic Science and Technology of China, Mianyang, Sichuan 621099, China; 2 Department of Clinical Nutrition, Innovation Center of Nursing Research, Nursing Key Laboratory of Sichuan Province, West China Hospital, Sichuan University, Chengdu, Sichuan 610041, China; 3 Department of Hepatobiliary Surgery, Mianyang Central Hospital, School of Medicine, University of Electronic Science and Technology of China, Mianyang, Sichuan 621099, China; 4 Department of Pathology, Mianyang Central Hospital, School of Medicine, University of Electronic Science and Technology of China, Mianyang, Sichuan 621099, China; 5 Key Laboratory of General Chemistry of the National Ethnic Affairs Commission, School of Chemistry and Environment, Southwest Minzu University, Chengdu 610041, Sichuan, China; 6 Department of Obstetrics and Gynecology, Mianyang Central Hospital, School of Medicine, University of Electronic Science and Technology of China, Mianyang, Sichuan 621099, China; 7 Department of Gastroenterology, Mianyang Central Hospital, School of Medicine, University of Electronic Science and Technology of China, Mianyang, Sichuan 621099, China

**Keywords:** Biological sciences, Chemistry, Physics

## Abstract

Fibroblast activation protein (FAP) is highly expressed in solid tumors and may be a potential diagnostic and therapeutic target in solid cancers. Linagliptin inhibits FAP; however, the interaction mechanism between linagliptin and FAP remains unclear. In this study, the binding free energy for linagliptin with human FAP was estimated at −13.66 kcal/mol, and the dissociation constant was 243 nM based on surface plasmon resonance analyses. E203, E204, and Y656 formed hydrogen bonds with ammonium. Y625 formed an unstable hydrogen bond with the carbonyl group. W623 and Y541 interacted with the quinazoline and pyrimidine-2,4-dione rings, respectively, via π–π interactions. The butyne group formed hydrophobic interactions with residues V650, Y653, Y656, and Y660. ZINC000299754517 and ZINC000299754576 were identified as potential FAP inhibitors. The R1 and R4 regions of linagliptin could be optimized to increase its FAP binding affinity. These findings can guide linagliptin structural optimization to improve its FAP binding affinity.

## Highlights

• Linagliptin has a binding free energy of −13.66 kcal/mol to the human FAP
• Linagliptin has a dissociation constant of 243 nM
• ZINC000299754517 and ZINC000299754576 are potential FAP inhibitors
• Linagliptin’s R1 and R4 regions could be optimized for increased FAP binding

## Graphical abstract

**Figure:**
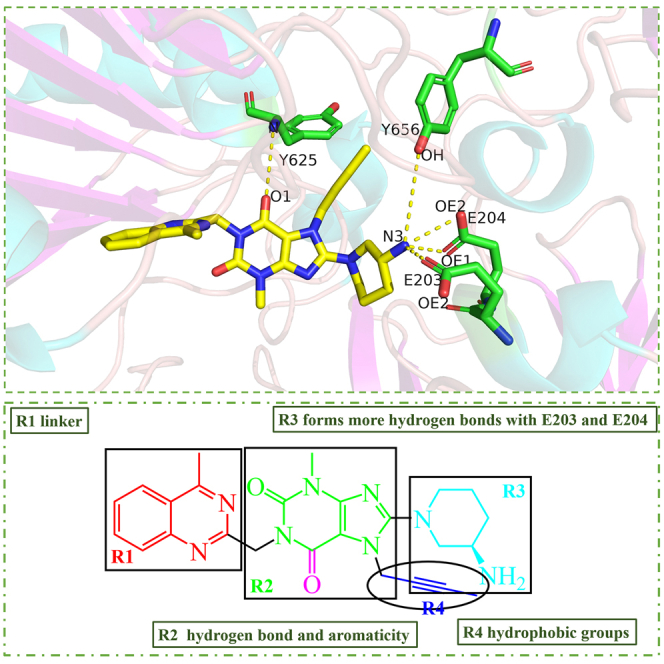

## Introduction

Fibroblast activation protein (FAP), formerly known as the cell surface F19 antigen, is typically expressed at low levels or absent in normal tissues.^1^^,^^2^ FAP is expressed in most solid cancers, including distal cholangiocarcinoma,^3^ hepatocellular carcinomas,^4^ esophageal squamous cell carcinoma,^5^ pancreatic adenocarcinomas,^6^ osteosarcomas,^7^ esophageal adenocarcinomas,^8^ hepatocellular carcinomas,^9^ urothelial carcinomas,^10^ and epithelial ovarian carcinoma,^11^ as well as other diseases.^12^^,^^13^^,^^14^^,^^15^ FAP has pivotal enzymatic and non-enzymatic functions in solid tumors, such as the migration, invasion, adhesion, and proliferation of tumor cells,^12^^,^^16^^,^^17^^,^^18^ extracellular matrix remodeling,^19^^,^^20^ cancer-associated fibroblast morphology,^18^^,^^21^ angiogenesis, and immunosuppression.^2^ These findings indicate that FAP may serve as a diagnostic marker and a therapeutic target for solid cancers.

**Figure 1:**
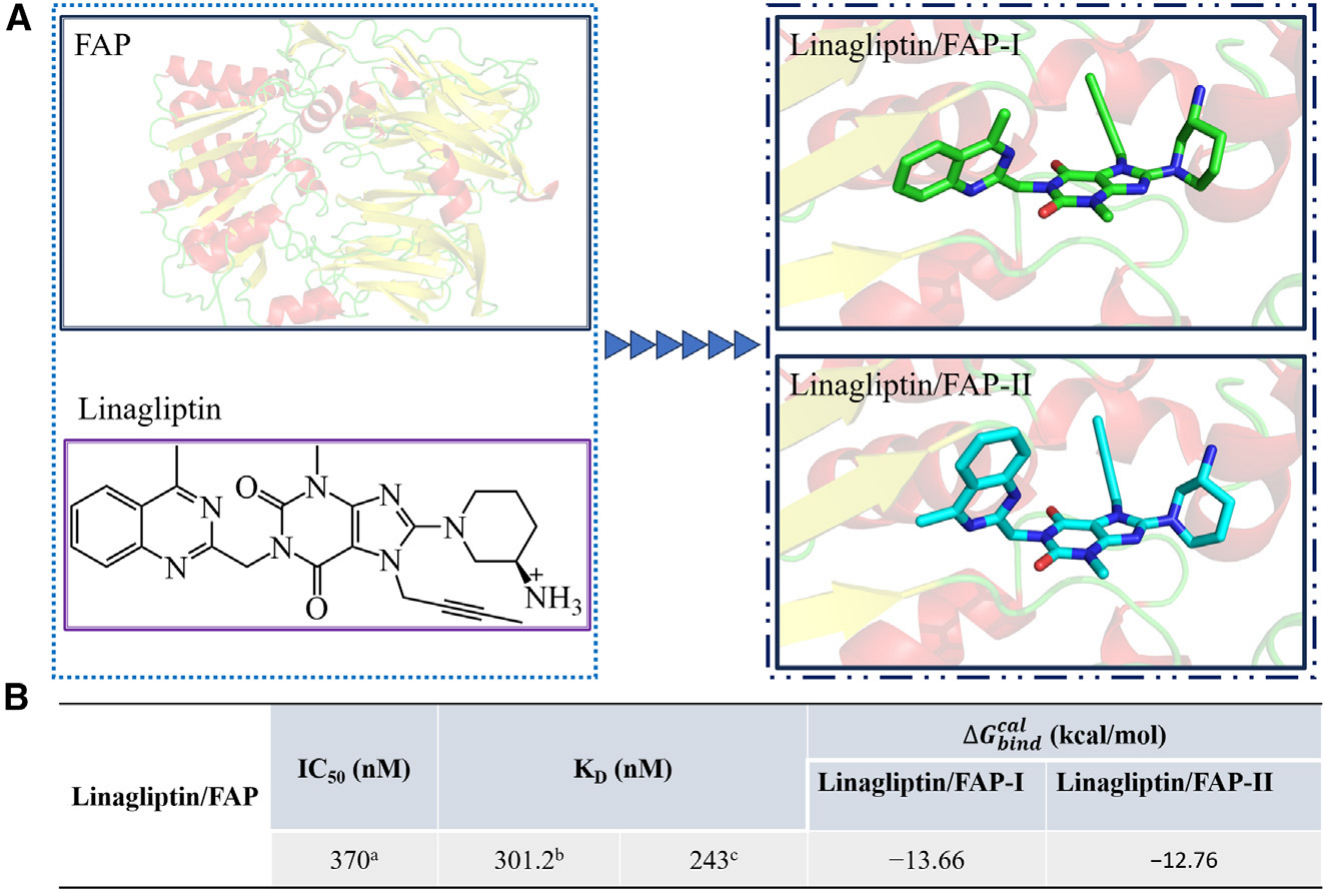
Linagliptin and FAP structures and their binding affinity (A) The human FAP structure in cartoon form; the linagliptin structure in stick form; C: green, O: red, and N: blue in the crystal structure of linagliptin/FAP and C: cyan, O: red, and N: blue in the crystal structure of linagliptin/DPP-4. (B) Linagliptin binding affinity with FAP; (a) data extracted from ref. 44; (b) data extracted from 48; (c) determined from surface plasmon resonance experiments of the current study. FAP, fibroblast activation protein; DPP-4, dipeptidyl peptidase-4.

FAP belongs to the type II transmembrane protein family comprising 760 amino acids and a short cytoplasmic tail (residue number: 1–4). The transmembrane protein domain contains 20 amino acids (residue number: 5–25) and an approximately 740-amino acid extracellular region (residue number: 26–760), comprising β-propeller and αβ-hydrolase domains at the N- and C-terminals, respectively (Figure 1). The transmembrane protein domain contains serine, aspartate, and histidine residues that catalyze hydrolase activity and regulate substrate availability.

Over the last decade, several inhibitors and diagnostic agents targeting the FAP have been developed for tumor diagnosis and treatment.^15^^,^^22^^,^^23^^,^^24^^,^^25^ Recently, FAP-targeted diagnostic and therapeutic strategies have gained considerable research interest, particularly in nuclear medicine.^26^^,^^27^^,^^28^^,^^29^ FAP-targeted radiopharmaceuticals derived from small molecules or peptides are currently the most promising diagnostic markers in nuclear medicine, such as in positron emission tomography and single photon emission computed tomography and cancer treatment. FAP inhibitors (FAPIs), such as FAPI-46, FAPI-04, and FAPI-02 (Figure S1), have shown promise as diagnostic and therapeutic markers because of their strong affinity, high specificity, and favorable pharmacokinetic profiles.^30^^,^^31^^,^^32^^,^^33^^,^^34^^,^^35^ Several FAPIs are currently undergoing clinical trials.^36^^,^^37^^,^^38^^,^^39^ Owing to their short retention times at tumor sites,^40^^,^^41^^,^^42^^,^^43^ these FAPIs cannot be widely used. Hence, the tumor retention times of radiotracers, such as FAP-2286, must be improved for use in radionuclide therapy to improve therapeutic outcomes in tumor-bearing mice. Therefore, it is necessary to develop novel FAPIs.

The long-acting dipeptidylpeptidase 4 (DPP-4) inhibitor linagliptin (known as BI 1356, CAS: 668270-12-0) is a potent and competitive drug that exerts long-lasting effects on glucose tolerance by regulating glucagon-like peptide-1 and insulin.^44^^,^^45^^,^^46^ Additionally, linagliptin is a potential FAP inhibitor, with a half-maximal inhibitory concentration (IC_50_) of 89.9 ± 15.4 nM (IC_50_ = 1.4 ± 1.1 nM for DPP-4).^47^^,^^48^ Furthermore, its dissociation constants (K_D_) are 301.2 ± 103.3 and 0.0066 ± 0.00034 nM for FAP and DPP-4, respectively.^48^ Considering the high inhibitory activity of linagliptin against FAP, it can be used as a lead compound to design new or refine existing FAPIs. Although linagliptin may not have potential in cancer therapy,^48^^,^^49^ FAP is widely applied in nuclear medicine.

In a previous study, the crystal structure of the linagliptin/FAP binding complex was determined,^48^ and the biophysical and structural characteristics of linagliptin binding to DPP-4 and FAP were compared. The binding model for linagliptin/FAP was relatively identical to that of linagliptin/DPP-4, excluding the distal quinazoline group of linagliptin, the binding mechanism of which remains unclear. Moreover, the single-second shell residue (A657) was responsible for the different affinity and kinetics properties of linagliptin binding with FAP and DPP-4, affecting the dissociation and association processes. Meanwhile, the impact of A657 on the equilibrium state remains unknown, and the details of the interaction between linagliptin and FAP have yet to be elucidated. Nevertheless, the conformations of the receptor-ligand complexes created by linagliptin and FAP or DPP-4 differ; therefore, its conformation in a solvent environment may affect its binding affinity for FAP.^48^^,^^50^ This has hindered the development and refinement of selective FAPIs, especially in nuclear medicine. Hence, to realize human FAP-targeted radiopharmaceutical therapy, a novel target molecule capable of binding FAP is required.

In this study, the binding free energy of the linagliptin/FAP system was analyzed using molecular dynamics (MD) simulations and binding free energy calculations. A binding model of linagliptin/FAP was obtained from the crystal structure of the linagliptin/FAP-I system, and the conformational structure of linagliptin binding with DPP-4 was used to generate a linagliptin/FAP-II system. For full relaxation rates, MD simulations were conducted over an extended period. Binding free energies were calculated using the molecular mechanics generalized Born surface area (MM/GBSA) method. Moreover, hot residues involved in linagliptin binding to FAP were analyzed using per-residue energy decomposition. These simulation results may provide useful information for the future design of linagliptin-based FAP-targeted inhibitors.

## Results and discussion

### System stability

For the crystal structures of linagliptin binding human FAP, the solvent environment was not considered. However, residue rearrangement around the active site, such as that of the second shell residue A657, was considered.^48^ Thus, the binding mechanism of linagliptin with FAP was studied using MD simulations. Over 500 ns MD simulations of the linagliptin/FAP-I complex were performed thrice.

**Figure 2:**
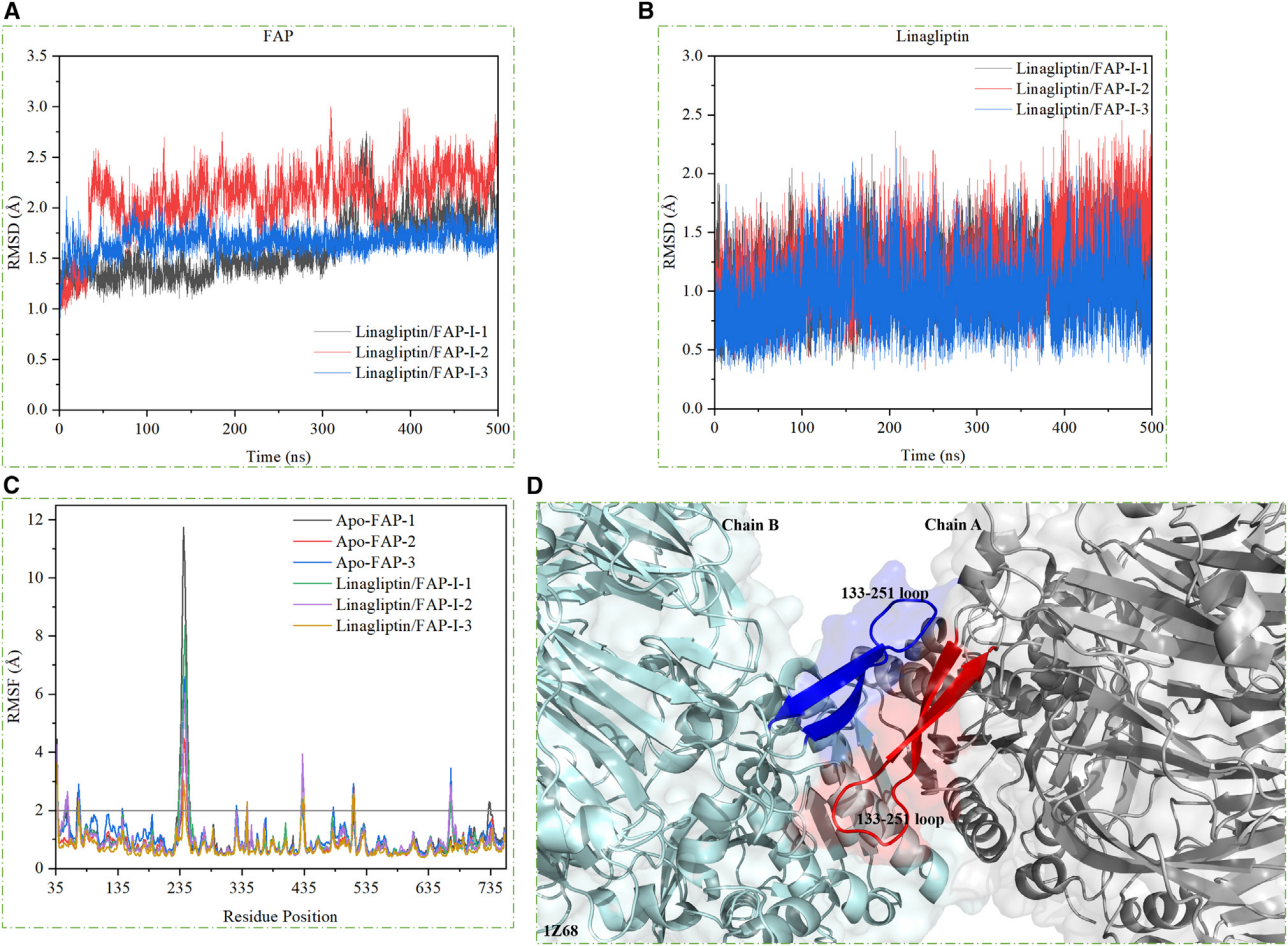
System stability of the linagliptin/FAP-I complexes (A) Root-mean-square deviation (RMSD) values of the heavy backbone atoms for human FAP throughout the 500 ns molecular dynamic (MD) simulations of the linagliptin/FAP-I systems. (B) RMSD values of the non-hydrogen bond atoms of linagliptin during the 500 ns MD simulation of the linagliptin/FAP-I systems. (C) Root-mean-square fluctuation (RMSF) variations for the linagliptin/FAP-I systems during the 500 ns MD simulation. (D) Interface of the FAP homodimer. FAP, fibroblast activation protein.

The root-mean-square deviations (RMSDs) of FAP were calculated using heavy atoms in the residue backbones (C, CA, O, and N, Figure S2). RMSD values of linagliptin were calculated using its non-hydrogen atoms and were 1.14 ± 0.22, 1.22 ± 0.29, and 0.97 ± 0.27 Å for linagliptin/FAP-I-1, linagliptin/FAP-I-2, and linagliptin/FAP-I-3, respectively (Table S1). This showed that linagliptin remained in the binding site of FAP, with a similar conformation among the study simulations. Meanwhile, the RMSD values for FAP (linagliptin/FAP-I-1: 1.61 ± 0.30, linagliptin/FAP-I-2: 2.11 ± 0.31, and linagliptin/FAP-I-3: 1.67 ± 0.11 Å) were greater than those of linagliptin. However, FAP and linagliptin conformations equilibrated in the last 400 ns of the experiment (Figure 2), apart from the FAP conformation in the linagliptin/FAP-I-1 system (Figure S3). In addition, the surface area (Figure S4) and gyration radius (Figure S5) measurements showed stable FAP and linagliptin conformations after 100 ns. Therefore, these simulations could be used to analyze the interactions between linagliptin and FAP.

A root-mean-square fluctuation (RMSF) analysis was conducted to quantify the flexibility of specific residues during MD simulations. Based on the 500 ns MD trajectory for the linagliptin/FAP complex, RMSF values were calculated for all residues (Figure 2). The 233–251 amino acids in the β-propeller domain 4 (BD4) of the human FAP showed higher flexibility than those of other FAP regions, with higher RMSF values. Moreover, these residues play an important role in the dimer formation of human FAP (Figure S6).^51^ Accordingly, the loop region in the BD4 of FAP was unstable during simulations (Figure S7). However, the FAP monomer was used as the model for investigating the interaction between linagliptin and FAP, potentially resulting in loop flexibility (residues: 233–251) during the simulations. For FAP to function catalytically, it must be dimerized.^51^^,^^52^ Therefore, the dimeric structure of FAP should be studied in future studies.

In addition, apo-FAP complexes without linagliptin bound to the catalytic site were simulated for 500 ns to determine the FAP conformation after linagliptin binding. The apo-FAP systems were not stable during the simulation, potentially due to the unbound FAP monomer and linagliptin (Figure S8). The fluctuations in the FAP structure were not affected by linagliptin binding (Figure S9). The other region of the FAP structure remained stable during the simulation. Overall, these observations suggest that human FAP conformations are stable, excluding the loop region in the BD4, which forms a dimer with other FAP molecules.

The snapshots extracted at 100, 200, 300, 400, and 500 ns were aligned to depict the linagliptin/FAP-I (Figures S10–S12) and apo-FAP systems (Figures S13–S15). For the two systems, conformations of the initial and last 500-ns MD trajectories were constructed to identify differences between the initial and refined binding models, which were obtained from the MD simulations (Figure S16). In these simulations, FAP adopted a similar conformation to its initial conformation, and there were no differences in binding modes between the initial structure of linagliptin and its initial conformation, except for the quinazoline group. Thus, linagliptin maintained its binding ability for the FAP catalytic site.

### Hydrogen bond analysis

**Figure 3:**
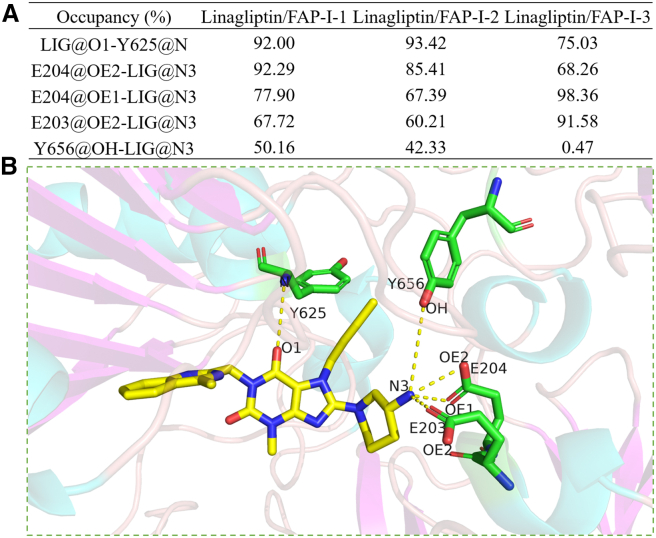
Analysis of the hydrogen bonds between human FAP and linagliptin (A) Occupancy analysis for hydrogen bonds. (B) The scheme for hydrogen bonds. Occupancy determined by the percentage of the simulation period (500 ns, 50000 frames) during which specific hydrogen bonds formed. Hydrogen bonds confirmed by a distance of <3.5 Å between the acceptor and donor atoms, with an internal angle >120° between the H-acceptor and H-donor. FAP, fibroblast activation protein.

In the simulations, hydrogen bonds were identified between FAP and linagliptin, revealing more than four hydrogen bonds between linagliptin and FAP (Figure S17). Linagliptin/FAP-I-1, linagliptin/FAP-I-2, and linagliptin/FAP-I-3 exhibited average hydrogen bond numbers of 4.40 ± 0.86, 4.11 ± 0.81, and 3.61 ± 0.80, respectively, in the 500 ns simulation (Figure 3). Therefore, approximately four hydrogen bonds were formed between linagliptin and FAP.

Furthermore, hydrogen bond occupancy was determined for the linagliptin/FAP-I complex in the 500 ns simulation. A hydrogen bond was formed between the O1 atom of the carbonyl group in linagliptin as an acceptor atom and the nitrogen atom in the amino group of the Y625 residue of FAP as a donor atom (>92.00% occupancy, excluding the linagliptin/FAP-I-3 system, with 75.03% occupancy). A hydrogen bond (LIG@O1-Y625@N) was observed between the carbonyl group at C6 of linagliptin and Y631 backbone of DPP4 in linagliptin/DPP-4.^48^^,^^50^ Further, the distances between the donor and acceptor atoms and the angles among the hydrogen, donor, and acceptor atoms in the MD simulations were calculated (Table S2). The distance between the N atom of Y625 and O1 of linagliptin was 3.20 ± 0.20, 3.17 ± 0.19, and 3.33 ± 0.25 Å for linagliptin/FAP-I-1, linagliptin/FAP-I-2, and linagliptin/FAP-I-3, respectively (Figure S18), indicating a stable hydrogen bond throughout the simulation period. Moreover, the angles between the three hydrogen bond atoms were 148.60 ± 11.44° (linagliptin/FAP-I-1), 148.11 ± 11.30° (linagliptin/FAP-I-2), and 142.93 ± 11.58° (linagliptin/FAP-I-3). These data indicate that the LIG@O1-Y625@N hydrogen bond was stable and strong during the simulations.

E203 and E204 are residues present in the recognition site of the amino terminus of peptide substrates of the human FAP. These residues form hydrogen bonds with substrates and linagliptin. The occupancy of the E203@OE2-LIG@N3 interaction was 91.58% in the linagliptin/FAP-I-3 complex but reduced to 67.72% and 60.21% in the linagliptin/FAP-I-1 and linagliptin/FAP-I-2 systems, respectively. These hydrogen bonds were confirmed by the distances between the OE2 atom of E203 and the N3 atom of linagliptin, which were 3.34 ± 0.91 Å (linagliptin/FAP-I-1), 3.44 ± 0.93 Å (linagliptin/FAP-I-2), and 2.79 ± 0.16 Å (linagliptin/FAP-I-3; Figure S19). The angle between linagliptin and E203 was approximately 91.73 ± 46.57° for the three simulations (Table S3). This indicates that the hydrogen bond formed between E203 and linagliptin was unstable due to the simulation.

E204 formed two strong hydrogen bonds with the N3 atom of linagliptin. The two oxygen atoms (OE1 and OE2) in the carboxyl group of E204 formed hydrogen bonds in the linagliptin/FAP-I-1 (77.90% and 92.29%), linagliptin/FAP-I-2 (67.39% and 85.41%), and linagliptin/FAP-I-3 (98.36% and 68.26%) structures. Occupancy analysis showed that OE1 and OE2 formed stable hydrogen bonds with linagliptin. The distances of the E204@OE1-LIG@N3 bonds were 3.18 ± 0.49 Å (linagliptin/FAP-I-1), 3.33 ± 0.70 Å (linagliptin/FAP-I-2), and 2.84 ± 0.20 Å (linagliptin/FAP-I-3; Figure S20). However, the distance was <3.00 Å for E204@OE2-LIG@N3 (Figure S21), suggesting that E204@OE2-LIG@N3 is stronger than E204@OE1-LIG@N3. This can be determined using the angle between the two hydrogen bonds. Thus, a strong and stable hydrogen bond was formed between E204 and linagliptin.

Additionally, an unstable hydrogen bond was formed between Y656 and the amino group of linagliptin in linagliptin/FAP-I-1 and linagliptin/FAP-I-2, with an occupancy of 50.16% and 42.33%, respectively. However, the Y656@OH-LIG@N3 bond was not detected in the linagliptin/FAP-I-3 system, implying that the hydrogen bond was unstable during the simulation. Furthermore, the distance of the bond was >5.00 Å, and the angle was approximately 100° in the linagliptin/FAP-I-3 system (Figure S22). Thus, the hydrogen bonds formed between Y656 and linagliptin were unstable.

Two glutamates (Glu motifs: E203 and E204 for human FAP and E205 and E206 for human DPP-4) were used to detect the charged N-terminal of the substrate peptides for the serine protease family. Previously, the Glu motif was used to design a selective FAPI.^53^ Additionally, the Glu motif of DPP-4 plays an important role in its aminopeptidase activity. The 3-aminopiperidine group is important for DPP-4-targeting activity; the IC_50_ value decreased from 2,800 nM with the piperazine group to 82 nM with the 3-aminopiperidine group (Figure S23).^50^ The positive ammonium of the 3-aminopiperidine group formed three strong hydrogen bonds with FAP and DPP-4.^50^ Based on the high sequence homology between DPP-4 and FAP, the 3-aminopiperidine group plays a key role in the binding of DPP-4 with FAP. Meanwhile, the R conformation of the primary amino group was better than the S conformation in the binding of FAP with DPP-4 (Figure S24), indicating that the R conformation may be better than the S conformation for binding with human FAP. Thus, the 3-aminopiperidine group of linagliptin recognizes the Glu motif site and binds to the S2̛ pocket to inhibit the catalytic activity of the human FAP.

### Binding free energies

An MD simulation was used to visualize the interaction between linagliptin and FAP. However, the MD simulations could not determine the binding affinity of linagliptin for FAP. Thus, the binding free energies of linagliptin and FAP were calculated using MM/GBSA (Tables 1 and S4–S7). Negative entropy ($\Delta TS_{total}$) or enthalpy (${\Delta E}_{\text{gas}}+{\Delta E}_{\text{sol}}$) values (less than −51.37 and −37.65 kcal/mol, respectively) were observed for the binding complexes, indicating the involvement of enthalpy-driven processes in forming the complexes. For linagliptin/FAP-I, the binding free energies ($\Delta G_{bind}^{cal}$) were approximately −13.72, −13.55, and −13.69 kcal/mol for linagliptin/FAP-I-1, linagliptin/FAP-I-2, and linagliptin/FAP-I-3, respectively. Therefore, linagliptin/FAP-I can be considered a reliable binding model of linagliptin binding to FAP. Based on the calculations, the linagliptin/FAP-I-1 system exhibited the lowest binding free energy. Additionally, the binding affinity of linagliptin/FAP-I was strong, consistent with experimental results (IC_50_ = 0.37 μM and K_D_ = 0.30 μM).^44^^,^^48^ Meanwhile, the K_D_ was determined from the surface plasmon resonance experiments to be 0.24 μM (Figure S25). The IC_50_ value was 137.3 nM (Figure S26), similar to those reported in previous studies (K_D_ = 301.2 nM,^48^ IC_50_ = 89.9 nM^48^ and 0.37 μM^44^).

**Table 1:** van der Waals interactions ($E_{vdW}$), decomposition and electrostatic interactions ($E_{ele}$), solvation free energies ($E_{polar}$), nonpolar solvation energies ($E_{nonpolar}$), entropy ($TS_{total}$), and binding free energies ($G_{bind}^{cal}$) of the linagliptin/FAP systems

Energy (kcal/mol)	Linagliptin/FAP-I-1	Linagliptin/FAP-I-2	Linagliptin/FAP-I-3	Linagliptin/FAP-II-1	Linagliptin/FAP-II-2	Linagliptin/FAP-II-3
${\Delta E}_{vdW}$	−52.02	−52.56	−53.97	−50.88	−53.41	−52.25
$\Delta E_{ele}$	−208.66	−204.50	−234.72	−216.63	−208.56	−226.22
$\Delta E_{polar}$	214.83	210.87	242.74	221.47	216.69	232.61
${\Delta E}_{nonpolar}$	−5.53	−5.32	−5.85	−5.29	−5.44	−5.36
${\Delta E}_{gas}$	−260.67	−257.05	−288.68	−267.50	−261.96	−278.46
${\Delta E}_{solv}$	209.30	205.55	236.89	216.18	211.24	227.24
$\Delta (E_{gas}+E_{sol)}$	−51.37	−51.51	−51.79	−51.33	−50.72	−51.22
$\Delta TS_{total}$	−37.65	−37.95	−38.10	−39.33	−37.47	−38.20
$\Delta G_{bind}^{cal}$	−13.72	−13.55	−13.69	−12.00	−13.24	−13.02

FAP, fibroblast activating protein.

Binding energy can be categorized into polar ($E_{\text{ele}}$ + $E_{\text{polar}}$) and nonpolar ($E_{\text{vdW}}$ + $E_{\text{nonpolar}}$) components. The polar terms for the linagliptin/FAP-I systems were >6.17 kcal/mol. Based on the positive polar terms, linagliptin and FAP had antagonistic polar interactions (Figure S27). In contrast, the nonpolar values for the linagliptin/FAP-I systems were <–57.55 kcal/mol. The negative value of the nonpolar terms indicated that they were the main contributors to the binding of linagliptin to FAP. Thus, the polar term was not favorable for linagliptin binding to human FAP. The van der Waals ($E_{\text{vdW}}$) interactions (linagliptin/FAP-I-1: −52.02 kcal/mol, linagliptin/FAP-I-2: −52.56 kcal/mol, and linagliptin/FAP-I-1: −53.97 kcal/mol) were the main nonpolar contributors. Hence, van der Waals interactions were the primary factors responsible for linagliptin binding to human FAP. Additionally, this suggests that FAP inhibitors should target van der Waals interactions to improve their potency.

### Free energy decomposition

The binding model was elucidated using MD simulations. However, there is a lack of information regarding the key residues involved in the binding of linagliptin to human FAP. An analysis of inhibitor-protein interactions can be performed using the per-residue decomposition of free energy. Thus, the MM/GBSA decomposition procedure was performed to estimate the interaction energies between human FAP residues and linagliptin (Tables S8–S10).

**Figure 4:**
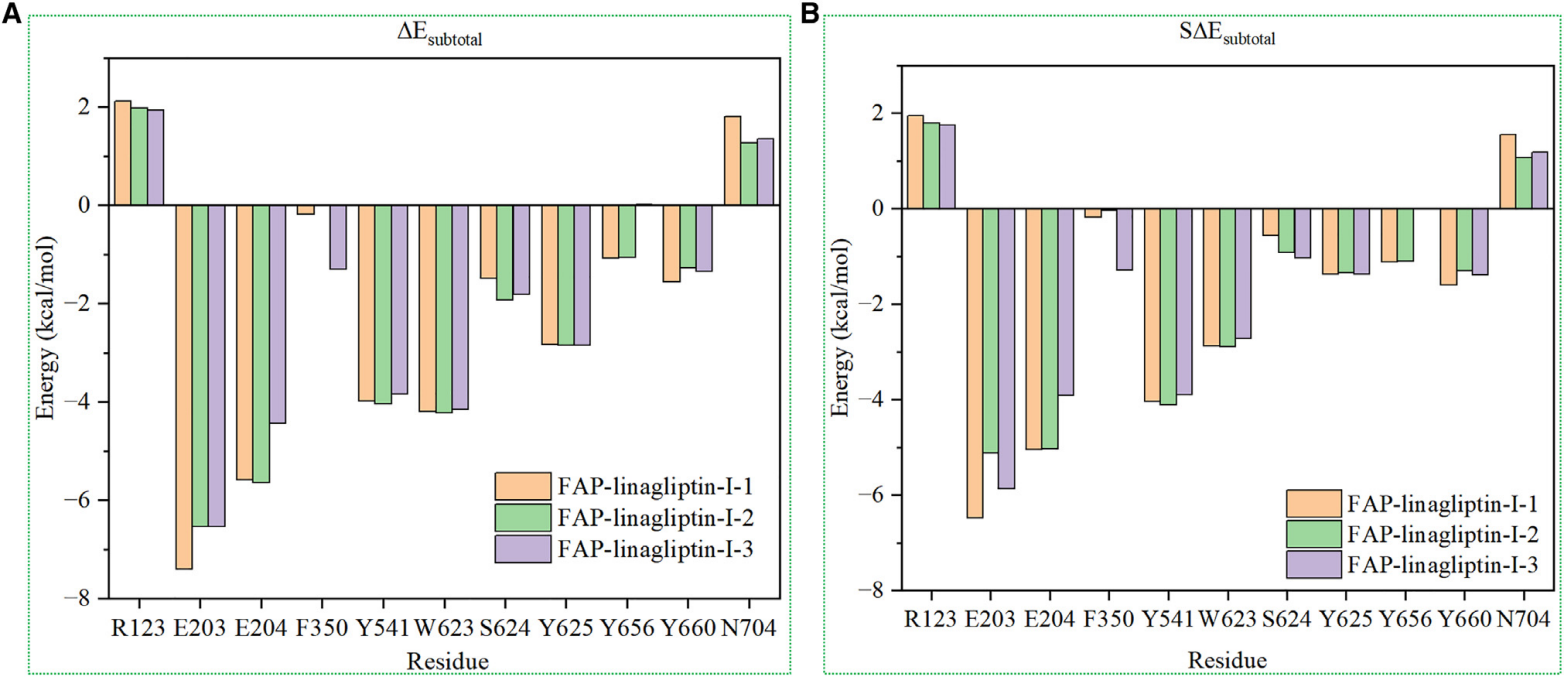
Decomposition energies of residues in the binding of linagliptin to human FAP in different linagliptin/FAP-I systems (A) Partial residue contributes energies ($\Delta E_{subtotal}$) to the binding of linagliptin to FAP. (B) Partial side chain residue contributes energies ($S\Delta E_{subtotal}$) to the binding of linagliptin to FAP. FAP, fibroblast activation protein.

Two residues (E203 and E204) in the Glu motif contributed the most to the binding energy between linagliptin and FAP. Specifically, the energies of E203 were −7.40, −6.54, and −6.54 kcal/mol for linagliptin/FAP-I-1, linagliptin/FAP-I-2, and linagliptin/FAP-I-3, respectively (Figure 4). Meanwhile, E204 contributed energies of −5.58, −5.64, and −4.44 kcal/mol to the linagliptin/FAP-I-1, linagliptin/FAP-I-2, and linagliptin/FAP-I-3 complexes, respectively. The carboxylate group in the side chain of glutamic acid (E203 and E204) formed hydrogen bonds with the ammonium group in linagliptin, accounting for why the side chains of E203 and E204 contribute most of the binding energy for linagliptin and FAP (for example, −6.48 and −5.05 kcal/mol for E203 and E204, respectively, for linagliptin/FAP-I-1). These results support those of the previous hydrogen bond analysis. Hence, one positively charged ammonium group must be retained to optimize linagliptin while this portion of FAPI does not contribute to the selectivity of the shared residues between FAP (E203 and E204) and DPP-4 (E205 and E206).

Analysis of the hydrogen bond between O1 in linagliptin and Y625 revealed that Y625 contributed −2.83, −2.85, and −2.84 kcal/mol to the binding energy of the linagliptin/FAP-I-1, linagliptin/FAP-I-2, and linagliptin/FAP-I-3 complexes, respectively. This hydrogen bond was more stable than the hydrogen bond between E203 and linagliptin. For the hydrogen bond formed with the backbone, the backbone of Y625 contributed approximately 1.50 kcal/mol to the three linagliptin/FAP-I systems (Figure S28). Meanwhile, the butyne group of linagliptin formed van der Waals bonds with the phenol group of Y625 ($S\Delta G_{subtotal}$ = −1.37 kcal/mol and ${\Delta E}_{vdW}$ = −1.91 kcal/mol for linagliptin/FAP-I-1). Thus, Y625 aids the stable binding of linagliptin to FAP through interactions between the side chain and backbone of Y625.

Y656 contributed more than 1 kcal/mol of energy to the linagliptin/FAP-I-1 and linagliptin/FAP-I-2 systems. However, its contribution was lower in the linagliptin/FAP-I-3 system (0.02 kcal/mol). The positive value of the electrostatic interaction energy for Y656 (Figure S29) was negated by the value of the polar solvation interaction for the first and second replicate systems (approximately 1.50 and −1.15 kcal/mol for the electrostatic and polar solvation interactions, respectively); however, those of the third replicate system did not (2.50 and −1.83 kcal/mol for the electrostatic and polar solvation interactions, respectively). Y656 was the main residue responsible for promoting the binding of linagliptin to FAP.

In addition, several aromatic amino acids contributed >1 kcal/mol to the binding energy. For example, W623 contributed −4.20, −4.22, and −4.16 kcal/mol in the linagliptin/FAP-I-1, linagliptin/FAP-I-2, and linagliptin/FAP-I-3 systems, respectively. The indole ring of W623 bonded with the quinazoline ring of linagliptin through a π–π interaction. This interaction was verified using the distance between the indole ring of W623 and the quinazoline ring of linagliptin (linagliptin/FAP-I-1: 5.00 ± 0.32 Å, linagliptin/FAP-I-2: 4.65 ± 0.37 Å, and linagliptin/FAP-I-3: 5.05 ± 0.33 Å in the 200 ns simulation; Figure S30) and the angle between the C14 atom of linagliptin, the center of the quinazoline ring of linagliptin, and center of the indole ring of W623 (linagliptin/FAP-I-1: 113.14 ± 5.50°, linagliptin/FAP-I-2: 117.91 ± 6.80°, and linagliptin/FAP-I-3: 114.05 ± 5.44° in the last 200 ns simulation; Figure S31). This is supported by the W623 backbone, which contributed −0.92 kcal/mol, and the side chain of W623, which contributed −0.56 kcal/mol, in the linagliptin/FAP-I-1 system.

The benzene ring of Y541 interacted with the pyrimidine-2,4(1H,3H)-dione ring of linagliptin through π–π bonds, resulting in Y541 contributing more than −3.84 kcal/mol in the binding of linagliptin to FAP. This interaction was confirmed by measuring the distance between the center of pyrimidine-2,4 (1H, 3H)-dione and benzene rings (linagliptin/FAP-I-1: 3.94 ± 0.23, linagliptin/FAP-I-2: 3.78 ± 0.23, and linagliptin/FAP-I-3: 3.94 ± 0.23 Å; Figure S32) and from the angles between the center of the pyrimidine-2,4 (1H, 3H)-dione ring in linagliptin, the OH atom of Y541, and the center of the benzene ring in Y541 (linagliptin/FAP-I-1: 77.02 ± 7.18°, linagliptin/FAP-I-2: 72.74 ± 7.40°, and linagliptin/FAP-I-3: 76.34 ± 8.39°; Figure S33). The main van der Waals interaction between Y541 and linagliptin was a π-π interaction, which contributed −3.56, −3.86, and −3.44 kcal/mol to the energies of linagliptin/FAP-I-1, linagliptin/FAP-I-2, and linagliptin/FAP-I-3, respectively.

In addition, R123, which has a positively charged amino group, had an unfavorable effect on linagliptin binding with FAP. The positive charges of the side chains of R123 repelled the positive charge of the amino group of linagliptin (Figure S34). The active site contained the positively charged residues R123 and the negatively charged residues E203 and E204. Thus, neutralizing electrostatic interactions is important for designing inhibitors. The long side chain of N704 may cause steric hindrance with linagliptin.

### Different linagliptin conformations

In the complexes formed by linagliptin with FAP and DPP-4, linagliptin formed different conformations. Thus, the conformation of linagliptin in its interaction with DPP-4 was evaluated in the linagliptin/FAP-II system. The linagliptin/FAP-II systems 500 ns simulations were performed in triplicate. The RMSD (Figure S35), gyration radius (Figure S36), and surface area (Figure S37) were calculated, confirming the stability of these systems and suitability for further analysis.

**Figure 5:**
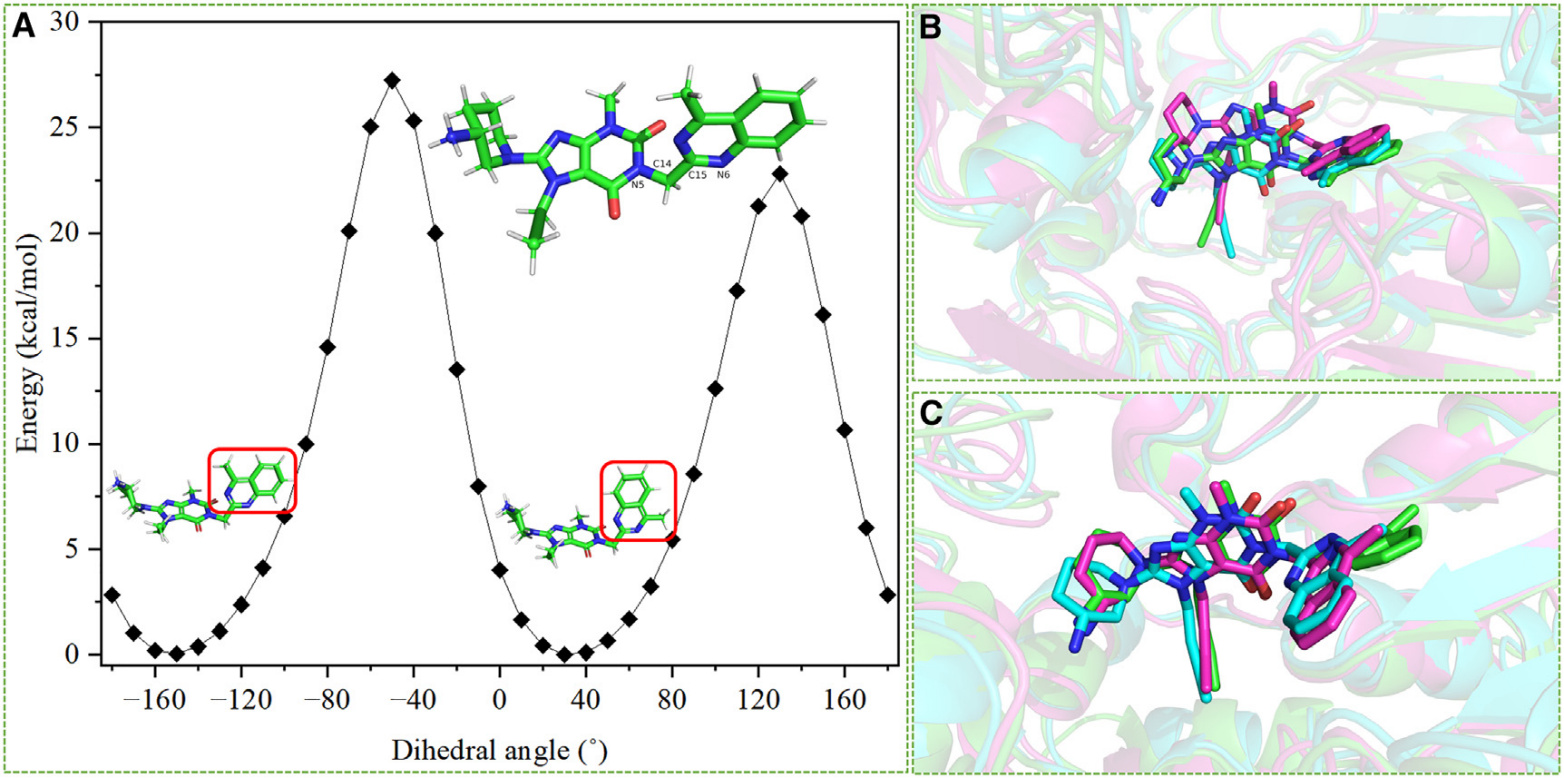
Conformations of the quinazoline group of linagliptin (A) Dihedral angles between N5, C14, C15, and N6 in linagliptin between −180° and 180° with 10° intervals. (B) Conformations of the quinazoline group of linagliptin in the 500 ns simulation in linagliptin/FAP-I-1 (green), linagliptin/FAP-I-2 (cyan), and linagliptin/FAP-I-3 (red). (C) Conformations of the quinazoline group of linagliptin in the 500 ns simulation in linagliptin/FAP-II-1 (green), linagliptin/FAP-II-2 (cyan), and linagliptin/FAP-II-3 (red). FAP, fibroblast activation protein.

Linagliptin/FAP-I and linagliptin/FAP-II differed in the quinazoline group of linagliptin (Figure S38). In the linagliptin/FAP-I systems, the dihedral angle among the N5, C14, C15, and N6 atoms in the quinazoline group was maintained, with angles of −134.91° ± 9.57°, −128.05° ± 9.05°, and −132.39° ± 11.53° in the linagliptin/FAP-I-1, linagliptin/FAP-I-2, and linagliptin/FAP-I-3 systems, respectively (Figure S39). Additionally, the structure of the quinazoline group of linagliptin was maintained in the linagliptin/FAP-II system (Figure S40). Dihedral angles between −180° and 180° with 10° intervals were screened using the wB97X functional and 6-311G (d, p) basis sets in the gas phase (detailed information is presented in the Supporting Information). Two conformations with dihedral angles of 30° and −150° had the lowest energy (Figure 5). In addition, these conformations were consistent with those of linagliptin in linagliptin/FAP-I and linagliptin/FAP-II. Therefore, these two conformations can coexist.

The other components of linagliptin did not exhibit different structures among the systems. This supports the hydrogen bond analysis results for linagliptin/FAP-I and linagliptin/FAP-II (Figure S41). The binding free energies were −12.00, −13.25, and −13.02 kcal/mol for the linagliptin/FAP-II-1, linagliptin/FAP-II-2, and linagliptin/FAP-II-3 systems, respectively (Table S11–S13). The similar binding energies between linagliptin/FAP-I and linagliptin/FAP-II systems indicated that changes in the conformation of the quinazoline group of linagliptin did not affect the binding affinity, consistent with other compounds binding with DPP-4.^48^ The different positions of quinoline on UAMC1110, the same site as the quinazoline linagliptin group, had two conformations, as evidenced by molecular docking (Figure S42). The quinoline region of UAMC1110 has been applied as a linker in chelating agents for radiopharmaceuticals, such as FAPI-04,^54^ FAPI-46,^43^ FAPI-74,^55^ NOTA-DD-FAPI,^56^ NOTA-FAPI-MB,^57^ FAPI-LM3,^25^ DOTAGA.(SA.FAPi)_2_,^58^ at C6 of the quinoline ring, DOTAGA-FAPI-FUSCC-II^59^ and OncoFAP-DOTAGA^60^ at the C8 site (Figure S43). Hence, this domain can be optimized or connected to linkers as radiopharmaceuticals.

### Design strategies

**Figure 6:**
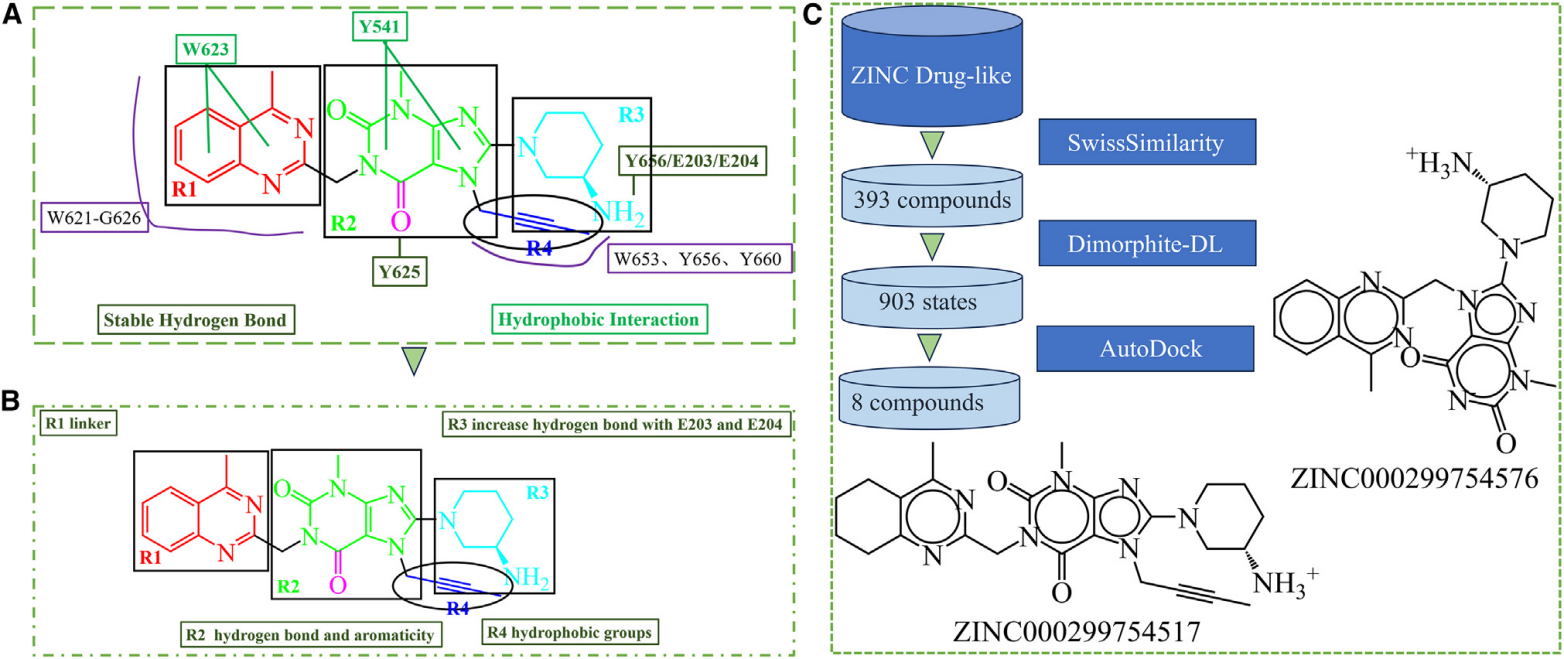
Compound design strategies based on the linagliptin structure (A) Binding model of the linagliptin/human FAP complex. (B) Design strategies based on the binding model of linagliptin/human FAP. (C) Similar small molecules identified in the ZINC database of drug-like compounds. FAP, fibroblast activation protein.

Calculations of binding free energy and MD simulations were performed to obtain a binding model for linagliptin and human FAP (Figure 6). The quinazoline group of linagliptin (R1) can form π–π interactions with W623 of FAP, in two conformations, which can be attached via a linker with a chelating agent for targeted radiopharmaceuticals (Figure S44). The R2 region, the core xanthine scaffold for those derivatized compounds, was discovered from high throughput virtual screening. The R conformation of the amino group at the piperazine ring (R3 part) can be applied to locate the conformation of linagliptin binding with FAP and contribute to the binding affinity from hydrogen bonds with E203, E204, and Y656 in the S2 subsite. The 7-butynyl substituent (R4) bound into the hydrophobic S1 pocket of FAP, essentially abolishing human ether-à-go-go-related gene channel inhibition^50^; it can be replaced with hydrophobic groups.

To identify small molecules in the ZINC drug-like molecules database with potential as FAPIs, linagliptin was selected as the template molecule because of its terminal amino group (Figure 6). A total of 393 small molecules with similarity scores >0.6 (Table S14) were identified from the ZINC database^61^ using the SwissSimilarity web tool.^62^ Additionally, the linagliptin/FAP complexes were selected from the first cluster of the three complex systems (linagliptin/FAP-I-1, linagliptin/FAP-I-2, and linagliptin/FAP-I-3) identified using cluster analysis, and the FAPs were labeled as FAP-I-1, FAP-I-2, and FAP-I-3, respectively (Figures S45–S47). The three binding models were used as the standard for molecular docking; the parameters were suitable for showing the interaction between linagliptin and FAP with an RMSD value of <1.2 Å (Figures S48–S50). Furthermore, the lowest binding energies of linagliptin/FAP-I-1, linagliptin/FAP-I-2, and linagliptin/FAP-I-3 were −11.11, −11.58, and −12.59 kcal/mol, respectively. Thus, molecular docking with these parameters was used to measure the binding affinities of the 393 small molecules for human FAP.

Using the ChemAxon pKa plugin, the primary amino group of linagliptin was shown to have one positive charge. Meanwhile, the charged amino group forms three hydrogen bonds with E203/E204/Y656. Thus, the different ionization states of the small molecules were considered at pH = 7.4 using the Dimorphite-DL software.^63^ There were 903 ionization states for the 393 small molecules. For example, there were two and four ionization states for ZINC000299754517 and ZINC000299754576, respectively (Figure S51). Next, the different ionization states were applied to molecular docking to screen for a potential FAPI. Overall, the docking scores for the 903 states with the lowest binding free energy of each interaction with the three protein conformations were selected (Table S15). The charged ammonium group played an important role in the binding of linagliptin to FAP. However, the linagliptin molecule in the ZINC database was a neutral molecule named ZINC000003820029. Meanwhile, the one positive charge state was considered in this study. The lowest binding energy of ZINC000003820029 (−11.41 and −10.70 kcal/mol for docking with one positive charge and neutral states, respectively) was greater than that of the positively charged linagliptin (−12.59 kcal/mol for docking, Figure S52). This implies that the docking score for molecular docking screening was affected by the initial conformation for molecular docking. The lowest binding energy of ZINC000003820029 (−10.70 kcal/mol) was selected as the standard for identifying potential inhibitors of human FAP. Three small molecules (ZINC000059031486, ZINC000299754517, and ZINC000299754593) exhibited greater binding affinities than ZINC000003820029 (−10.70 kcal/mol) for human FAP (Figure S53). Among these small molecules, ZINC000059031486 and ZINC000299754517 altered the amino group stereo-conformation of linagliptin, and ZINC000299754517 decreased the aromaticity of the quinazoline group to the tetrahydroquinazoline group. Moreover, these two compounds showed similar binding energies to ZINC000003820029, supporting their structural similarities (similarity scores: 1.000 for ZINC000059031486 and 0.786 for ZINC000299754517). Meanwhile, ZINC000299754593 showed better binding affinity (−10.53, −10.65, and −10.94 kcal/mol for three different ionization states) with FAP. These small molecules can be classified as having some N1 substitutions at the xanthine moiety of linagliptin (Figure S54).

Another class of small molecules had a 3-methyl-8-(4-(naphthalen-1-ylmethyl)piperazin-1-yl)-3,7-dihydro-1H-purine-2,6-dione skeleton, including ZINC000022855256, ZINC000022855258, ZINC000022421631, ZINC000022409193, ZINC000020565829, ZINC000299754576, and ZINC000009714540 (Figure S55). The side chains of this skeleton were modified with pentane, hexane, butane, isopentane, and isobutane. The binding energies of ZINC000022855256, ZINC000022855258, ZINC000022421631, ZINC000022409193, ZINC000020565829, ZINC000299754576, and ZINC000009714540 were −10.15, −10.00, −10.19, −10.01, −10.12. −10.50, and −10.25 kcal/mol, respectively. Additionally, ZINC000299754576 contained this skeleton and the key ammonium group from linagliptin. Furthermore, it had a higher binding energy (−10.50 kcal/mol) than linagliptin. This indicates that adding ammonium groups to molecular skeletons may be a potential optimization direction.

The optimal small molecules were the R1 (ZINC000299754517) and R4 (ZINC000299754576) regions of linagliptin. This indicates that the R1 and R4 regions of linagliptin can be optimized to obtain better compounds that target human FAP. Natural compounds exhibit good efficacy and low toxicity.^64^^,^^65^^,^^66^^,^^67^ Thus, compounds having similar characteristics as linagliptin, as identified from the natural compound database, may be used to unveil potential inhibitors targeting FAP.

FAP is a potential diagnostic marker and therapeutic target in solid cancers. Therefore, novel FAPIs are required. Linagliptin, a leading compound, can be used to design new or refine existing FAPIs. Here, the interaction between linagliptin and FAP is described using MD simulations and binding-free energy calculations. E203, E204, and Y656 form hydrogen bonds with the ammonium group of linagliptin. Y625 forms an unstable hydrogen bond with the carbonyl group of linagliptin. W623 interacts with the quinazoline ring of linagliptin, and Y541 interacts with the pyrimidine-2,4-dione ring of linagliptin via π-π interactions. The butyne group of linagliptin forms hydrophobic interactions with V650, Y653, Y656, and Y660. Similar small molecules identified in the ZINC drug-like compound database may serve as potential FAPIs. Eight compounds exhibit superior binding affinities than linagliptin, as determined by molecular docking. ZINC000299754517 and ZINC000299754576 are potential FAPIs. The R1 and R4 regions of linagliptin can be optimized and used to identify potential FAPIs. However, the inhibitory activity of these molecules against FAP must be verified experimentally. Furthermore, covalent targeting of FAP is a potential avenue for developing the next generation of FAPIs.

### Limitations of the study

Although the binding affinity between linagliptin and FAP has been studied using surface plasmon resonance experiments and other studies, the binding free energy is not based on isothermal titration calorimetry compared with our calculations. Further validation through simulation is necessary for verifying compounds with characteristics similar to those of linagliptin using molecular docking (e.g., molecular dynamics simulation and binding free energy calculations) and experimental (e.g., surface plasmon resonance and isothermal titration calorimetry) methodologies. Moreover, although the equilibrium state for linagliptin binding with FAP was defined, the kinetics data and effect of pH on the binding remain unclear. Therefore, thorough analyses using Gaussian accelerated molecular dynamics and constant pH molecular dynamics analyses are required.

## Resource availability

### Lead contact

Further information and requests for resources and reagents should be directed to and will be fulfilled by the lead contact, Xiaoan Li (lixiaoan@sc-mch.cn).

### Materials availability

This study did not generate new unique reagents. Further information and requests for resources and reagents should be directed to and will be fulfilled by the lead contact, Xiaoan Li (lixiaoan@sc-mch.cn).

### Data and code availability

• All data reported in this paper will be available from the lead contact upon request.
• This article does not report original code.
• Any additional information required to reanalyze the data reported in this paper is available from the lead contact upon request.

## Acknowledgments

This work was supported by the 10.13039/501100001809National Natural Science Foundation of China (no. 22203056), Foundation of Nursing Key Laboratory of Sichuan Province (no. HLKF2023(F)-4), Talent Introduction Project of Mianyang Central Hospital (2023RCYJ-003), the NHC Key Laboratory of Nuclear Technology Medical Transformation (Mianyang Central Hospital) (no. 2022HYX001 and 2020FH09), Sichuan Science and Technology Program (no. 2023YFS0470), Natural Science Foundation of Science and Technology Department of Sichuan Province (no. 2023NSFSC119), and the Mianyang Science and Technology Bureau (Minyang Science and Technology Program, grant no. 2023ZYDF073).All simulations were performed using clusters from our group, the National Supercomputing Center of Guangzhou, and the Chengdu Supercomputing Center. The authors thank Editage (www.editage.cn) for English language editing. We acknowledge HZWTECH for providing computation facilities. Mingsong Shi was partially supported by the postgraduate research opportunities program of HZWTECH (HZWTECH- PROP).

## Author contributions

Conceptualization, M.S., W.Y., and X.L.; methodology, M.S., F.W., Z.L., and Y.Y.; software, M.S., F.W., and J.C.; validation, M.S., F.W., X.Z., and D.W.; formal analysis, M.S., F.W., M.J., X.J., and J.W.; investigation, M.S., F.W., and Z.L.; resources, M.S., W.Y., and X.L.; data curation, M.S., F.W., X.C., and Y.Y.; writing—original draft preparation, M.S.; writing—review and editing, M.S., W.Y., and X.L.; visualization, M.S. and X.L.; supervision, M.S., W.Y., and X.L.; project administration, M.S. and X.L.; funding acquisition, M.S., X.J., and X.L. All authors have read and agreed to the published version of the manuscript.

## Declaration of interests

The authors declare no competing interests.

## STAR★Methods

### Key resources table

**Table:** 

REAGENT or RESOURCE	SOURCE	IDENTIFIER
**Biological samples**		

Human FAP Recombinant protein	Sangon Biotech	D145263
FAP Protein, Human, Recombinant (hFc)	TargetMol	TMPK-00373

**Chemicals, peptides, and recombinant proteins**		

Linagliptin	Bidepharm	BD161416

**Software and algorithms**		

H++	H++ online service	http://newbiophysics.cs.vt.edu/H++/
SWISS-MODEL	Expasy web server	https://swissmodel.expasy.org/
ChemAxon pKa plugin	ChemAxon	http://www.chemaxon.com
InDraw software	Integle Chemical Draw	https://www.integle.com/static/indraw
Beijing density functional (BDF)	HZWTECH	https://doi.org/10.1142/S0219633603000471
AMBER20	Amber Project	https://ambermd.org
AMBERTools21	Amber Project	https://ambermd.org/AmberTools.php
Gaussian16	Gaussian, Inc.	https://gaussian.com/
PyMOL 2.1	Schrödinger	https://sourceforge.net/projects/pymol/

### Experimental model and study participant details

No experimental models/study participants were used in the study.

### Method details

#### FAP structure preparation

The full-length human FAP (UniProt ID: Q12884) structure in the UniProt database contains 760 residues.^68^^,^^69^^,^^70^ Two crystal structures have been published in the Protein DataBank (PDB): FAP bound to substrates at 2.6 Å (PDB ID: 1Z68^51^) and linagliptin at 2.92 Å (PDB ID: 6Y0F^48^^,^^71^). Thus, the linagliptin/FAP complex crystal structure (Figure S56) was selected as the initial model to study the interactions between linagliptin and human FAP. Four chains of human FAP are bound to linagliptin in this crystal structure. The protein-protein interactions between different FAPs were not considered in this study. Chain A was similar to chains B, C, and D (Figure S57) and was, therefore, used as the initial binding model for the linagliptin/FAP complex, designated linagliptin/FAP-I. The missing residues for human FAP were modeled from chain A of the crystal structure (PDB ID: 6Y0F^48^) using the online service SWISS-MODEL.^72^ The protonation state of each residue in human FAP was determined at the physiological pH (pH 7.4) using the H++ online service,^73^^,^^74^^,^^75^^,^^76^ The disulfide bonds in FAP (C321-C332, C438-C441, C448-C466, and C643-C755) were maintained throughout the simulations.

Using the PDB, the crystal structures of two linagliptin complexes were identified: linagliptin bound to DPP-4 (PDB ID: 2RGU^50^) and FAP (PDB ID: 6Y0F^48^). Owing to the highly conserved binding pockets of DPP-4 and FAP, they exhibited a similar binding pattern with linagliptin (Figure S57). However, the conformation of the quinazoline-2-yl-methyl group differed between DPP-4 and FAP. Thus, it was postulated that linagliptin formed different conformations when binding to FAP compared with the linagliptin/DPP-4 complex. The other linagliptin/FAP complex (i.e., linagliptin/FAP-II) was constructed by aligning the structures identified for FAP with those of the linagliptin/DPP-4 complex (PDB ID: 2RGU^50^). First, based on the superposition of the protein, the FAP coordinates were moved to the linagliptin/DPP-4 system. Second, the coordinates of linagliptin were obtained from the linagliptin/DPP-4 structure. Subsequently, the linagliptin coordinates were fitted to the active site of human FAP to form the linagliptin/FAP-II complex (Figure S58).

#### Linagliptin structure preparation

The structure of linagliptin was downloaded from the PDB (Compound ID: 356). The charge states of linagliptin at different pH values were derived from the amine groups using the ChemAxon pKa plugin (Calculator Plugins, Marvin 22.13, 2022; ChemAxon, http://www.chemaxon.com, Figure S59). As physiological environments have a pH of 7.4, microspecies #2, which has one positive charge on the primary amino group, was selected as a suitable conformation of linagliptin, supported by its pKa of 1.9 and 8.6 for the protonation of the quinazoline and primary amino groups, respectively, determined from experimental results.^50^ The two-dimensional structure of linagliptin in the microspecies #2 conformation was constructed using InDraw software (Integle Chemical Draw) and transformed into a three-dimensional structure using Openbabel 2.4.0.^77^ The three-dimensional structure of the selected linagliptin conformation was optimized at the B3LYP/6-311G (d, p) level.^66^^,^^78^^,^^79^^,^^80^^,^^81^^,^^82^^,^^83^^,^^84^^,^^85^^,^^86^ The same level was used to evaluate the predicted frequency numbers to confirm whether the species’ conformation was stable. These calculations were performed using the four-component Beijing density functional (BDF) program package.^87^^,^^88^^,^^89^^,^^90^

#### Molecular dynamics simulation

The standard force field parameters for small molecules and linagliptin have not yet been published. Therefore, the general amber force field (GAFF, version 2)^91^ was used to construct the force-field parameters for linagliptin. The charge of the partial atoms in linagliptin was obtained using the restrained electrostatic potential protocol at the HF/6-31G theory level.^92^ The antechamber module in AMBERTools21^93^ and Gaussian 16 software^94^ were used to construct the force field, whereas the standard protein force field was applied for human FAP (AMBER ff19SB force field^95^). Seven Na^+^ ions were added to the complex to neutralize the charge of the linagliptin/FAP complex. The optimal point charge water molecule^96^ was employed to solvate the linagliptin/FAP systems with a 15 Å distance in the cuboid box. Meanwhile, 95 NaCl molecules were solvated in the system box. Subsequently, 36,278 water molecules were added to the linagliptin/FAP system. The final linagliptin/FAP complex system included 721 residues of human FAP, one small molecule of linagliptin, 102 Na^+^ ions, 95 Cl^-^ ions, and 36,278 water molecules (Table S16). In the initial optimization of the linagliptin/FAP complex, the steepest descent and conjugate gradient methods were used, where all solute molecules were not minimized. In the second step, using conjugate gradients without restriction, the entire system was minimized. Over 200 ps, Langevin dynamics were used to increase the temperature of the system from 0 to 300 K following the two minimization steps. Using isotropic position scaling, the temperature was maintained at 1 bar for 200 ps. Afterward, a pre-equilibration was conducted at 300 K and 1 bar for 200 ps within a fixed nitrogen atom, pressure, and temperature ensemble (isothermal-isobaric). The entire system was simulated for 500 ns using MD simulations following data collection and analysis. Simulations were conducted using AMBER20 and AMBERTools21.^93^ The *CPPTRAJ* module^97^^,^^98^ was used to analyze the data. Three replicates were performed for each experiment.

#### Binding free energy calculation

An MM/GBSA method was used to calculate linagliptin and human FAP binding free energies,^99^^,^^100^ which has been widely applied to calculate the binding affinities between enzymes and their ligands.^101^^,^^102^^,^^103^^,^^104^^,^^105^^,^^106^ A detailed description of the MM/GBSA framework has already been provided.^107^^,^^108^^,^^109^ The MM/GBSA method was used to calculate the energy terms for the linagliptin/FAP complex based on the last 200 ns of the MD simulation. MM/GBSA binding energy decomposition was employed to identify the contribution energy of each residue to linagliptin binding FAP.^110^ The MMPBA.py tool in AMBERTools21 has been used for energy calculation.^93^ The supporting information for calculation methods contains more information about the binding free energy calculations. The methods employed in this work are schematically depicted in Figure S60.

#### Trajectory analysis

The trajectories from three independent simulations of each system were analyzed using the tools available in the AMBERTools21 software package. As defined, a hydrogen bond occurs when the distance between the acceptor and donor atoms is greater than 3.5, and the internal angle between the acceptor, hydrogen, and donor atoms is greater than 120°. Structural visualization was performed using PyMOL software.^111^

#### Surface plasmon resonance experiments

Human FAP (27–760) was obtained from TargetMol (TargetMol Chemicals Inc., Boston, MA, USA). Linagliptin was purchased from Bide Pharmatech, Ltd. (Shanghai, China). Immobilization of recombinant human FAP on CM5 chip was performed in 10 mM sodium acetate at pH 5.0. Binding studies were conducted at 25°C in 20 mM Tris (pH as indicated), 150 mM NaCl, 0.05% (v/v) Tween 20, and 1% (v/v) DMSO with surface plasmon resonance (SPR) with a Biacore T200. The linagliptin concentrations were 15.6, 31.25, 62.5, 125, 250, and 500 nM for the FAP binding studies. At a flow rate of 30 L/min, FAP binding was determined with a 120 s association time and 180 s. The Biacore T200 Evaluation software (version 3.0; GE Healthcare) was employed to analyze kinetic parameters.

### Quantification and statistical analysis

There are no quantification or statistical analyses to include in this study.

## Supplemental information

Document S1. Figures S1–S60 and Tables S1–S13 and S16

Table S14. For similarity score for the screening small molecules which related to Figure 6

Table S15. For docking score for the similarity small molecules which related to Figure 6
