# Supplementary material for: Elucidating the linagliptin and fibroblast activation protein binding mechanism through molecular dynamics and binding free energy analysis
Source: iScience. 2024 Nov 13;27(12):111368. doi: 10.1016/j.isci.2024.111368 (PMC11629334; doi:10.1016/j.isci.2024.111368)
Supplement: Document S1. Figures S1–S60 and Tables S1–S13 and S16 [file mmc1.pdf]

**iScience, Volume *27***

## Supplemental information

# Elucidating the linagliptin and fibroblast activation protein binding mechanism through molecular dynamics and binding free energy analysis

**Mingsong Shi, Fang Wang, Zhou Lu, Yuan Yin, Xueting Zheng, Decai Wang, Xianfu Cai, Meng Jing, Jianjun Wang, Junxian Chen, Xile Jiang, Wenliang Yu, and Xiaoan Li**

**Elucidating the linagliptin and human fibroblast activation protein binding mechanism through molecular dynamics and binding free energy analysis**

Mingsong Shi[1,2,#], Fang Wang[2,#], Zhou Lu[1], Yuan Yin[1], Xueting Zheng[1], Decai Wang[3], Xianfu Cai[3], Meng Jing[4], Jianjun Wang[3], Junxian Chen[5], Xile Jiang[2], Wenliang Yu[6,*], Xiaoan Li[1,7,*]

[1] NHC Key Laboratory of Nuclear Technology Medical Transformation, Mianyang Central Hospital, School of Medicine, University of Electronic Science and Technology of China, Mianyang, Sichuan, 621099, China
[2] Department of Clinical Nutrition, Innovation Center of Nursing Research, Nursing Key Laboratory of Sichuan Province, West China Hospital, Sichuan University, Chengdu, Sichuan, 610041, China
[3] Department of Hepatobiliary Surgery, Mianyang Central Hospital, School of Medicine, University of Electronic Science and Technology of China, Mianyang, Sichuan, 621099, China
[4] Department of Pathology, Mianyang Central Hospital, School of Medicine, University of Electronic Science and Technology of China, Mianyang, Sichuan, 621099, China
[5] Key Laboratory of General Chemistry of the National Ethnic Affairs Commission, School of Chemistry and Environment, Southwest Minzu University, Chengdu 610041, Sichuan, China
[6] Department of Obstetrics and Gynecology, Mianyang Central Hospital, School of Medicine, University of Electronic Science and Technology of China, Mianyang, Sichuan, 621099, China
[7] Department of Gastroenterology, Mianyang Central Hospital, School of Medicine, University of Electronic Science and Technology of China, Mianyang, Sichuan, 621099, China

[#] These authors have contributed equally to this work.
[*] Correspondence: lixiaoan@sc-mch.cn (X.L.); 13778079235@163.com (W.Y.)

Lead contact
Further information and requests for resources and reagents should be directed to and will be fulfilled by the Lead Contact, Xiaoan Li (lixiaoan@sc-mch.cn).

Contents

Method ........................................................................................................S1

Method S1: Molecular dynamics simulations, related to Figure 1 ..........................S1

Method S2: Cluster analysis, related to Figures S45-S50.......................................S2

Method S3: Binding free energy calculation, related to Figure 1 and Figure 4.......S2

Method S4: Surface plasmon resonance experiments, related to Figure S25..........S3

Method S5: Enzymatic assay, related to Figure S26................................................S4

Method S6: Molecular docking, related to Figure 1 ................................................S4

Method S7: Density functional theory, related to Figure 5......................................S5

Reference ..................................................................................................S5

Figure S1. Structures for FAP inhibitors.......................................................S9

Figure S2. Root mean square deviation (RMSD) value of heavy atoms of backbone for human FAP and no-hydrogen atoms of linagliptin along 500 ns MD simulation for three linagliptin/FAP-I complex systems.........................................................S10

Figure S3. Snapshots of the linagliptin/FAP-I-1 along the dynamic simulation time for 400-500 ns..........................................................................................S11

Figure S4. Surface area for linagliptin binding with FAP ........................................S12

Figure S5. Gyration radius for linagliptin binding with FAP ....................................S13

Figure S6. Dimer structure of human FAP ...............................................................S14

Figure S7. Root mean square deviation (RMSD) value of heavy atoms of backbone for human FAP and BD4 domain of FAP along 500 ns MD simulation for three linagliptin/FAP-I complex systems.........................................................S15

Figure S8. Root mean square deviation (RMSD) value of heavy atoms of backbone for human FAP along 500 ns MD simulation for apo-FAP systems................................S16

Figure S9. RMSF variations for Cα atom of human FAP for linagliptin/FAP-I and Apo-FAP systems from the 500 ns MD simulation .........................................................S17

Figure S10. Snapshots of the linagliptin/FAP-I-1 along the dynamic simulation time for 100, 200, 300, 400, and 500 ns ......................................................................S18

Figure S11. Snapshots of the linagliptin/FAP-I-2 along the dynamic simulation time for 100, 200, 300, 400, and 500 ns ....................................................................S19

Figure S12. Snapshots of the linagliptin/FAP-I-3 along the dynamic simulation time for 100, 200, 300, 400, and 500 ns ....................................................................S20

Figure S13. Snapshots of the apo-FAP-1 along the dynamic simulation time for 100, 200, 300, 400, and 500 ns ..........................................................................S21

Figure S14. Snapshots of the apo-FAP-2 along the dynamic simulation time for 100, 200, 300, 400, and 500 ns ..........................................................................S22

Figure S15. Snapshots of the apo-FAP-3 along the dynamic simulation time for 100, 200, 300, 400, and 500 ns ..........................................................................S23

Figure S16. Frames of linagliptin/FAP-I complex systems for initial and 500$^{th}$ ns...S24

Figure S17. Statistical hydrogen bond number profile along the 500-ns MD simulation for linagliptin/FAP-I..........................................................................................S25

Figure S18. Hydrogen bond parameters for hydrogen bond between LIG@O1 and Y625@N for linagliptin/FAP-I complex system .....................................................S26

Figure S19. Hydrogen bond parameters for hydrogen bond between E203@OE2 and LIG@N3 for linagliptin/FAP-I complex system .....................................................S27

Figure S20. Hydrogen bond parameters for hydrogen bond between E204@OE1 and LIG@N3 for linagliptin/FAP-I complex system .....................................................S28

Figure S21. Hydrogen bond parameters for hydrogen bond between E204@OE2 and LIG@N3 for linagliptin/FAP-I complex system .....................................................S29

Figure S22. Hydrogen bond parameters for hydrogen bond between Y656@OH and LIG@N3 for linagliptin/FAP-I complex system .....................................................S30

Figure S23. Activity and structure for piperazine and 3-aminopiperidine groups at C8 of xanthine moiety ...................................................................................S31

Figure S24. Activity and structure for the R and S conformations for the 3-aminopiperidine groups at C8 of the xanthine moiety .............................................S32

Figure S25. Linagliptin binding with human FAP from surface plasmon resonance experiments ..........................................................................................S33

Figure S26. Inhibition activity for linagliptin with FAP ..........................................S34

Figure S27. Energy for linagliptin binding with FAP for linagliptin/FAP-I systems S35

Figure S28. Energy decomposition with backbone and sidechain for linagliptin/FAP-I systems....S36

Figure S29. Energy decomposition for linagliptin/FAP-I systems....S37

Figure S30. Distance between quinazoline ring of linagliptin and indole ring of W623 for linagliptin/FAP-I systems although the 500 ns MD simulation....S38

Figure S31. Angle among quinazoline ring of linagliptin, indole ring of W623, and C14 atom of linagliptin for linagliptin/FAP-I systems although the 500 ns MD simulation ....S39

Figure S32. Distance between ring B of linagliptin and benzene ring of Y541 for linagliptin/FAP-I systems although the 500 ns MD simulation....S40

Figure S33. Angle among ring B of linagliptin, OH atom of Y541, and benzene ring of Y541 for linagliptin/FAP-I systems although the 500 ns MD simulation....S41

Figure S34. R123 and N704 of FAP interact with linagliptin....S42

Figure S35. Root mean square deviation (RMSD) vs Time plot for the 500 ns MD simulation on linagliptin/FAP-II....S43

Figure S36. Gyration radius vs Time plot for the 500 ns MD simulation on linagliptin/FAP-II....S44

Figure S37. Surface area vs Time plot for the 500 ns MD simulation on linagliptin/FAP-II....S45

Figure S38. Conformations for quinazoline group of linagliptin in linagliptin/FAP complex systems....S46

Figure S39. Dihedral angle among N5, C14, C15 and N6 atoms for linagliptin/FAP-I ....S47

Figure S40. Dihedral angle among N5, C14, C15 and N6 atoms for linagliptin/FAP-II ....S48

Figure S41. Hydrogen bond analysis for linagliptin/FAP-II system....S49

Figure S42. Conformation analysis for UAMC1110 binding with FAP from molecular docking....S50

Figure S43. Quinazoline ring of UAMC1110 are applied to design targeting FAP

radiopharmaceutical ........................................................................................S51
Figure S44. Binding model for linagliptin with human FAP ....................................S52
Figure S45. Cluster analysis for linagliptin/FAP-I-1 system ....................................S53
Figure S46. Cluster analysis for linagliptin/FAP-I-2 system ....................................S54
Figure S47. Cluster analysis for linagliptin/FAP-I-3 system ....................................S55
Figure S48. Docking analysis for linagliptin/FAP-I-1 system ..................................S56
Figure S49. Docking analysis for linagliptin/FAP-I-2 system ..................................S57
Figure S50. Docking analysis for linagliptin/FAP-I-3 system ..................................S58
Figure S51. Ionization states of ZINC000299754517 and ZINC000299754576 from prediction with Dimorphite-DL ..................................................................S59
Figure S52. Structure and docking score for linagliptin with different ionization states ....................................................................................................S60
Figure S53. Structure and docking score for the top four compounds ......................S61
Figure S54. Class one small molecules as potential FAP inhibitors ..........................S62
Figure S55. Class two small molecules as potential FAP inhibitors ..........................S63
Figure S56. Crystal structure for linagliptin binding with human fibroblast-activation protein ....................................................................................................S64
Figure S57. Conformations of linagliptin binding with FAP and DPP-4 ..................S65
Figure S58. Method for construction linagliptin/FAP-II system ..............................S66
Figure S59. Different charged state of linagliptin....................................................S67
Figure S60. Method for linagliptin/FAP systems.....................................................S68
Table S1. RMSD value of the FAP and linagliptin for the linagliptin/FAP complex systems although 500 ns MD simulations ..............................................................S69
Table S2. Statistical hydrogen bond distance between linagliptin and FAP the linagliptin/FAP complex systems although 500 ns MD simulations .........................S70
Table S3. Statistical hydrogen bond angle between linagliptin and FAP the linagliptin/FAP complex systems although 500 ns MD simulations .........................S71
Table S4. Binding free energies, decomposition and electrostatic interactions (**Eele**),

van der Waals interactions ($\mathbf{EvdW}$), solvation free energies ($\mathbf{Epolar}$), nonpolar solvation energies ($\mathbf{Enonpolar}$), and entropy ($\mathbf{TStotal}$)[#] of the linagliptin/FAP-I systems........................................................................................................S72

Table S5. Binding free energies ($\Delta\mathbf{Gbindcal}$) for linagliptin/FAP-I-1 complex system ........................................................................................................S73

Table S6. Binding free energies ($\Delta\mathbf{Gbindcal}$) for linagliptin/FAP-I-2 complex system ........................................................................................................S74

Table S7. Binding free energies ($\Delta\mathbf{Gbindcal}$) for linagliptin/FAP-I-3 complex system ........................................................................................................S75

Table S8. Free energy decomposition for the FAP-linagliptin-I-1 complex on the individual residue basis, where decomposition is performed in terms of the contributions from van der Waals energy, the electrostatic interaction energy, the nonpolar solvation free energy, the polar solvation free energy, the backbone energy, and the side chain energy ..........................................................................S76

Table S9. Free energy decomposition for the FAP-linagliptin-I-2 complex on the individual residue basis, where decomposition is performed in terms of the contributions from van der Waals energy, the electrostatic interaction energy, the nonpolar solvation free energy, the polar solvation free energy, the backbone energy, and the side chain energy ..........................................................................S77

Table S10. Free energy decomposition for the FAP-linagliptin-I-3 complex on the individual residue basis, where decomposition is performed in terms of the contributions from van der Waals energy, the electrostatic interaction energy, the nonpolar solvation free energy, the polar solvation free energy, the backbone energy, and the side chain energy ..........................................................................S78

Table S11. Binding free energies ($\Delta\mathbf{Gbindcal}$) for linagliptin/FAP-II-1 complex system ........................................................................................................S79

Table S12. Binding free energies ($\Delta\mathbf{Gbindcal}$) for linagliptin/FAP-II-2 complex system ........................................................................................................S80

Table S13. Binding free energies ($\Delta\mathbf{Gbindcal}$) for linagliptin/FAP-II-3 complex system ........................................................................................................S81

Table S16. System information for linagliptin binding with FAP ...........................S82

**Method**

**Method S1: Molecular dynamics simulations, related to Figure 1**

To correctly describe the force field of linagliptin, the general Amber force field (GAFF2)[1] generation procedure was used. The geometry structure was optimized at B3LYP/6-311G (d, p) level of theory using Beijing four-component density functional (BDF)program package[2, 3]. To obtain the partial atomic charges, the restrained electrostatic potential (RESP) protocol[4] was employed at the HF/6-31G* level of theory. The force field parameters were generated using the Antechamber module. Meanwhile, the AMBER ff19SB force field[5] was used to create topology parameters of protein human FAP. The missing residues for human FAP were modeled by online service SWISS-MODEL[6] from the chain A of crystal structure (PDB ID: 6Y0F[7]). The protonation state of every residue in human FAP was determined with at physiological pH value (pH = 7.4) using the H++ online service[8-11]. Those disulfide bonds for FAP (C321-C332, C438-C441, C448-C466, C643-C755) were maintained for simulations. Firstly, the complex systems were dissolved in a OPC water[12] with cuboid box, and the total box size was about 103 Å × 116 Å × 117 Å. Results from Tian[5] showed that ff19SB pairs best with the more accurate water model OPC. The systems were neutralized using the sodium chloride ions. The solute atom, solvent water, and sodium chloride ions of the final system was list in Table S15. The periodic boundary conditions were applied to avoid edge effects and a cut-off radius of 12 Å was employed for van der Waals interactions. The particle mesh Ewald (PME) algorithm[13] also was used in calculating the long-range electrostatic interactions. The SHAKE algorithm[14] was used to constrain the covalent bond involved with hydrogen atoms. To reduce the effect of unfavorable interactions produced by solvents and ion, the system was subjected to 9000 steps of steepest descent method and then 1000-step conjugate gradient, while fixing all the solute molecules at the initial position. Then the 10000-step conjugate gradient method was used to optimize the whole system including solute molecule and solvent molecule. After the first two-step minimization of the system, the overall system temperature was heated from 0 K to 300 K in 200 ps with Langevin dynamics[15, 16]with the collision frequency g = 2.0 $ps^{-1}$. The pressure was then kept at 1 bar in 200 ps with isotropic position scaling[17] and temperature at 300 K. After that, the system was equilibrated at 300 K and 1 bar within the NPT (isothermal-isobaric) ensemble with 200 ps. Subsequently, the entire system underwent the 500 ns molecular dynamics simulation for final data collection and analyses. The integration step size was set to 2 fs throughout

the molecular dynamics. The random seed was based on the current date and time for every step. Especially, the random seed was generated every ns simulation in the 500 ns MD simulations. All dynamics were performed using the CUDA version of the PMEMD in AMBER 20[18]. The *CPPTRAJ* module[19, 20] was used to analysis the data from the MD trajectories.

Method S2: Cluster analysis, related Figures to S45-S50

Cluster analysis is a general unsupervised technique for finding patterns within data. In this work, the root mean-square deviation (RMSD)-based clustering was performed with *ptraj*[19, 20], a simulation analysis tools implemented in AMBERTools21[21]. As one of the most popular clustering algorithms, the average linkage cluster algorithm was used herein. Each cluster contained a representative structure whose RMSD was equidistant to all other cluster members. Structures were collected by sampling at 100 ps intervals in the last 200 ns simulation with 2000 frames, and cluster analysis was based on the heavy atoms of linagliptin/FAP complex to generate some clusters with epsilon = 2.0 Å.

Method S3: Binding free energy calculation, related to Figure 1 and Figure 4

In addition to qualitative analysis of ligand/protein binding, quantitative analysis is also important in determining the binding affinity between ligands and proteins. Several methods, such as molecular mechanics/Poisson Boltzmann (or generalized Born) surface area (MM/PBSA or MM/GBSA)[22, 23], solvated interaction energy[24], linear interaction energy[25-27], free energy pathway method[28], and linear response approximation[29], have been developed to estimate the absolute binding free energy between inhibitors and their target proteins. The MM/GBSA approach is a timesaving and efficient method to evaluate the binding free energy between inhibitors and proteins[30-34]. In this study, only a short description of the calculation of the binding free energy ($\Delta G_{binding}$) from the MM/GBSA method is provided in the following formulae:

$$\Delta G_{binding} = G_{complex} - G_{protein} - G_{ligand} \quad (1)$$

$$G = E_{gas} + E_{sol} - TS \quad (2)$$

$$E_{gas} = E_{int} + E_{vdW} + E_{ele} \quad (3)$$

$$E_{sol} = E_{polar} + E_{nonpolar} \quad (4)$$

$E_{nonpolar}$= γ·SA + b (5)

$G_{complex}$, $G_{protein}$, and $G_{ligand}$ denote the free energies of linagliptin/FAP, FAP, and linagliptin, respectively. $G$ can be decomposed into enthalpy ($H = E_{gas} + E_{sol}$) and entropy ($TS$). The molecular mechanical energies ($E_{gas}$) can also be summarized from the intramolecular energy ($E_{int}$), van der Waals forces ($E_{vdW}$), and electrostatic forces ($E_{ele}$). Meanwhile, the contributions of $E_{int}$, $E_{vdW}$, and $E_{ele}$ can be obtained through the statistical average based on molecular mechanics. In addition, solvation free energy ($E_{sol}$) can be divided into polar solvation ($E_{polar}$) and nonpolar solvation energies ($E_{nonpolar}$). $E_{nonpolar}$ is obtained from the favorable van der Waals interactions between the solute and solvent and the unfavorable cost of surface formation. $E_{nonpolar}$ can be calculated using equation (5), where $\gamma$ = 0.0072 kcal/Å$^2$ and $b$ = 0.0 kcal/mol. The linear combination of pairwise overlaps (LCPO) method[35] was employed to estimate the solvent accessible surface area (*SA*). However, the $E_{polar}$ contribution was calculated from the GB equation.[36, 37] The dielectric constant for the ligand/protein and water were set to 1 and 80, respectively. In addition, normal model analysis was used to calculate the entropy contribution. Thousand snapshots were extracted from the last 100 ns MD trajectory to calculate the statistical average of the MM/GBSA method. The 100 snapshots from the last 100 ns MD trajectory were employed to estimate the entropy contribution.

The binding free energy between linagliptin and each residue of FAP was also decomposed for van der Waals ($\Delta G_{vdW}$), electrostatic ($\Delta G_{ele}$), polar solvation ($\Delta G_{polar}$), and nonpolar solvation energies ($\Delta G_{nonpolar}$) using the MM/GBSA method, and the same parameters were applied in the binding free energy calculation. In addition, the free energy decomposition was calculated for the backbone ($B\Delta G_{subtotal}$) and sidechain energies ($S\Delta G_{subtotal}$) for each residue.

**Method S4: Surface plasmon resonance experiments, related to Figure S25**

Human FAP (27-760) protein was obtained from TargetMol (TargetMol Chemicals Inc., Boston，USA). Linagliptin has been purchased from Bidepharm (Bide pharmatech ltd., Shanghai, China). The surface plasmon resonance experiment is referenced article[7, 38]. Experiments were performed in at 25 °C on a BIAcore T200 using CM5 sensor chips,

and data were analysed using BIAcore T200 Evaluation software (GE Healthcare) following the manufacturer's instruction. In brief, a cell on the CM5 sensor chip was activated with a mixture of 200 μM 1-ethyl-3-(3-dimethylaminopropyl)carbodiimide (EDC) and 50 μM N-hydroxysuccinimide (NHS) at 10 μl min–1 for 420s. A total of 50 μl of protein by mixing with 180 μl of 10 mM sodium acetate solution, pH 5.0, was then immobilized on the surface of the cell at 10 μl min–1 for 420s for two repetitive runs. The cell was then blocked with 1 M ethanolamine (10 μl min–1 for 420s). A neighbouring aisle that served as a reference was similarly activated and blocked, except that PBS adjusted to pH 5.0 was used for immobilization. Both of the aisle were then equilibrated with PBS. Molecule stock solution was diluted to a series of concentrationsin PBS, and was flowed at 10 μl min–1 for 150 s in each run. At the end of each flow, cells were regenerated for 5 min with 10 mM glycine-HCl (pH 2.0) solution at 10 μl min–1. Data from the sample cell were collected using BIAcore T200 Control software (v. 2.0, GE Healthcare), and were subtracted by those from the reference cell. Association and dissociation constants were obtained by global fitting of the data to a 1:1 Langmuir binding model using BIAcore T200 Evaluation software (v.2.0, GE Healthcare). Data were exported to Origin 2024b software (OriginLab) with multicycle kinetic for generating the final figures.

**Method S5: Enzymatic assay, related to Figure S26**

The human FAP recombinant protein (Cat. No. D145263) was purchased from Sangon Biotech (Shanghai) Co., Ltd. The FAP was diluted 2,500-folds with assay buffer (25 mM Tris-HCl, 250 mM NaCl, pH 7.4) prior to use. A 10 mM stock solution in dimethylformamide of the substrate (Z-Gly-Pro-AMC) was diluted 250-folds with assay buffer before performing the assay. The assay itself was performed in black flat-bottom 96-well plates by mixing 25 μl of appropriate compound dilutions in assay buffer (compound stock solutions in DMSO) with 25 μL of the diluted substrate (final concentration in the assay 40 μM) and 50 μl of the diluted FAP. The plate was then incubated at 37°C for 1 h and fluorescence was measured at excitation/emission wavelengths of 360/460 nm using a BioTek Synergy LX.

**Method S6: Molecular docking, related to Figure 1**

Based on the receptor protein of FAP obtained by cluster analysis, the receptor-ligand complex structure can be prepared by the molecule docking simulation, which

has been widely applied[39, 40]. The protein was pretreated through the AutoDockTools 1.5.6 (ADT)[41], including Gasteiger charge, adjustment of unreasonable atomic overlap, and so on. The structure of linagliptin and other small molecules was optimized by PM3 force field[42-44] by MOPAC2016[45]. A grid map with 0.375 Å grid spacing was generated using AutoGrid based on the center of original linagliptin from cluster analysis linagliptin/FAP complex systems. The box size was determined from the default method with size of small molecules. The 100 conformations of each system were finally generated with Lamarckian genetic algorithm implemented in AutoDock4.2[46]. At the end, the conformation with lowest binding energy of each docking models was remained.

**Method S7: Density functional theory, related to Figure 5**

The structure of linagliptin was extracted from the representative conformation of the fist cluster for linagliptin/FAP-I-1 systems. The geometry of linagliptin is not continue optimize in the density functional theory calculation. The dihedral angle among N5, C14, C15, and N6 is set between $-180^{\circ}$ and $180^{\circ}$ and interval is $10^{\circ}$. Single point energy calculations for those 37 conformations of linagliptin were carried out using Beijing four-component density functional (BDF) program package[2, 3]. The single point energy was calculated using the wB97X functional with a mixed basis set 6-311G (d, p) in the gas phase.

**Reference**

1. Wang, J. M.; Wolf, R. M.; Caldwell, J. W.; Kollman, P. A.; Case, D. A., Development and testing of a general amber force field. *J. Comput. Chem.* **2004,** *25* (9), 1157-1174.

2. Zhang, Y.; Suo, B. B.; Wang, Z. K.; Zhang, N.; Li, Z. D.; Lei, Y. B.; Zou, W. L.; Gao, J.; Peng, D. L.; Pu, Z. C.; Xiao, Y. L.; Sun, Q. M.; Wang, F.; Ma, Y. T.; Wang, X. P.; Guo, Y.; Liu, W. J., BDF: A relativistic electronic structure program package. *J. Chem. Phys.* **2020,** *152* (6), 11.

3. Liu, W. J.; Wang, F.; Li, L. M., The Beijing Density Functional (BDF) program package: Methodologies and applications. *J. Theor. Comput. Chem.* **2003,** *2* (2), 257-272.

4. Bayly, C. I.; Cieplak, P.; Cornell, W. D.; Kollman, P. A., A well-behaved electrostatic potential based method using charge restraints for deriving atomic charges: the RESP model. *J. Phys. Chem.* **1993,** *97* (40), 10269-10280.

5. Tian, C.; Kasavajhala, K.; Belfon, K. A. A.; Raguette, L.; Huang, H.; Migues, A. N.; Bickel, J.; Wang, Y. Z.; Pincay, J.; Wu, Q.; Simmerling, C., ff19SB: Amino-acid-specific protein backbone parameters trained against quantum mechanics energy surfaces in solution. *Journal of Chemical Theory and Computation*

**2020,** *16* (1), 528-552.

6. Waterhouse, A.; Bertoni, M.; Bienert, S.; Studer, G.; Tauriello, G.; Gumienny, R.; Heer, F. T.; de Beer, T. A. P.; Rempfer, C.; Bordoli, L.; Lepore, R.; Schwede, T., SWISS-MODEL: homology modelling of protein structures and complexes. *Nucleic Acids Res.* **2018,** *46* (W1), W296-W303.

7. Schnapp, G.; Hoevels, Y.; Bakker, R. A.; Schreiner, P.; Klein, T.; Nar, H., A single second shell amino acid determines affinity and kinetics of linagliptin binding to type 4 dipeptidyl peptidase and fibroblast activation protein. *ChemMedChem* **2021,** *16* (4), 630-639.

8. Anandakrishnan, R.; Aguilar, B.; Onufriev, A. V., H++3.0: automating pK prediction and the preparation of biomolecular structures for atomistic molecular modeling and simulations. *Nucleic Acids Res.* **2012,** *40* (W1), W537-W541.

9. Myers, J.; Grothaus, G.; Narayanan, S.; Onufriev, A., A simple clustering algorithm can be accurate enough for use in calculations of pKs in macromolecules. *Proteins* **2006,** *63* (4), 928-938.

10. Gordon, J. C.; Myers, J. B.; Folta, T.; Shoja, V.; Heath, L. S.; Onufriev, A., H++: a server for estimating pK(a)s and adding missing hydrogens to macromolecules. *Nucleic Acids Res.* **2005,** *33*, W368-W371.

11. Bashford, D.; Karplus, M., pKa of ionizable groups in proteins: Atomic detail from a continuum electrostatic model. *Biochemistry* **1990,** *29* (44), 10219-10225.

12. Izadi, S.; Anandakrishnan, R.; Onufriev, A. V., Building water models: A different approach. *J. Phys. Chem. Lett.* **2014,** *5* (21), 3863-3871.

13. Darden, T.; York, D.; Pedersen, L., Particle mesh Ewald: An N · log(N) method for Ewald sums in large systems. *The Journal of Chemical Physics* **1993,** *98* (12), 10089-10092.

14. Ryckaert, J.-P.; Ciccotti, G.; Berendsen, H. J. C., Numerical integration of the cartesian equations of motion of a system with constraints: molecular dynamics of n-alkanes. *J. Comput. Phys.* **1977,** *23* (3), 327-341.

15. Feller, S. E.; Zhang, Y. H.; Pastor, R. W.; Brooks, B. R., Constant pressure molecular dynamics simulation: The Langevin piston method. *J. Chem. Phys.* **1995,** *103* (11), 4613-4621.

16. Martyna, G. J.; Tobias, D. J.; Klein, M. L., Constant pressure molecular dynamics algorithms. *J. Chem. Phys.* **1994,** *101* (5), 4177-4189.

17. Berendsen, H. J. C.; Postma, J. P. M.; Vangunsteren, W. F.; Dinola, A.; Haak, J. R., Molecular dynamics with coupling to an external bath. *J. Chem. Phys.* **1984,** *81* (8), 3684-3690.

18. D.A. Case, K. B., I.Y. Ben-Shalom, S.R. Brozell, D.S. Cerutti, T.E. Cheatham, III, V.W.D. Cruzeiro, T.A. Darden, R.E. Duke, G. Giambasu, M.K. Gilson, H. Gohlke, A.W. Goetz, R. Harris, S. Izadi, S.A. Izmailov, K. Kasavajhala, A. Kovalenko, R. Krasny, T. Kurtzman, T.S. Lee, S. LeGrand, P. Li, C. Lin, J. Liu, T. Luchko, R. Luo, V. Man, K.M. Merz, Y. Miao, O. Mikhailovskii, G. Monard, H. Nguyen, A. Onufriev, F.Pan, S. Pantano, R. Qi, D.R. Roe, A. Roitberg, C. Sagui, S. Schott-Verdugo, J. Shen, C. Simmerling, N.R.Skrynnikov, J. Smith, J. Swails, R.C. Walker, J. Wang, L. Wilson, R.M. Wolf, X. Wu, Y. Xiong, Y. Xue, D.M. York and P.A. Kollman *AMBER 2020, University of California, San Francisco*, 2020.

19. Roe, D. R.; Cheatham, T. E., Parallelization of CPPTRAJ enables large scale analysis of molecular dynamics trajectory data. *J. Comput. Chem.* **2018,** *39* (25), 2110-2117.

20. Roe, D. R.; Cheatham, T. E., PTRAJ and CPPTRAJ: Software for Processing and Analysis of Molecular Dynamics Trajectory Data. *Journal of Chemical Theory and*

*Computation* **2013,** *9* (7), 3084-3095.

21. D.A. Case, K. B., I.Y. Ben-Shalom, S.R. Brozell, D.S. Cerutti, T.E. Cheatham, III, V.W.D. Cruzeiro, T.A. Darden, R.E. Duke, G. Giambasu, M.K. Gilson, H. Gohlke, A.W. Goetz, R. Harris, S. Izadi, S.A. Izmailov, K. Kasavajhala, A. Kovalenko, R. Krasny, T. Kurtzman, T.S. Lee, S. LeGrand, P. Li, C. Lin, J. Liu, T. Luchko, R. Luo, V. Man, K.M. Merz, Y. Miao, O. Mikhailovskii, G. Monard, H. Nguyen, A. Onufriev, F.Pan, S. Pantano, R. Qi, D.R. Roe, A. Roitberg, C. Sagui, S. Schott-Verdugo, J. Shen, C. Simmerling, N.R.Skrynnikov, J. Smith, J. Swails, R.C. Walker, J. Wang, L. Wilson, R.M. Wolf, X. Wu, Y. Xiong, Y. Xue, D.M. York and P.A. Kollman, AMBER 2020, University of California, San Francisco. **2020**.

22. Srinivasan, J.; Cheatham, T. E.; Cieplak, P.; Kollman, P. A.; Case, D. A., Continuum solvent studies of the stability of DNA, RNA, and phosphoramidate - DNA helices. *J. Am. Chem. Soc.* **1998,** *120* (37), 9401-9409.

23. Lee, M. S.; Salsbury, F. R.; Olson, M. A., An efficient hybrid explicit/implicit solvent method for biomolecular simulations. *J. Comput. Chem.* **2004,** *25* (16), 1967-1978.

24. Naim, M.; Bhat, S.; Rankin, K. N.; Dennis, S.; Chowdhury, S. F.; Siddiqi, I.; Drabik, P.; Sulea, T.; Bayly, C. I.; Jakalian, A.; Purisima, E. O., Solvated interaction energy (SIE) for scoring protein-ligand binding affinities. 1. Exploring the parameter space. *J. Chem Inf. Model.* **2007,** *47* (1), 122-133.

25. Perdih, A.; Bren, U.; Solmajer, T., Binding free energy calculations of N-sulphonyl-glutamic acid inhibitors of MurD ligase. *J. Mol. Model.* **2009,** *15* (8), 983-996.

26. Bren, U.; Martinek, V.; Florian, J., Free energy simulations of uncatalyzed DNA replication fidelity: Structure and stability of T center dot G and dTTP center dot G terminal DNA mismatches flanked by a single dangling nucleotide. *J. Phys. Chem. B* **2006,** *110* (21), 10557-10566.

27. Aqvist, J.; Medina, C.; Samuelsson, J. E., A new method for predicting binding affinity in computer-aided drug design. *Protein Eng.* **1994,** *7* (3), 385-391.

28. Gilson, M. K.; Zhou, H. X., Calculation of protein-ligand binding affinities. *Annu. Rev. Biophys. Biomolec. Struct.* **2007,** *36*, 21-42.

29. Lee, F. S.; Chu, Z. T.; Bolger, M. B.; Warshel, A., Calculations of antibody-antigen interactions: microscopic and semi-microscopic evaluation of the free energies of binding of phosphorylcholine analogs to McPC603. *Protein Eng.* **1992,** *5* (3), 215-228.

30. Shi, M.; Wang, L.; Li, P.; Liu, J.; Chen, L.; Xu, D., Dasatinib-SIK2 binding elucidated by homology modeling, molecular docking, and dynamics simulations. *ACS Omega* **2021,** *6* (16), 11025-11038.

31. King, E.; Aitchison, E.; Li, H.; Luo, R., Recent Developments in Free Energy Calculations for Drug Discovery. *Front. Mol. Biosci.* **2021,** *8*, 24.

32. Cheng, P.; Li, J. J.; Wang, J.; Zhang, X. Y.; Zhai, H. L., Investigations of FAK inhibitors: a combination of 3D-QSAR, docking, and molecular dynamics simulations studies. *J. Biomol. Struct. Dyn.* **2018,** *36* (6), 1529-1549.

33. Shirvani, P.; Fassihi, A., In silico design of novel FAK inhibitors using integrated molecular docking, 3D-QSAR and molecular dynamics simulation studies. *J. Biomol. Struct. Dyn.* **2022,** *40* (13), 5965-5982.

34. Shi, M.; Chen, T.; Wei, S.; Zhao, C.; Zhang, X.; Li, X.; Tang, X.; Liu, Y.; Yang, Z.; Chen, L., Molecular Docking, Molecular Dynamics Simulations, and Free Energy Calculation Insights into the Binding Mechanism between VS-4718 and Focal Adhesion Kinase. *ACS Omega* **2022,** *7* (36), 32442-32456.

35. Weiser, J.; Shenkin, P. S.; Still, W. C., Approximate atomic surfaces from linear combinations of pairwise overlaps (LCPO). *J. Comput. Chem.* **1999,** *20* (2), 217-230.

36. Still, W. C.; Tempczyk, A.; Hawley, R. C.; Hendrickson, T., Semianalytical treatment of solvation for molecular mechanics and dynamics. *J. Am. Chem. Soc.* **1990,** *112* (16), 6127-6129.

37. Srinivasan, J.; Trevathan, M. W.; Beroza, P.; Case, D. A., Application of a pairwise generalized Born model to proteins and nucleic acids: inclusion of salt effects. *Theor. Chem. Acc.* **1999,** *101* (6), 426-434.

38. Ma, T.; Tian, X.; Zhang, B. D.; Li, M. Q.; Wang, Y.; Yang, C. Y.; Wu, J. F.; Wei, X. Y.; Qu, Q.; Yu, Y. X.; Long, S. T.; Feng, J. W.; Li, C.; Zhang, C. X.; Xie, C. C.; Wu, Y. Y.; Xu, Z. N.; Chen, J. J.; Yu, Y.; Huang, X.; He, Y.; Yao, L. M.; Zhang, L.; Zhu, M. X.; Wang, W.; Wang, Z. C.; Zhang, M. L.; Bao, Y. Q.; Jia, W. P.; Lin, S. Y.; Ye, Z. Y.; Piao, H. L.; Deng, X. M.; Zhang, C. S.; Lin, S. C., Low-dose metformin targets the lysosomal AMPK pathway through PEN2. *Nature* **2022,** *603* (7899), 159-+.

39. Saikia, S.; Bordoloi, M., Molecular docking: Challenges, advances and its use in drug discovery perspective. *Curr. Drug Targets* **2019,** *20* (5), 501-521.

40. Ferreira, L. G.; dos Santos, R. N.; Oliva, G.; Andricopulo, A. D., Molecular docking and structure-based drug design strategies. *Molecules* **2015,** *20* (7), 13384-13421.

41. Sanner, M. F., Python: A programming language for software integration and development. *J. Mol. Graph.* **1999,** *17* (1), 57-61.

42. Stewart, J. J. P., Optimization of parameters for semiempirical methods. III Extension of PM3 to Be, Mg, Zn, Ga, Ge, As, Se, Cd, In, Sn, Sb, Te, Hg, Tl, Pb, and Bi. *J. Comput. Chem.* **1991,** *12* (3), 320-341.

43. Stewart, J. J. P., Optimization of parameters for semiempirical methods I. Method. *J. Comput. Chem.* **1989,** *10* (2), 209-220.

44. Stewart, J. J. P., Optimization of parameters for semiempirical methods II. Applications *J. Comput. Chem.* **1989,** *10* (2), 221-264.

45. Stewart, J. J. P., MOPAC: A semiempirical molecular orbital program. *J. Comput.-Aided Mol. Des.* **1990,** *4* (1), 1-45.

46. Morris, G. M.; Huey, R.; Lindstrom, W.; Sanner, M. F.; Belew, R. K.; Goodsell, D. S.; Olson, A. J., AutoDock4 and AutoDockTools4: Automated docking with selective receptor flexibility. *J. Comput. Chem.* **2009,** *30* (16), 2785-2791.

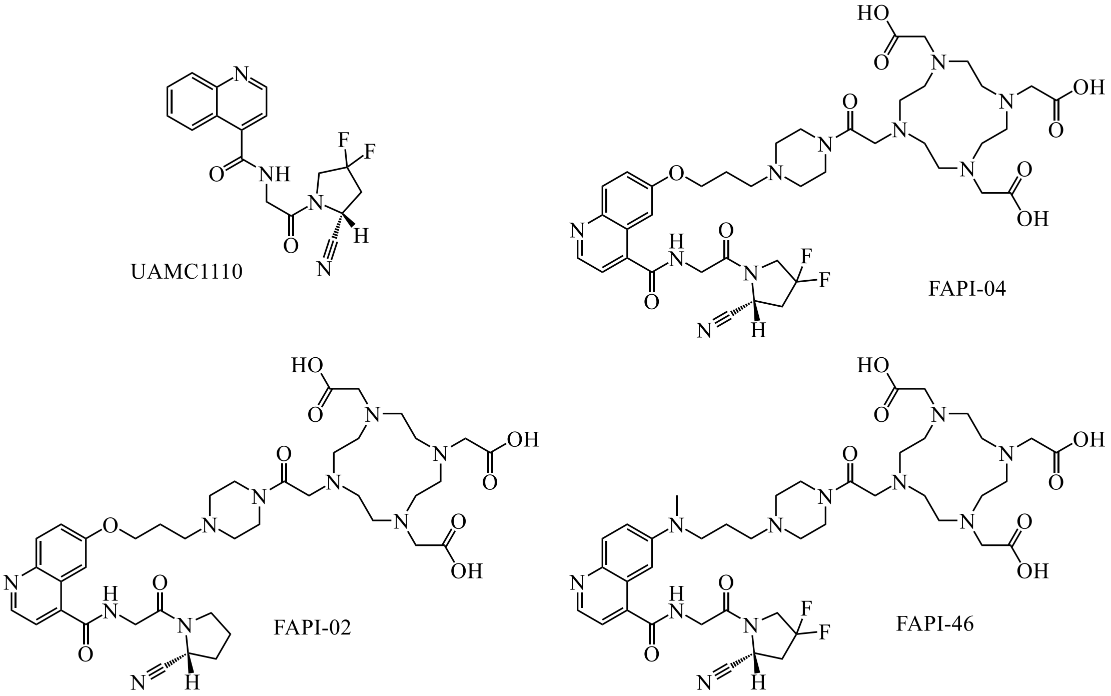


**Figure S1. Structures for FAP inhibitors**

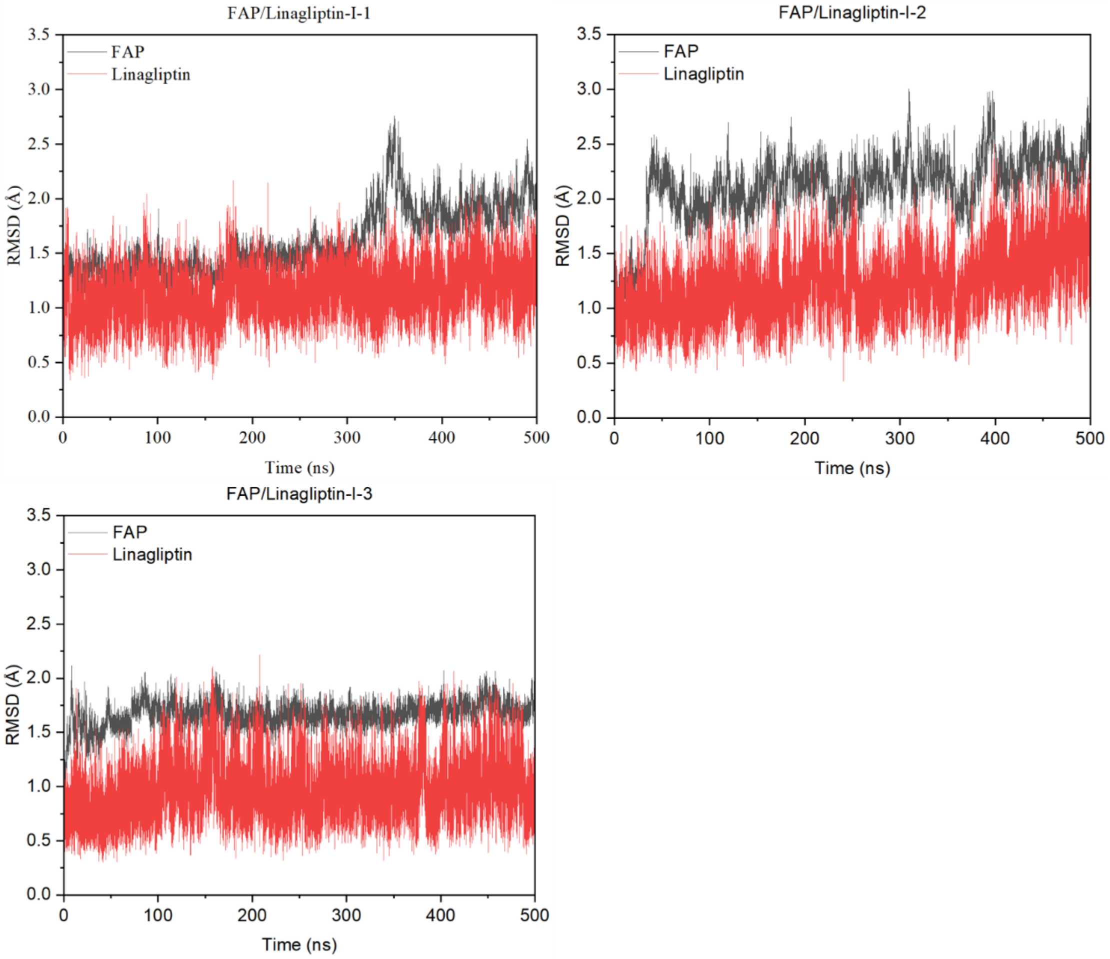


**Figure S2. Root mean square deviation (RMSD) value of heavy atoms of backbone for human FAP and no-hydrogen atoms of linagliptin along 500 ns MD simulation for three linagliptin/FAP-I complex systems**

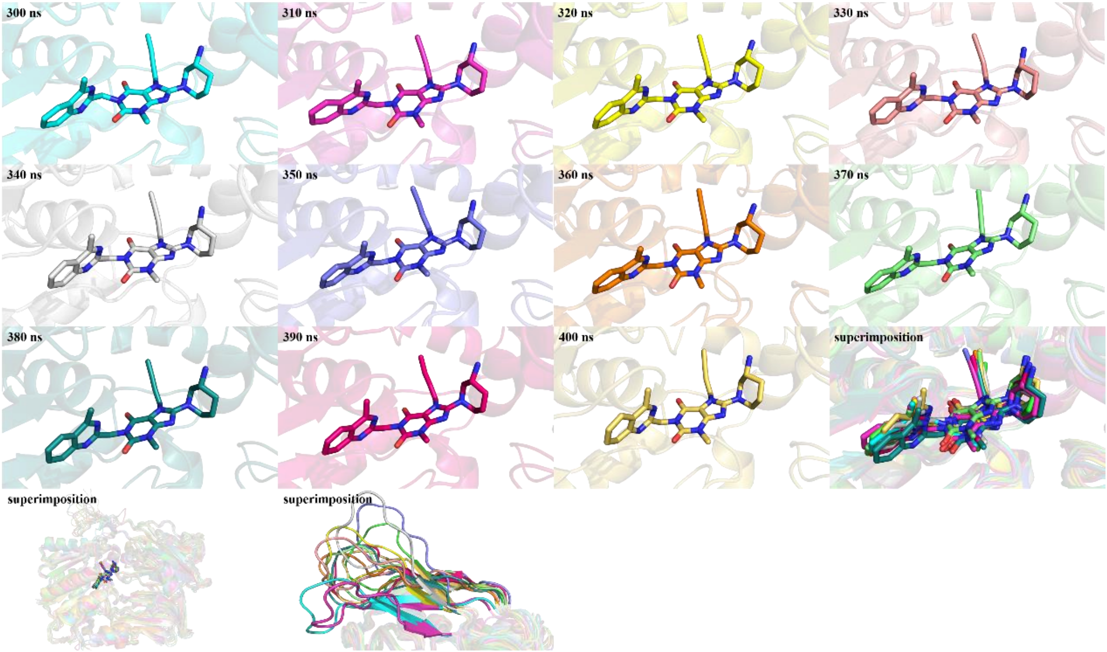


**Figure S3. Snapshots of the linagliptin/FAP-I-1 along the dynamic simulation time for 400-500 ns**

For clarity, the water molecules have been removed. The inhibitor linagliptin is plotted using stick style, while cartoon style for human FAP.

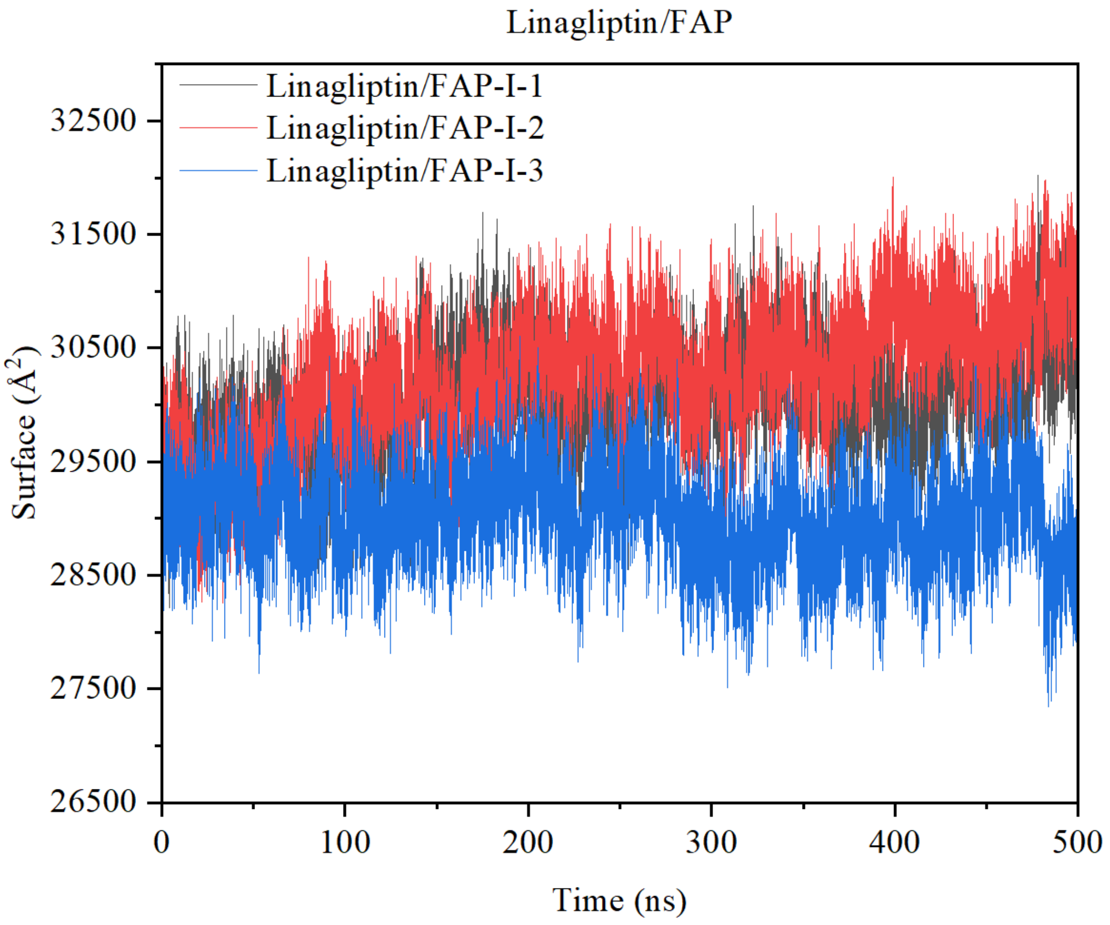


**Figure S4. Surface area for linagliptin binding with FAP**

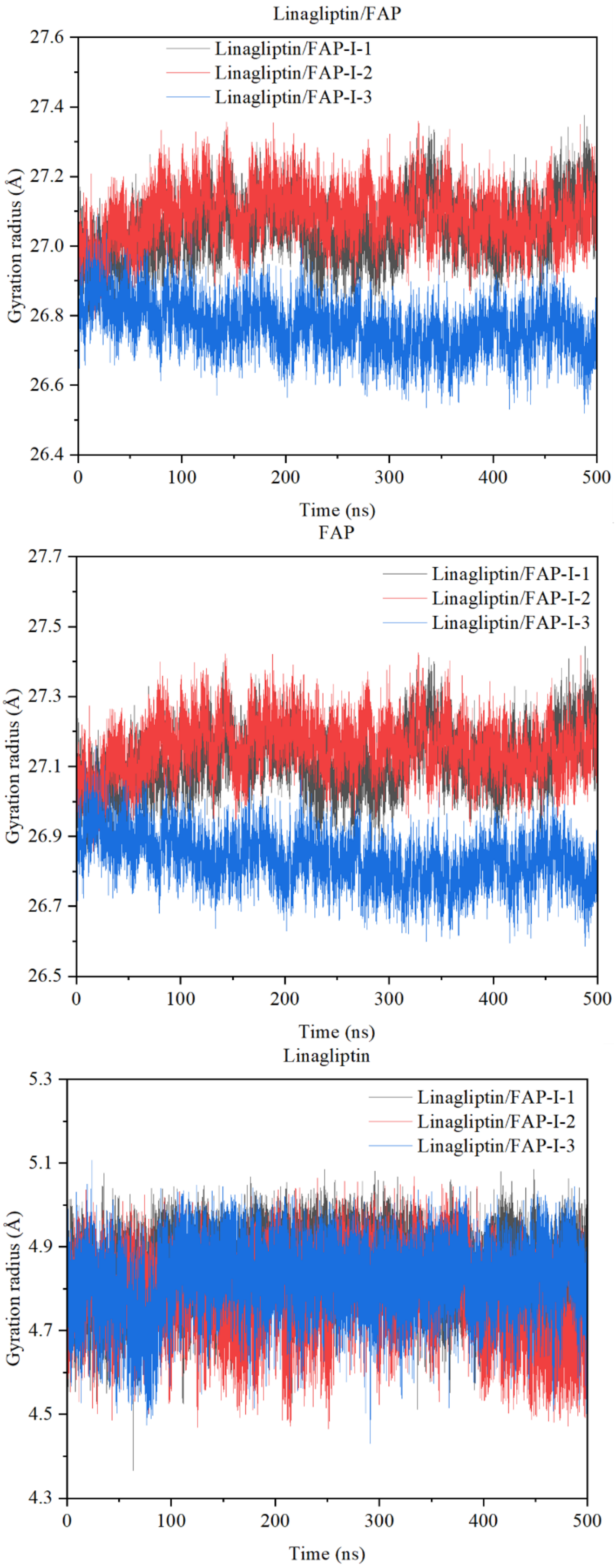


**Figure S5. Gyration radius for linagliptin binding with FAP**

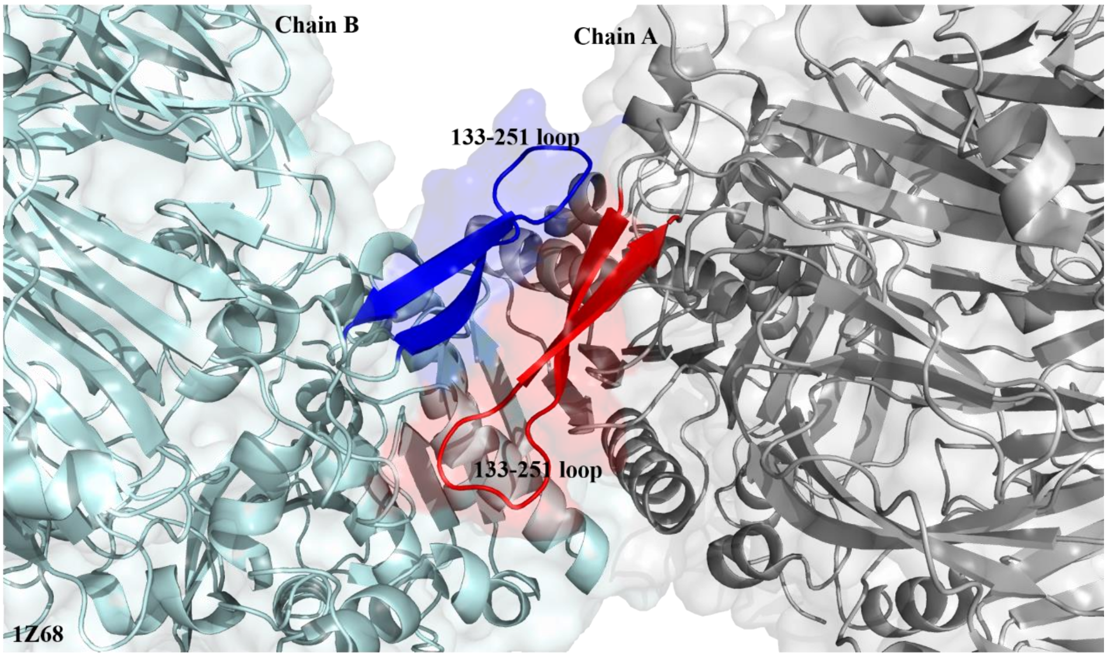


**Figure S6. Dimer structure of human FAP**

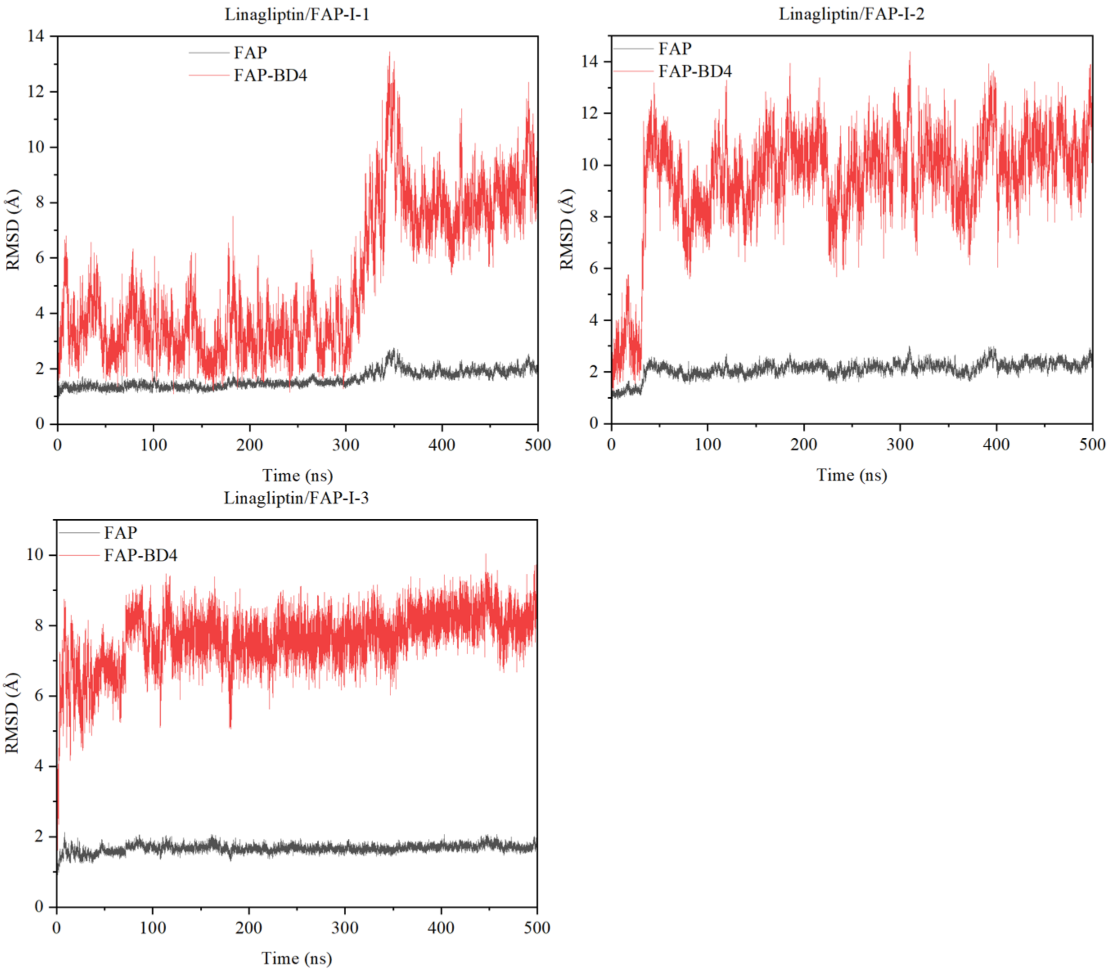


**Figure S7. Root mean square deviation (RMSD) value of heavy atoms of backbone for human FAP and BD4 domain of FAP along 500 ns MD simulation for three linagliptin/FAP-I complex systems**

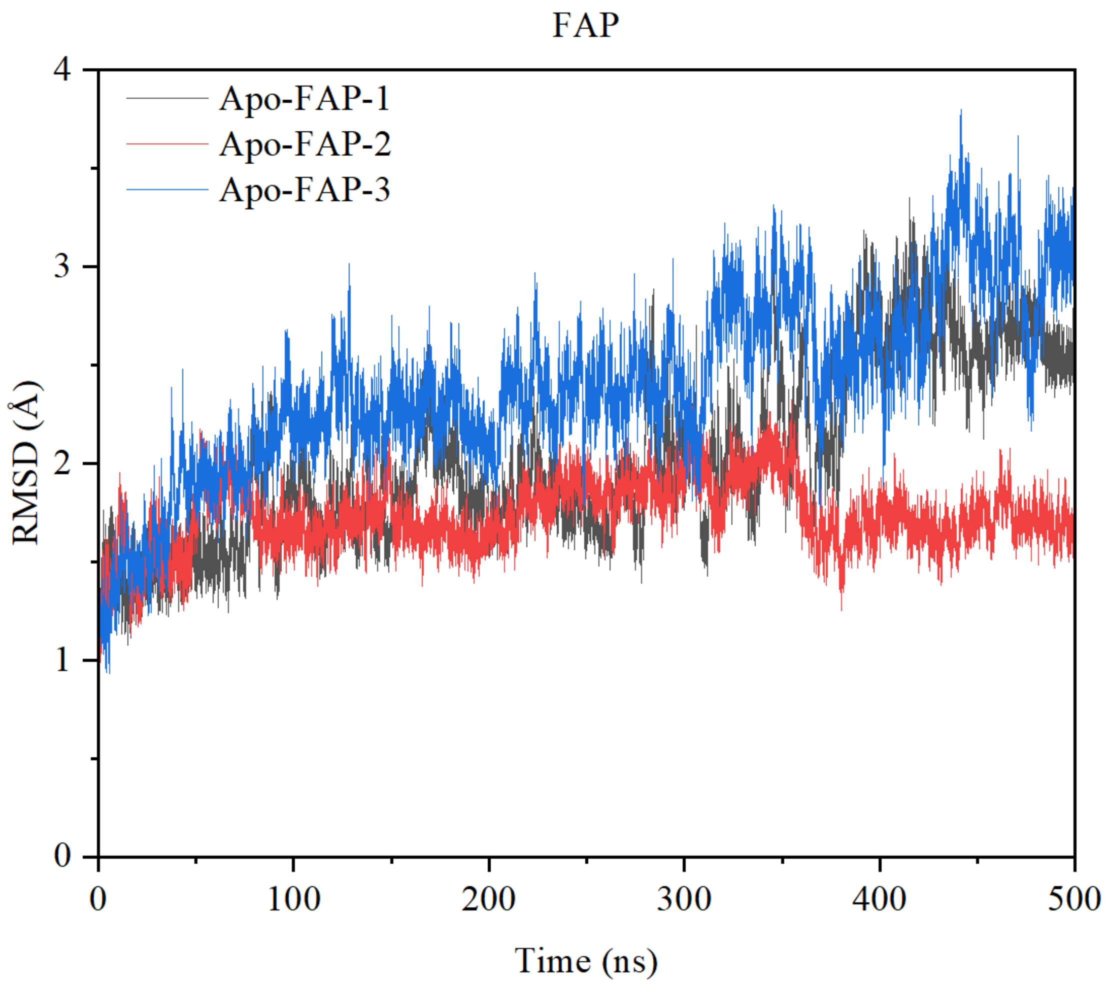


**Figure S8. Root mean square deviation (RMSD) value of heavy atoms of backbone for human FAP along 500 ns MD simulation for apo-FAP systems**

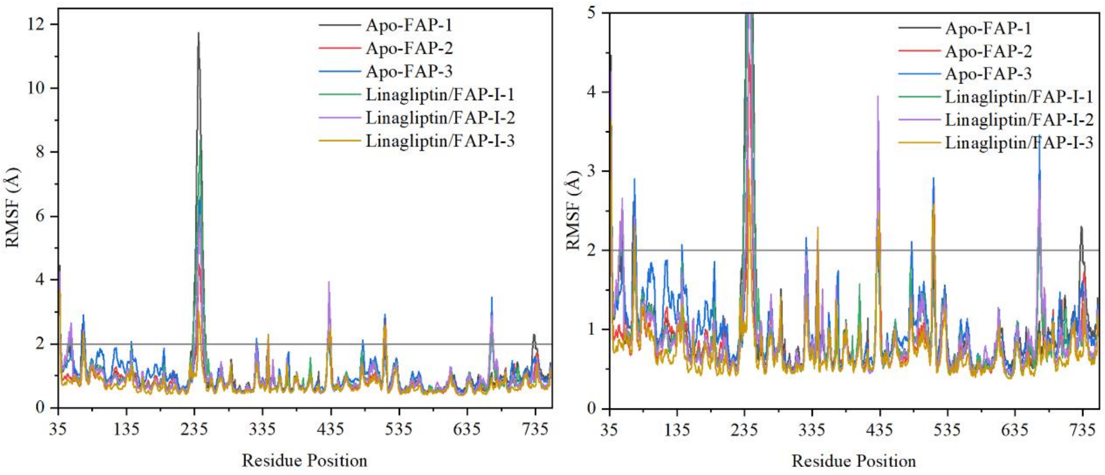


**Figure S9. RMSF variations for Cα atom of human FAP for linagliptin/FAP-I and Apo-FAP systems from the 500 ns MD simulation**

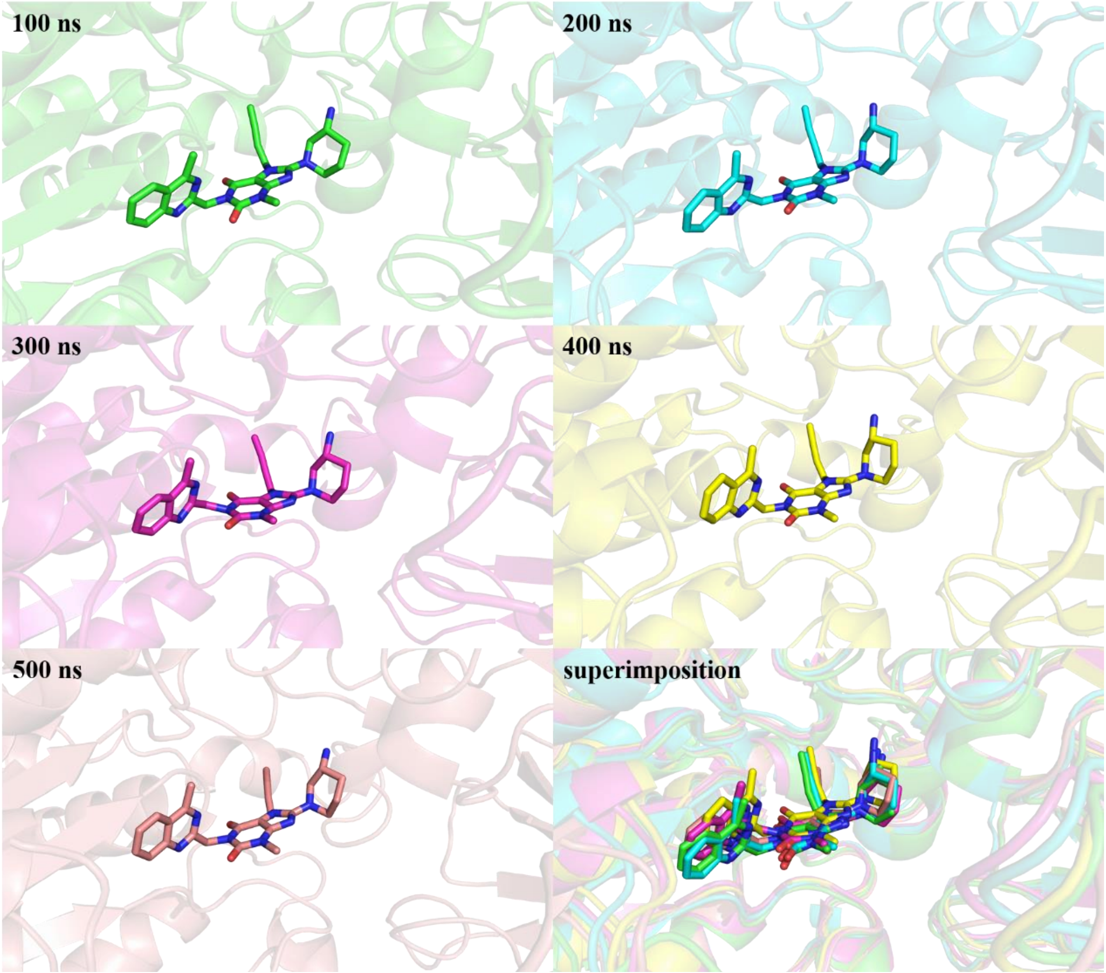


**Figure S10. Snapshots of the linagliptin/FAP-I-1 along the dynamic simulation time for 100, 200, 300, 400, and 500 ns**

For clarity, the water molecules have been removed. The inhibitor linagliptin is plotted using stick style, while cartoon style for human FAP.

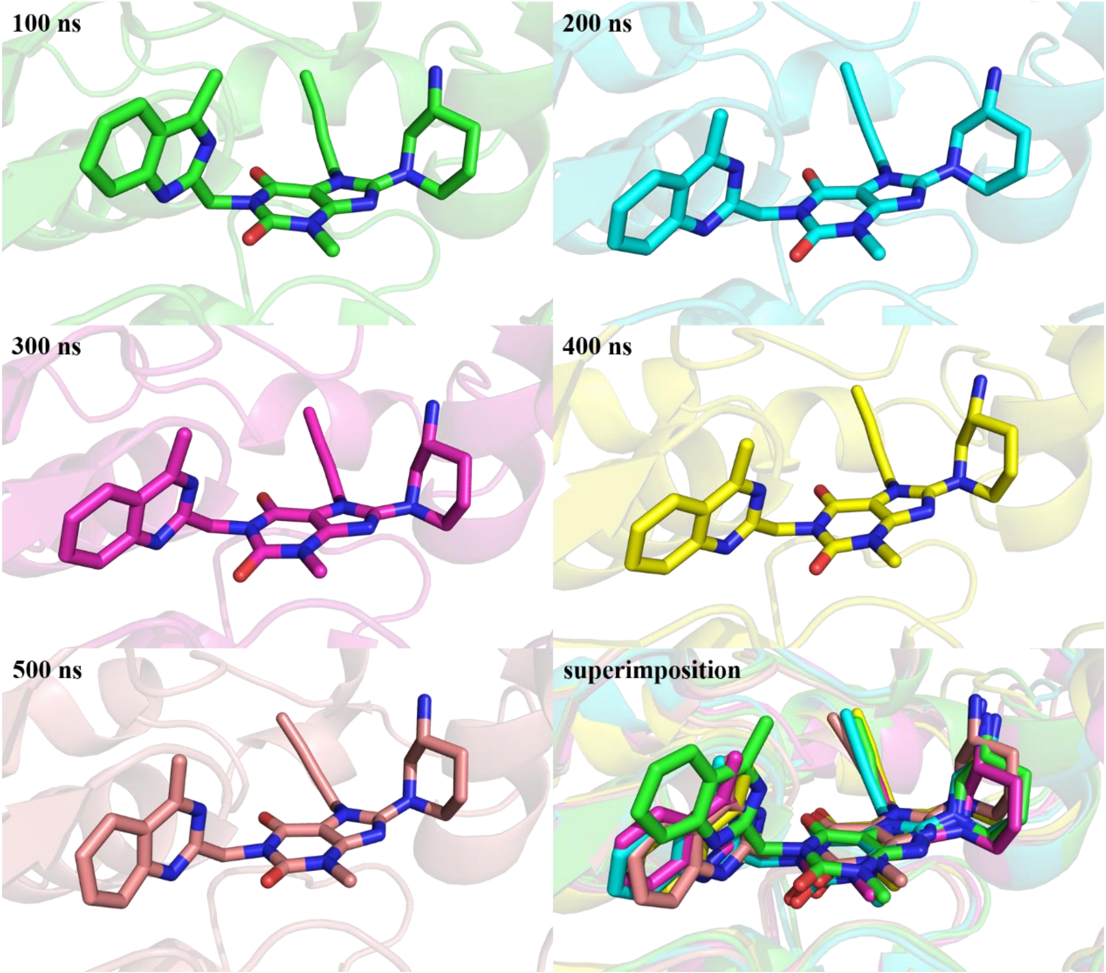


**Figure S11. Snapshots of the linagliptin/FAP-I-2 along the dynamic simulation time for 100, 200, 300, 400, and 500 ns**

For clarity, the water molecules have been removed. The inhibitor linagliptin is plotted using stick style, while cartoon style for human FAP.

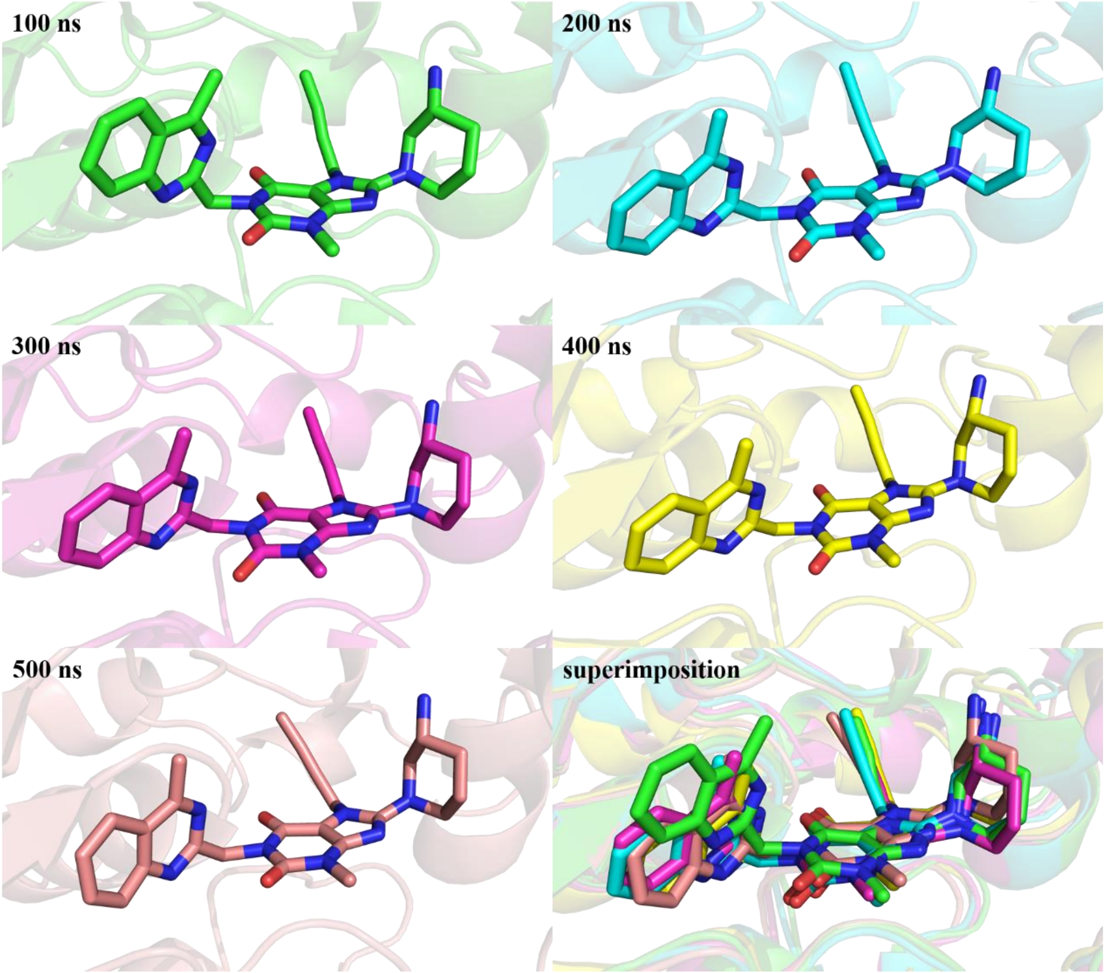


**Figure S12. Snapshots of the linagliptin/FAP-I-3 along the dynamic simulation time for 100, 200, 300, 400, and 500 ns**

For clarity, the water molecules have been removed. The inhibitor linagliptin is plotted using stick style, while cartoon style for human FAP.

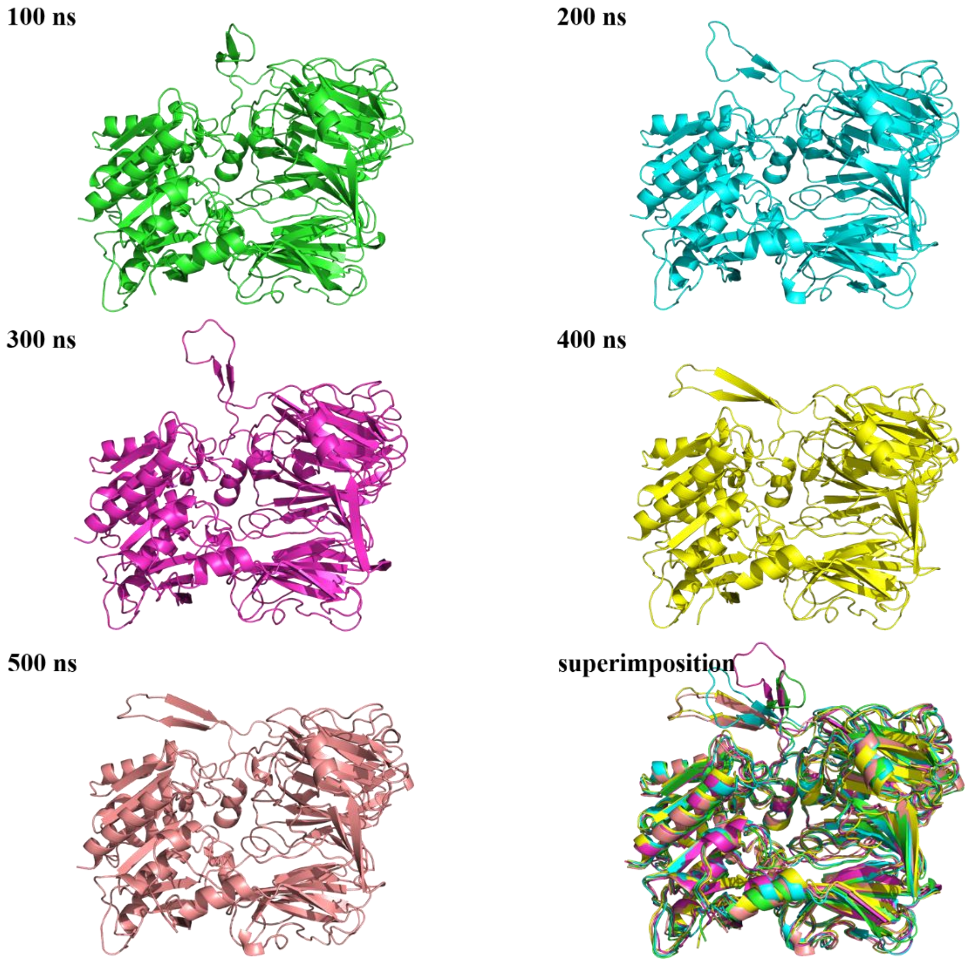


**Figure S13. Snapshots of the apo-FAP-1 along the dynamic simulation time for 100, 200, 300, 400, and 500 ns**

For clarity, the water molecules have been removed. The human FAP is plotted using cartoon style.

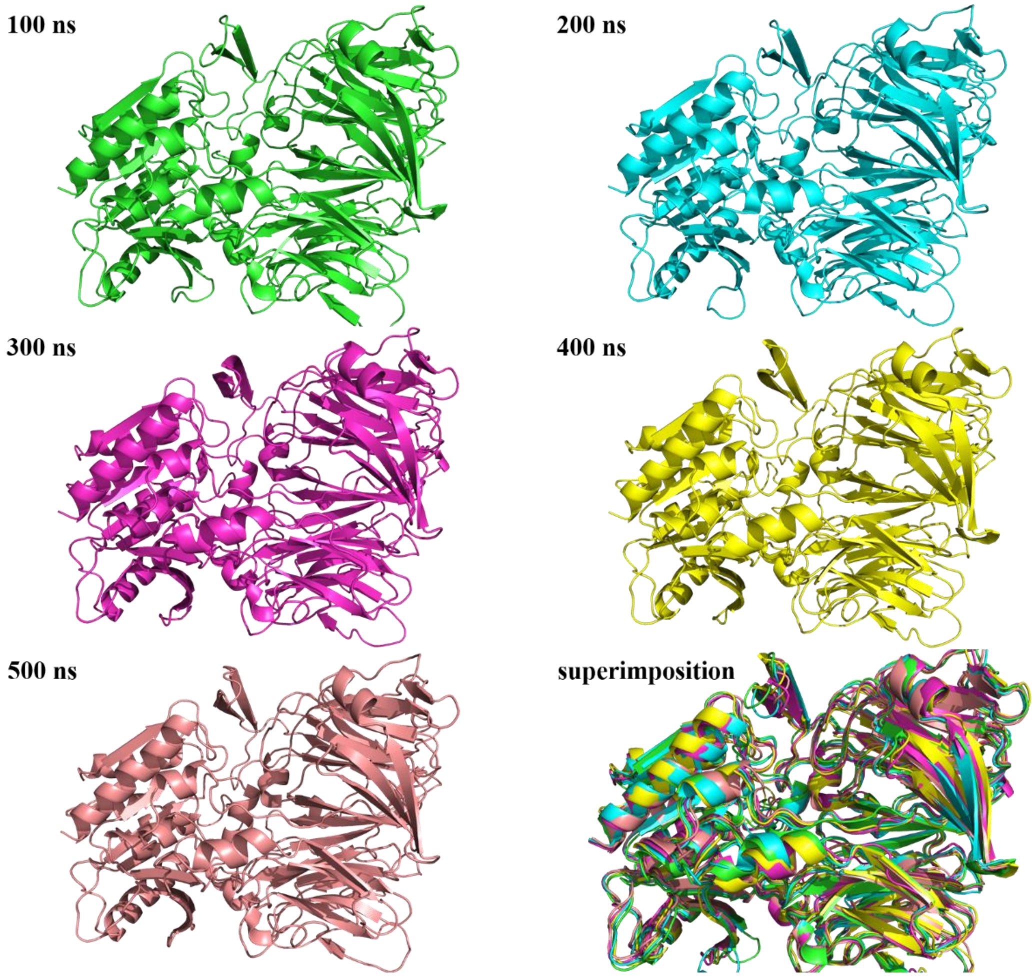


**Figure S14. Snapshots of the apo-FAP-2 along the dynamic simulation time for 100, 200, 300, 400, and 500 ns**

For clarity, the water molecules have been removed. The human FAP is plotted using cartoon style.

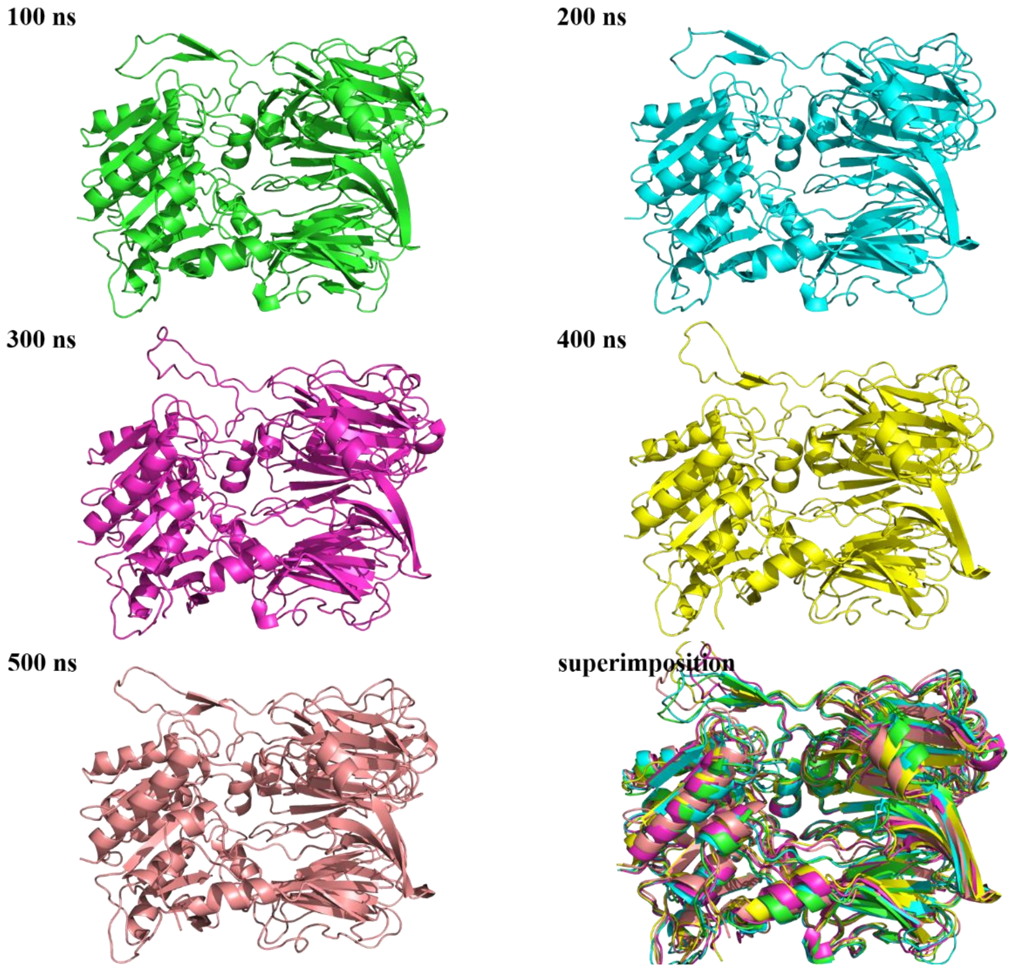


**Figure S15. Snapshots of the apo-FAP-3 along the dynamic simulation time for 100, 200, 300, 400, and 500 ns**

For clarity, the water molecules have been removed. The human FAP is plotted using cartoon style.

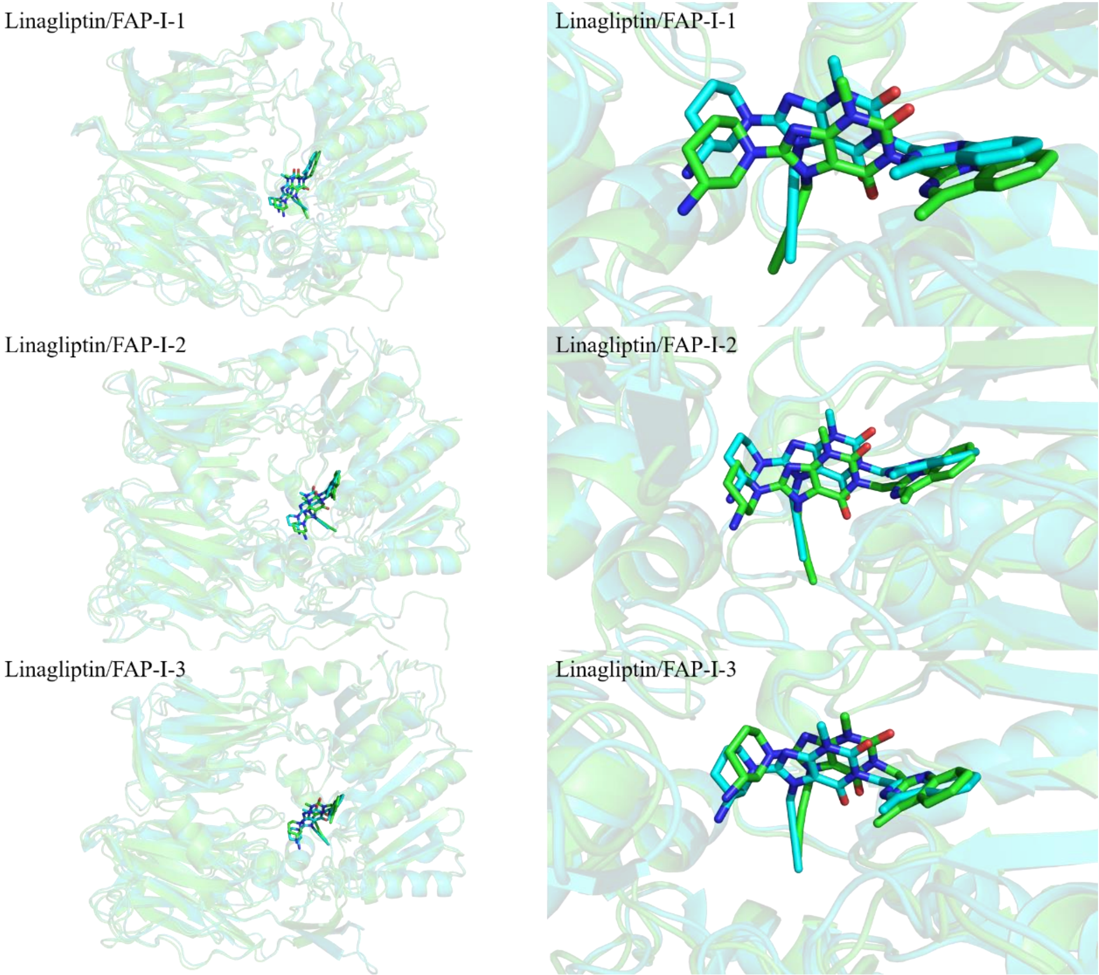


**Figure S16. Frames of linagliptin/FAP-I complex systems for initial and 500$^{th}$ ns**
Grey indicates the initial frame and green indicates the frame from 500th ns MD simulation.

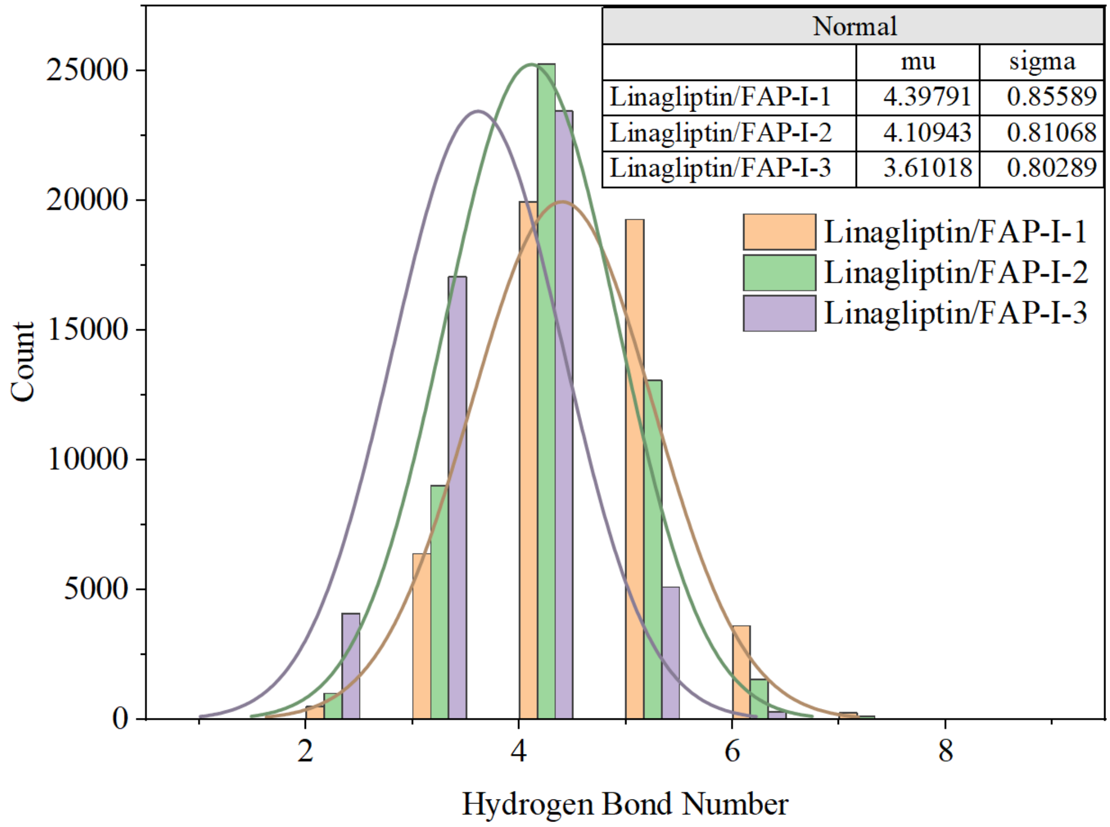


**Figure S17. Statistical hydrogen bond number profile along the 500-ns MD simulation for linagliptin/FAP-I**

Hydrogen bond is defined as the distance between the acceptor and donor atoms < 3.5 Å, with an internal angle between the H-acceptor and H-donor > 120º.

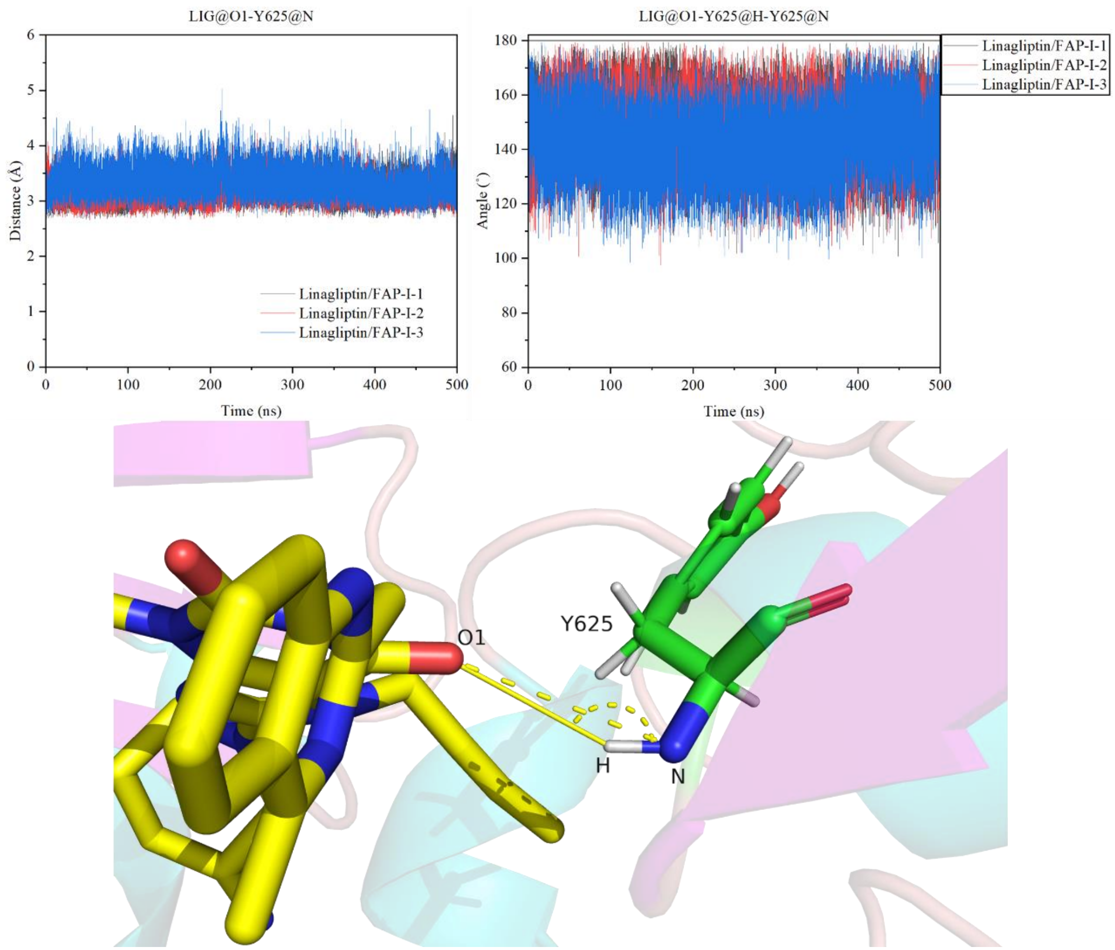


**Figure S18. Hydrogen bond parameters for hydrogen bond between LIG@O1 and Y625@N for linagliptin/FAP-I complex system**

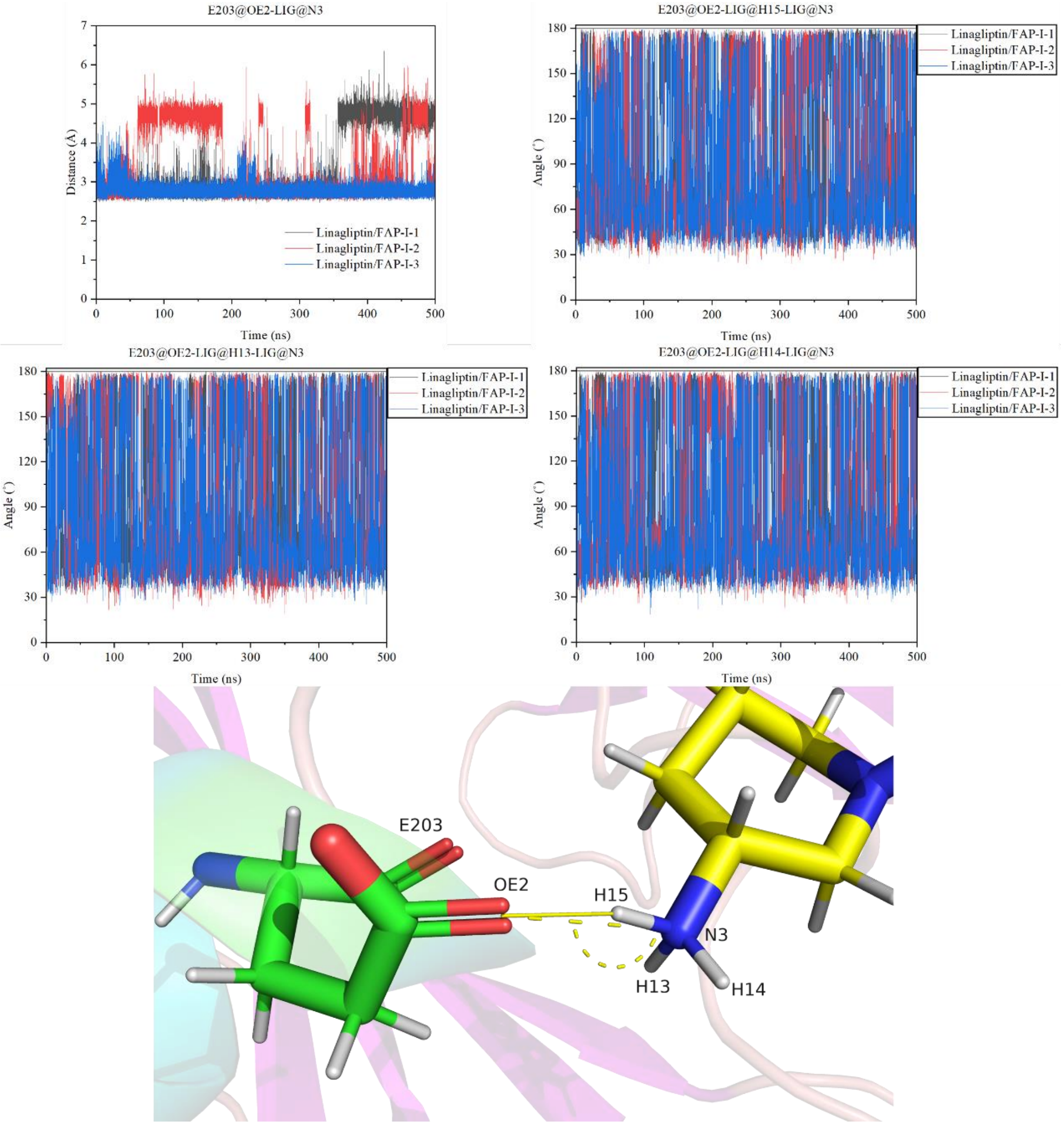


**Figure S19. Hydrogen bond parameters for hydrogen bond between E203@OE2 and LIG@N3 for linagliptin/FAP-I complex system**

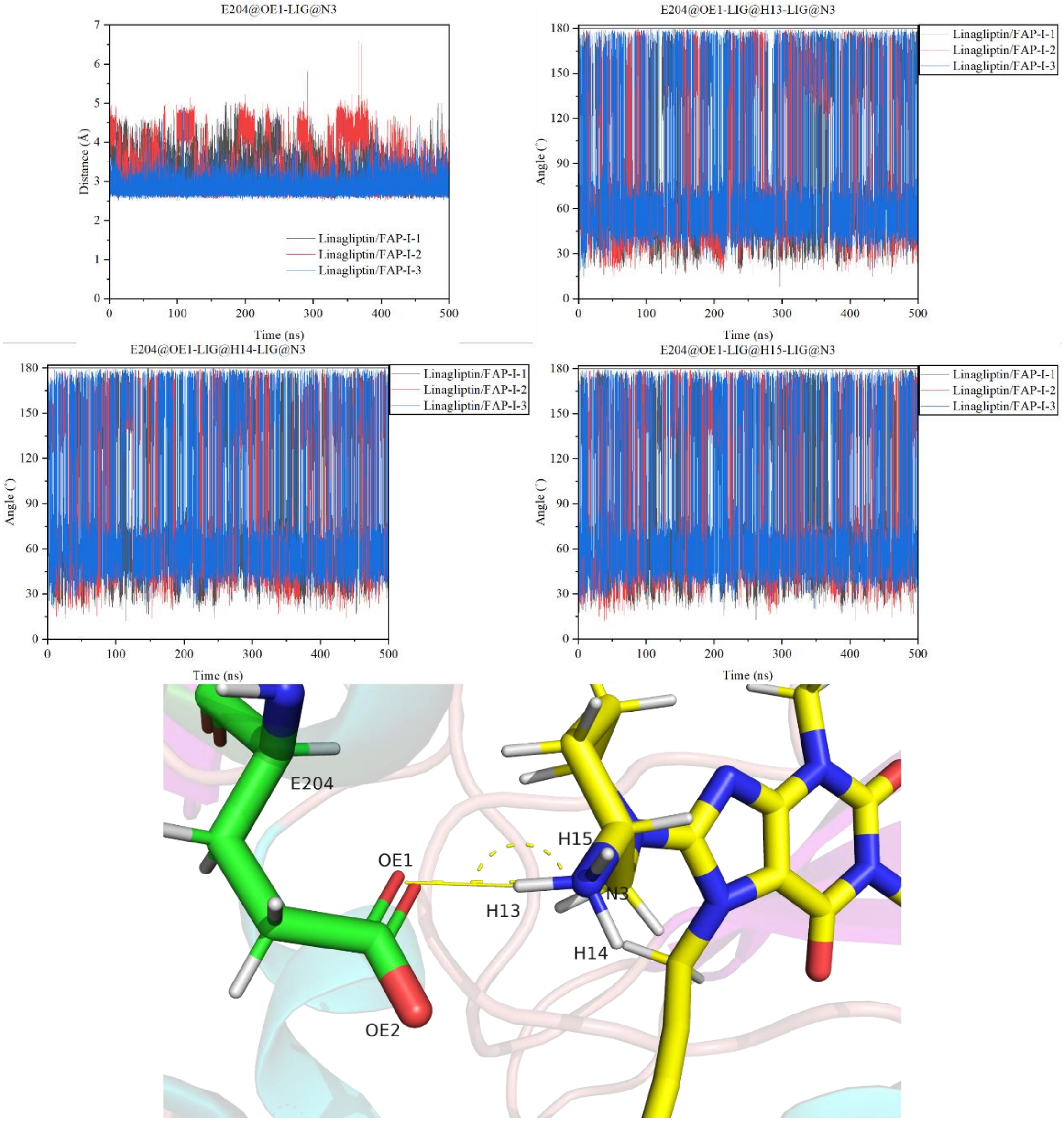


**Figure S20. Hydrogen bond parameters for hydrogen bond between E204@OE1 and LIG@N3 for linagliptin/FAP-I complex system**

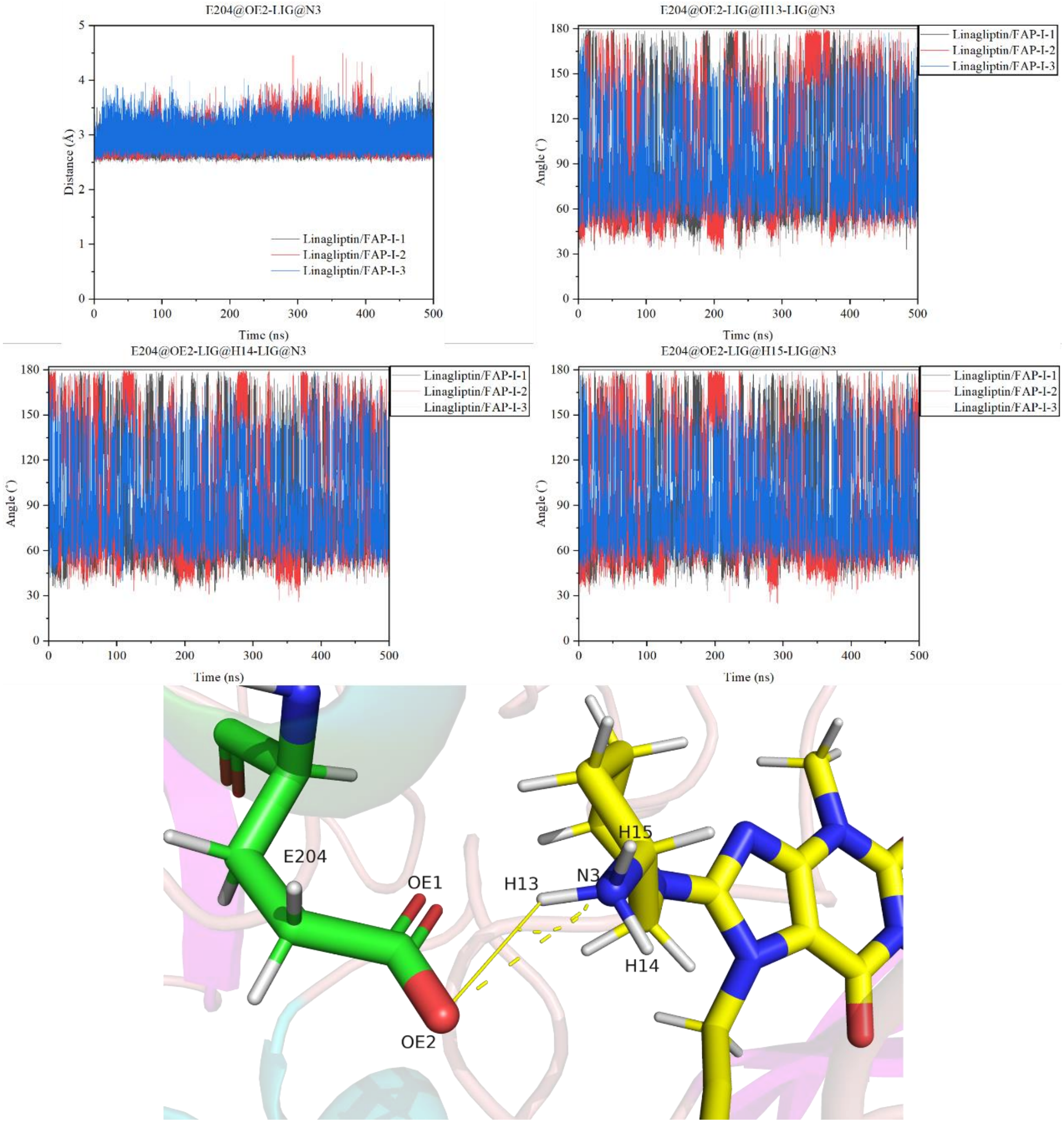


**Figure S21. Hydrogen bond parameters for hydrogen bond between E204@OE2 and LIG@N3 for linagliptin/FAP-I complex system**

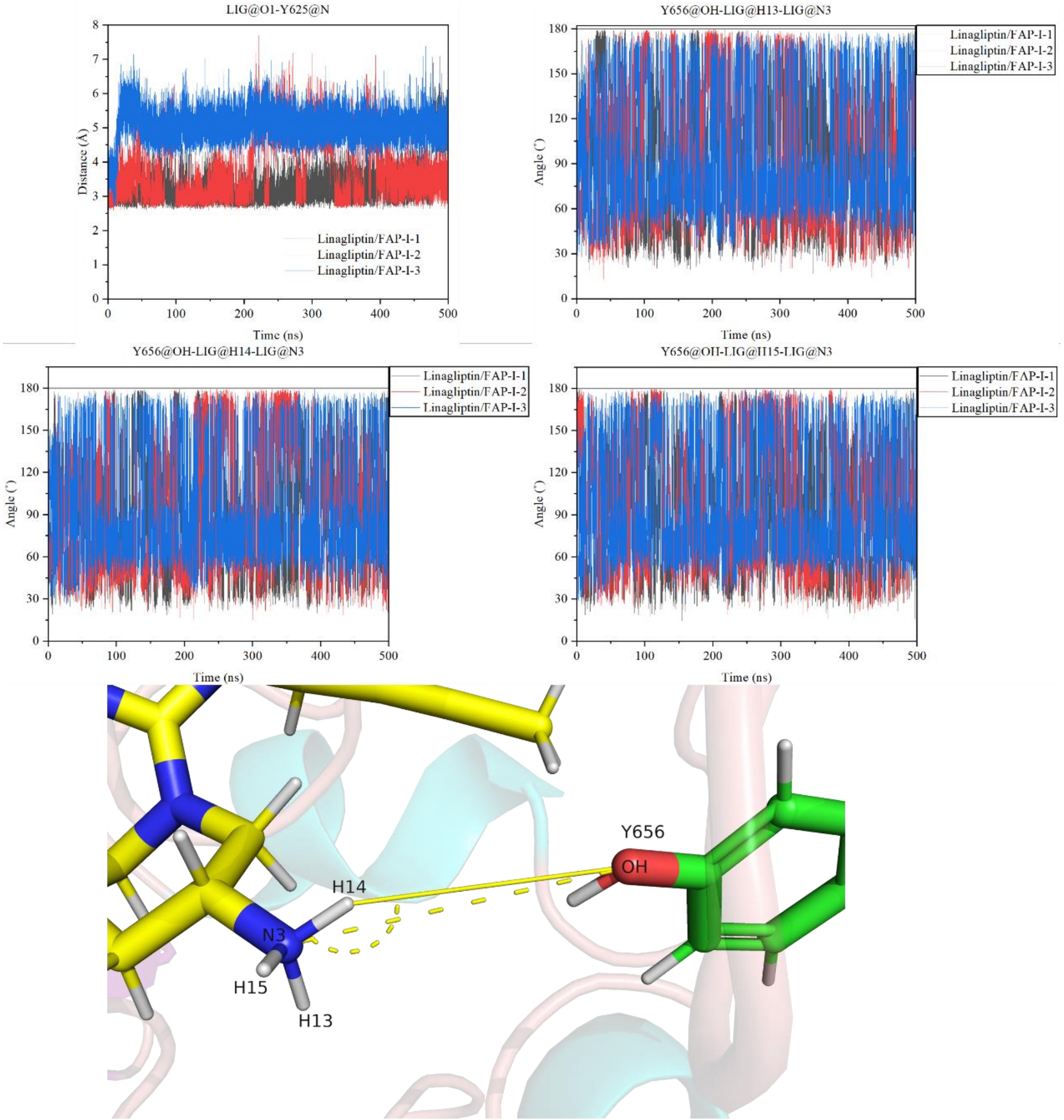


**Figure S22. Hydrogen bond parameters for hydrogen bond between Y656@OH and LIG@N3 for linagliptin/FAP-I complex system**

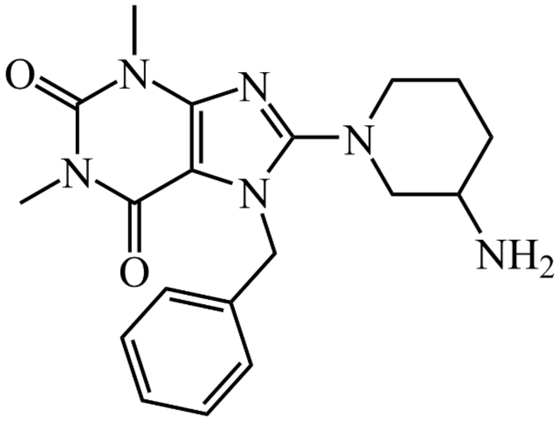

6: $IC_{50}$ = 82 nM (DPP-4)

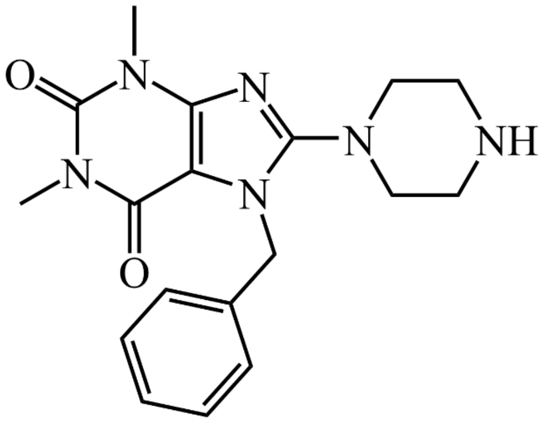

5a: $IC_{50}$ = 2800 nM (DPP-4)

**Figure S23. Activity and structure for piperazine and 3-aminopiperidine groups at C8 of xanthine moiety**

The data obtained from reference (*J. Med. Chem.* **2007,** *50* (26), 6450-6453).

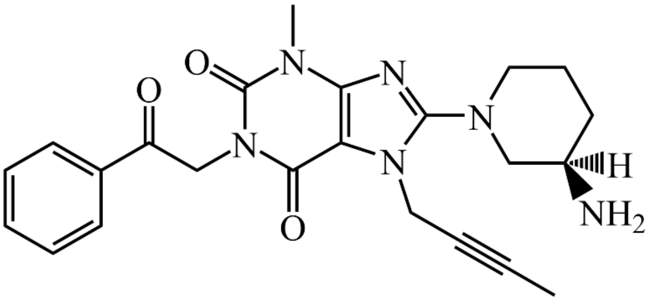


R-6bf: $IC_{50}$ = 4 nM (DPP-4)

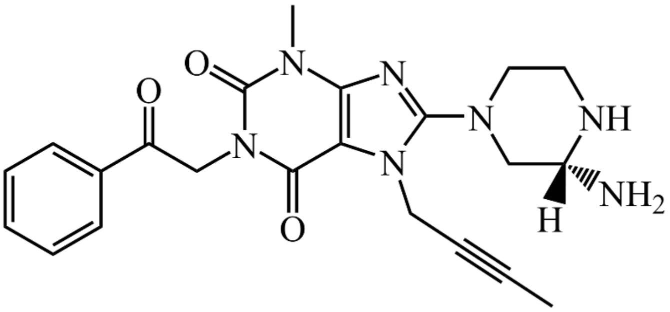


S-6bf: $IC_{50}$ = 9 nM (DPP-4)

**Figure S24. Activity and structure for the R and S conformations for the 3-aminopiperidine groups at C8 of the xanthine moiety**

The data obtained from reference (*J. Med. Chem.* **2007,** *50* (26), 6450-6453).

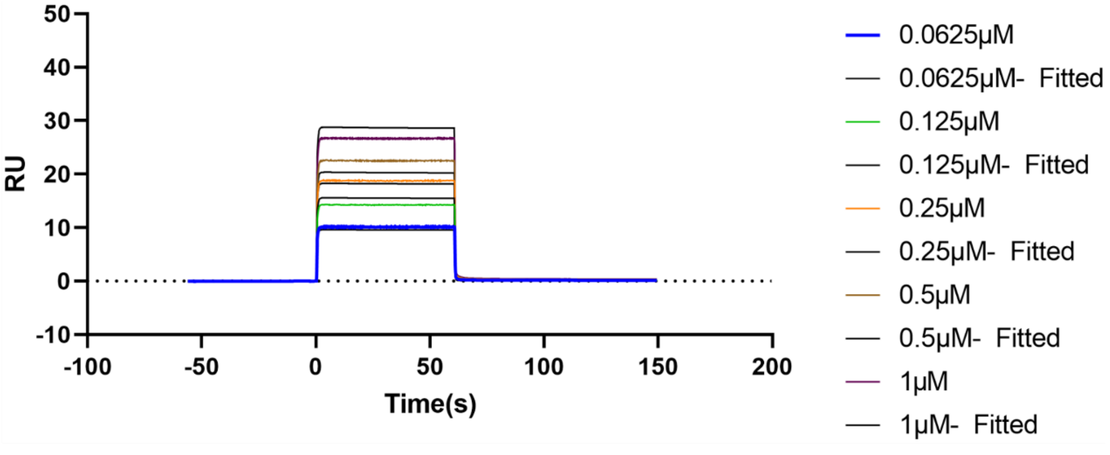


| $K_D$ | $k_{on}$ | $k_{off}$ |
|---|---|---|
| M | 1/Ms | 1/s |
| 2.43E-07 | 3665151.541 | 0.890631824 |

**Figure S25. Linagliptin binding with human FAP from surface plasmon resonance experiments**

Multicycle kinetic is applied for linagliptin binding with FAP in surface plasmon resonance experiment results.

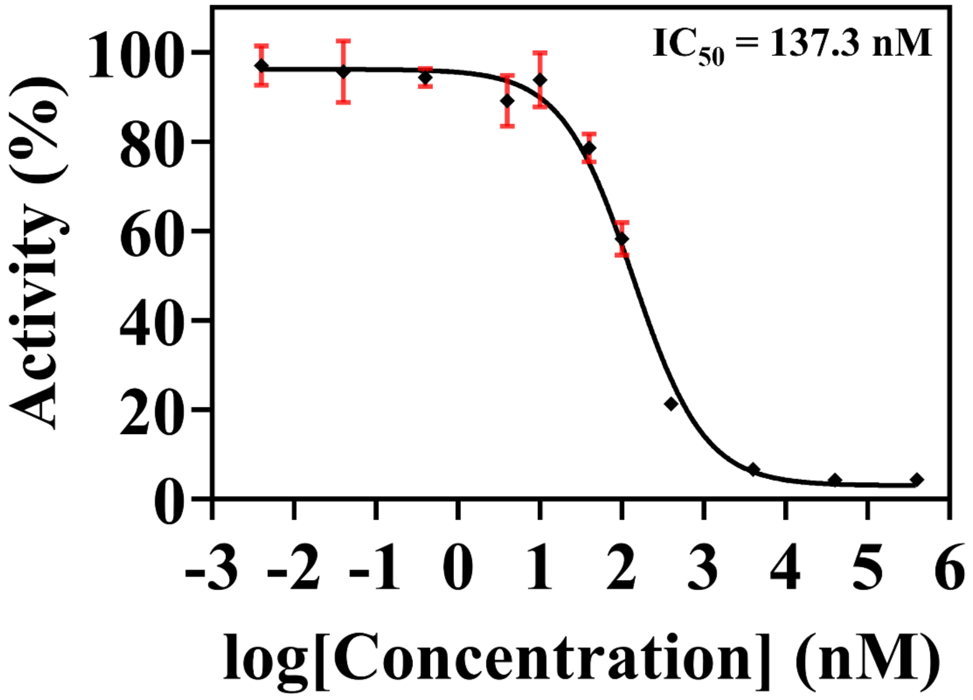


**Figure S26. Inhibition activity for linagliptin with FAP**

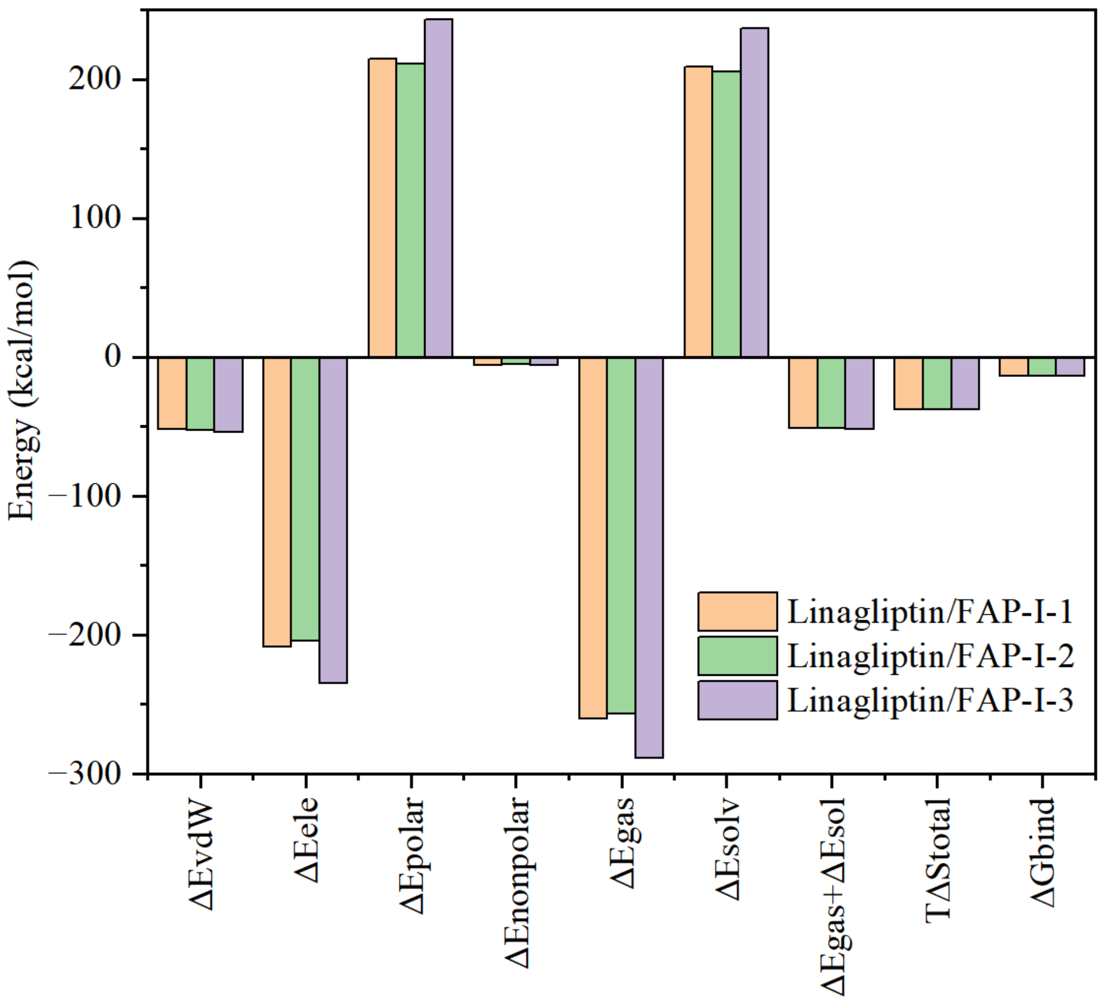


**Figure S27. Energy for linagliptin binding with FAP for linagliptin/FAP-I systems**

The binding free energies ($\Delta G_{bind}$) for linagliptin/FAP complex and decomposition to electrostatic interaction ($\Delta E_{ele}$), van der Walls interaction ($\Delta E_{vdW}$), polar solvation free energies ($\Delta E_{polar}$), nonpolar solvation free energies ($\Delta E_{nonpolar}$), and entropy ($\Delta TS_{total}$). Energy values are presented in kcal/mol. Uncertainties were calculated as the root mean square error for all frames extracted from the trajectories.

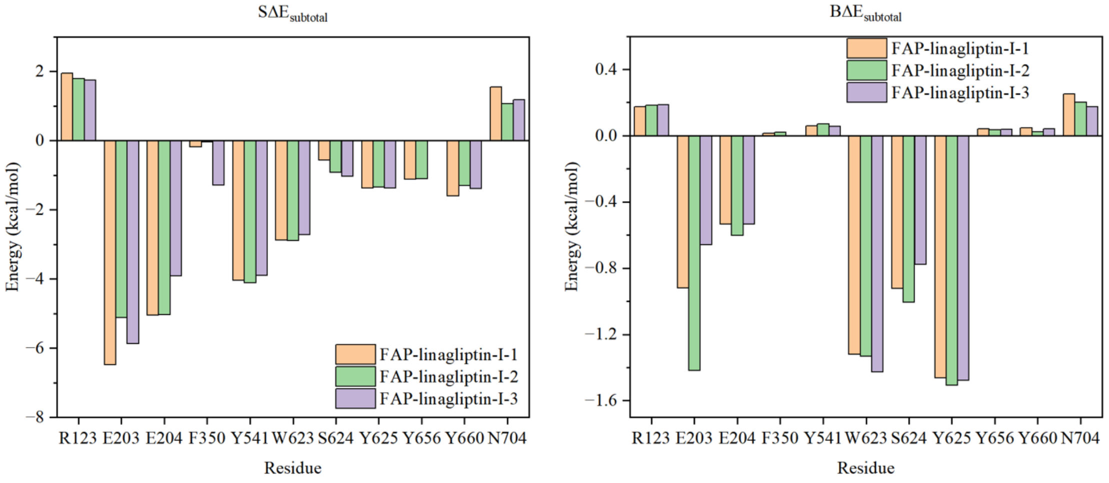


**Figure S28. Energy decomposition with backbone and sidechain for linagliptin/FAP-I systems**

The binding free energies ($\Delta G_{bind}$) for linagliptin/FAP complex and decomposition to backbone interaction ($B\Delta E_{subtotal}$), and sidechain interaction ($S\Delta E_{subtotal}$). Energy values are presented in kcal/mol.

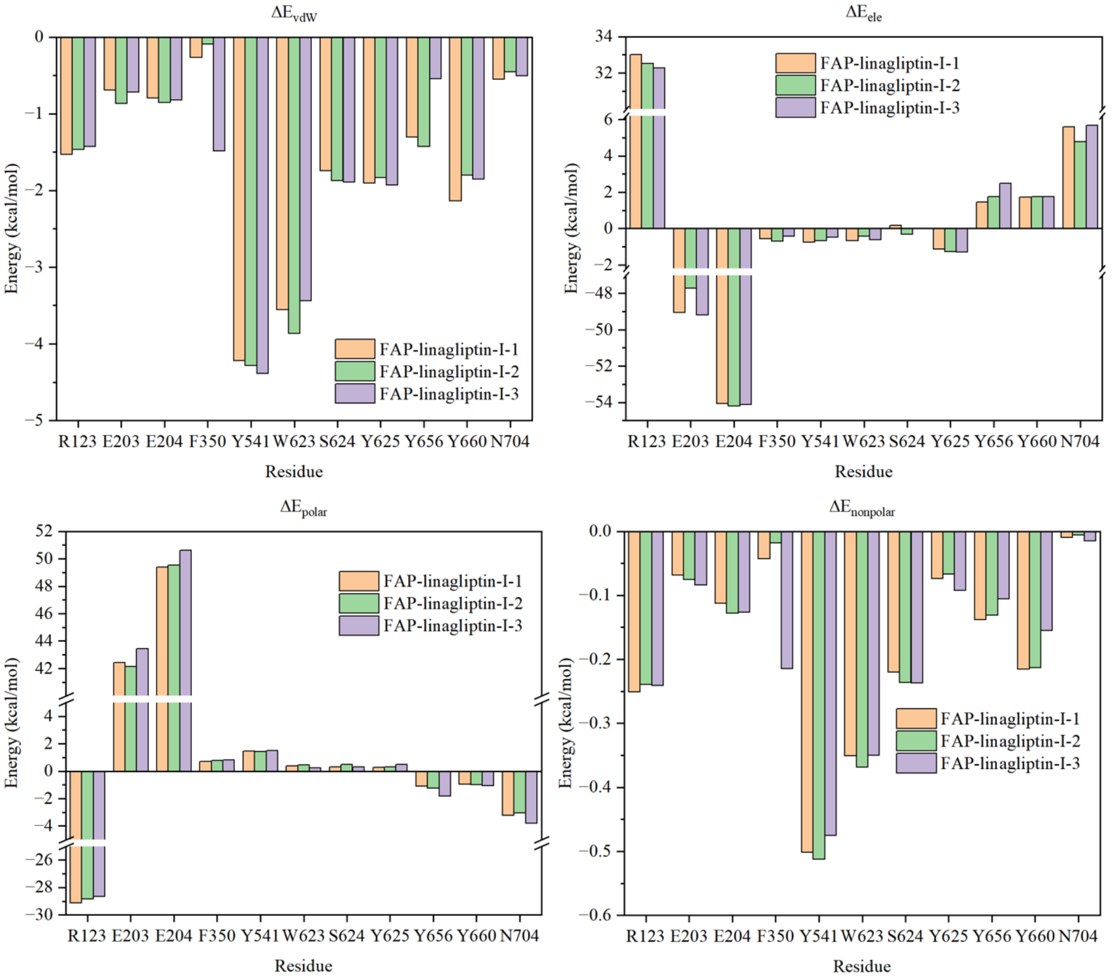


**Figure S29. Energy decomposition for linagliptin/FAP-I systems**

The binding free energies ($\Delta G_{bind}$) for linagliptin/FAP complex and decomposition to electrostatic interaction ($\Delta E_{ele}$), van der Walls interaction ($\Delta E_{vdW}$), polar solvation free energies ($\Delta E_{polar}$), and nonpolar solvation free energies ($\Delta E_{nonpolar}$). Energy values are presented in kcal/mol.

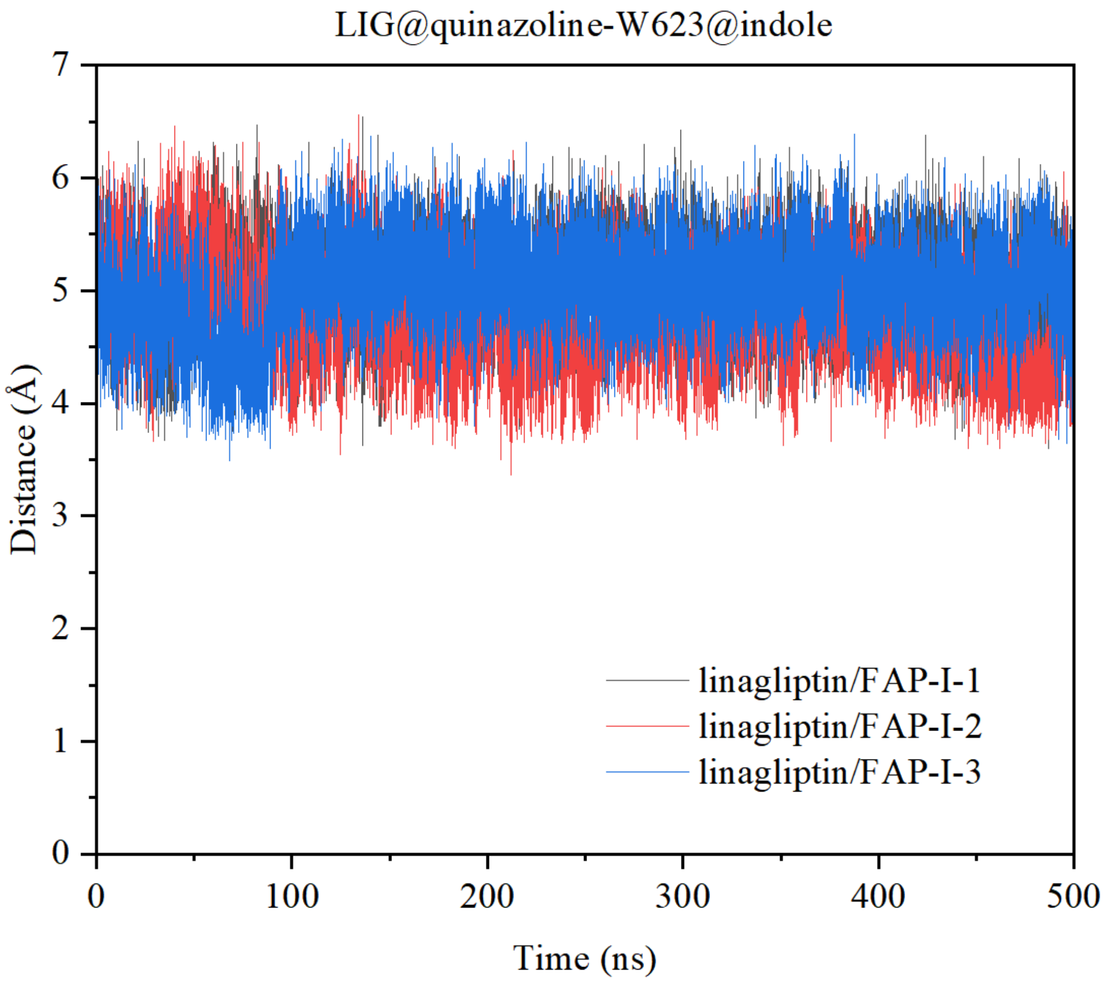


**Figure S30. Distance between quinazoline ring of linagliptin and indole ring of W623 for linagliptin/FAP-I systems although the 500 ns MD simulation**

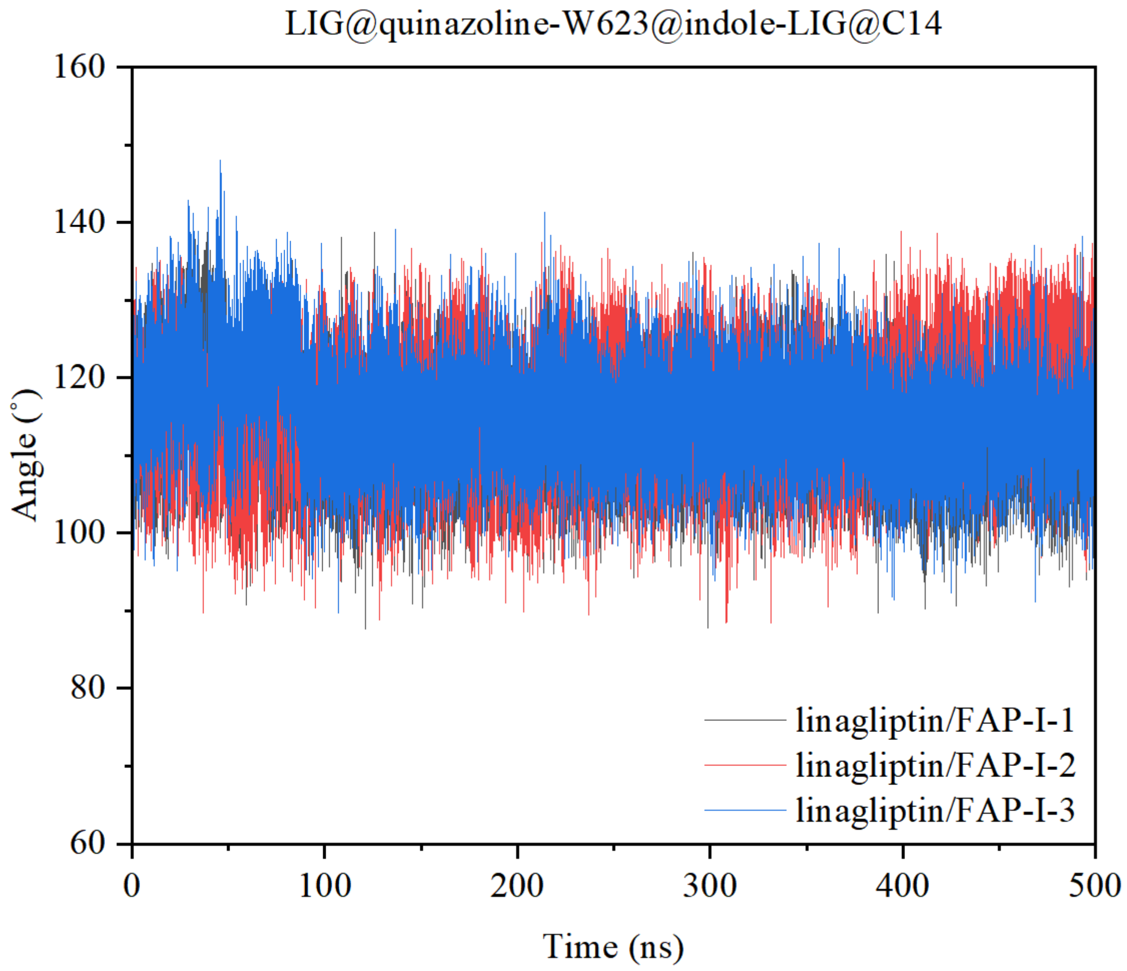


**Figure S31. Angle among quinazoline ring of linagliptin, indole ring of W623, and C14 atom of linagliptin for linagliptin/FAP-I systems although the 500 ns MD simulation**

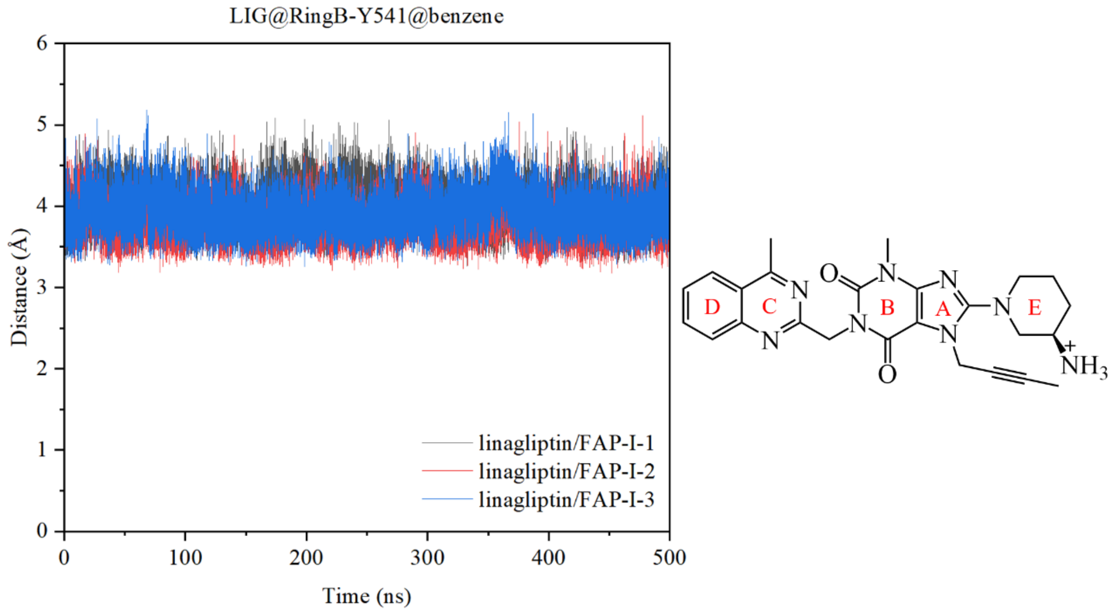


**Figure S32. Distance between ring B of linagliptin and benzene ring of Y541 for linagliptin/FAP-I systems although the 500 ns MD simulation**

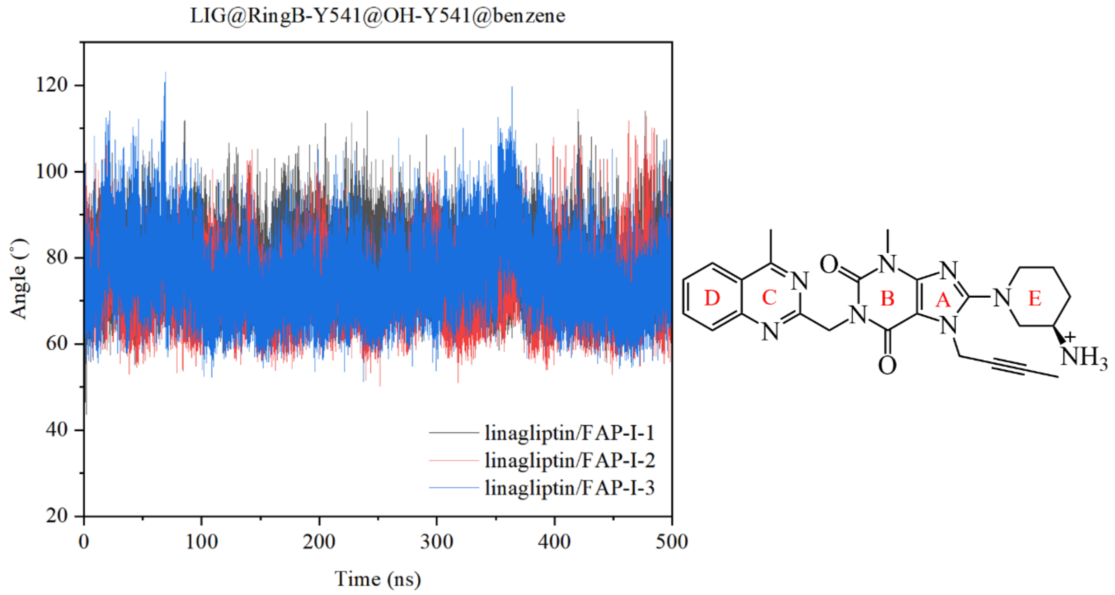


**Figure S33. Angle among ring B of linagliptin, OH atom of Y541, and benzene ring of Y541 for linagliptin/FAP-I systems although the 500 ns MD simulation**

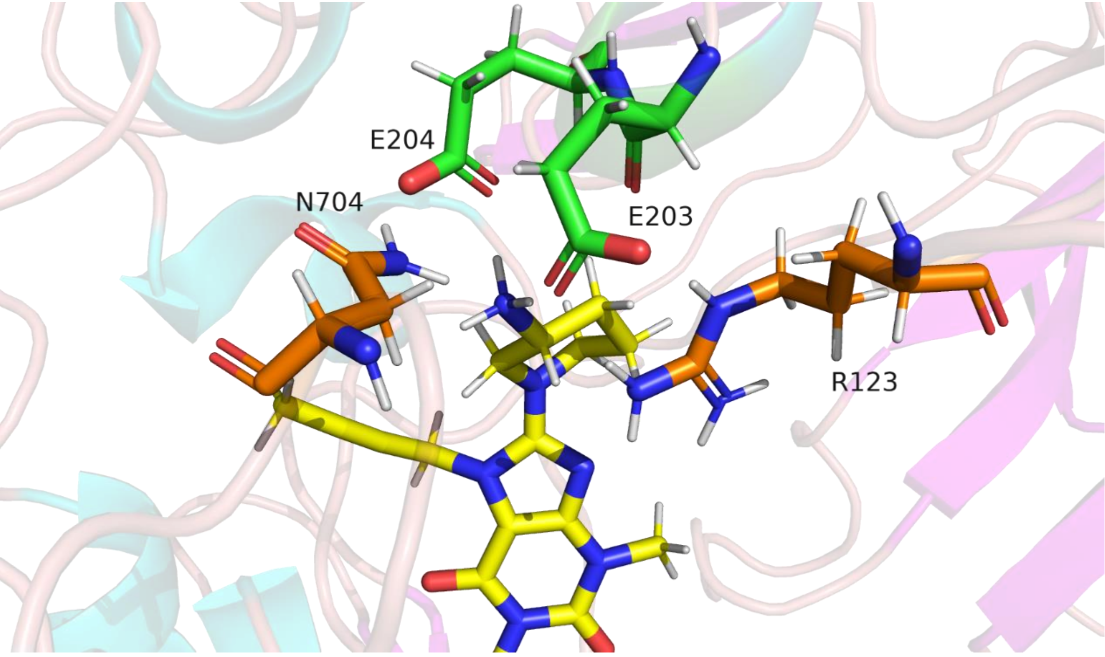


**Figure S34. R123 and N704 of FAP interact with linagliptin**

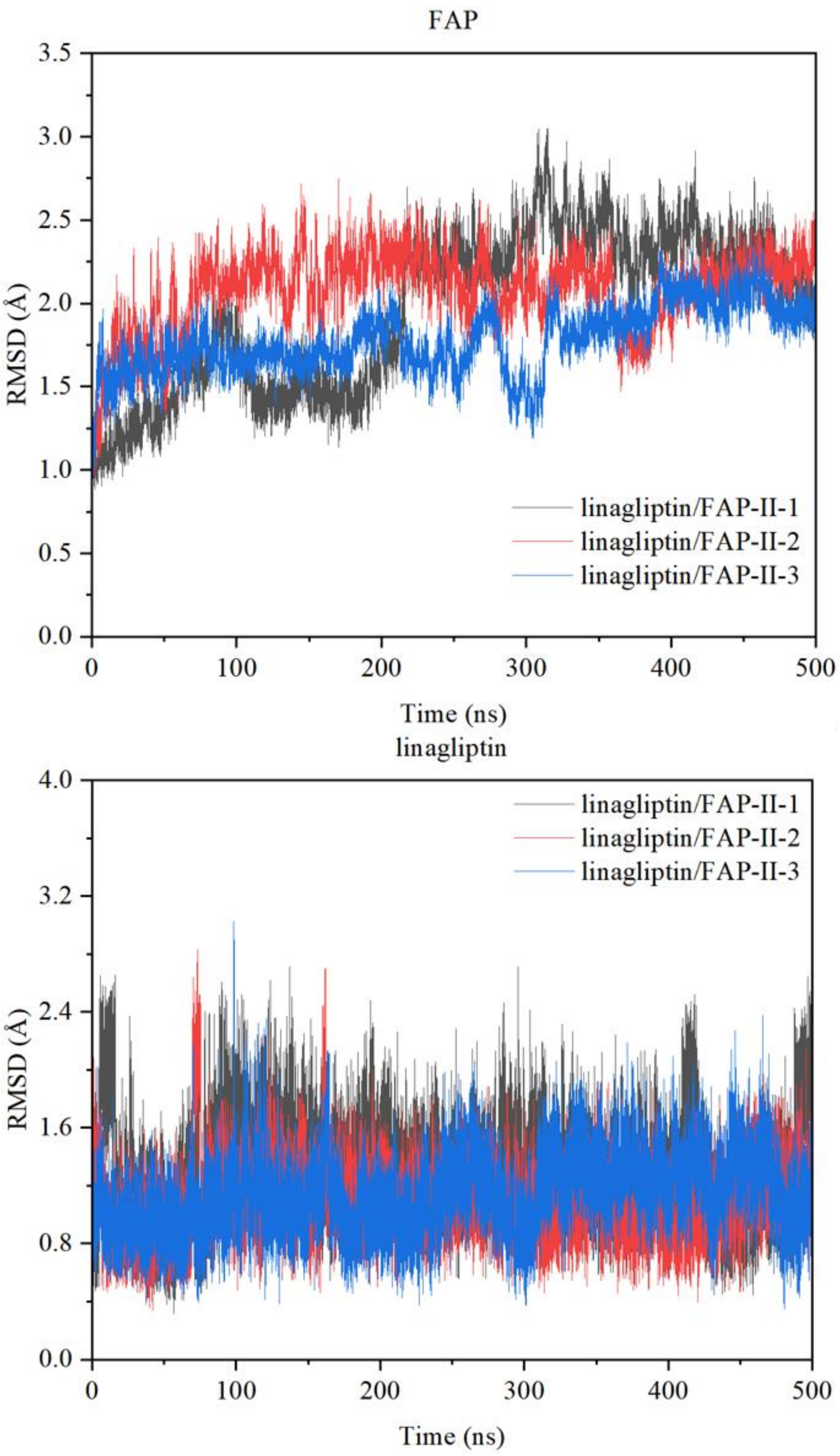


**Figure S35. Root mean square deviation (RMSD) vs Time plot for the 500 ns MD simulation on linagliptin/FAP-II**

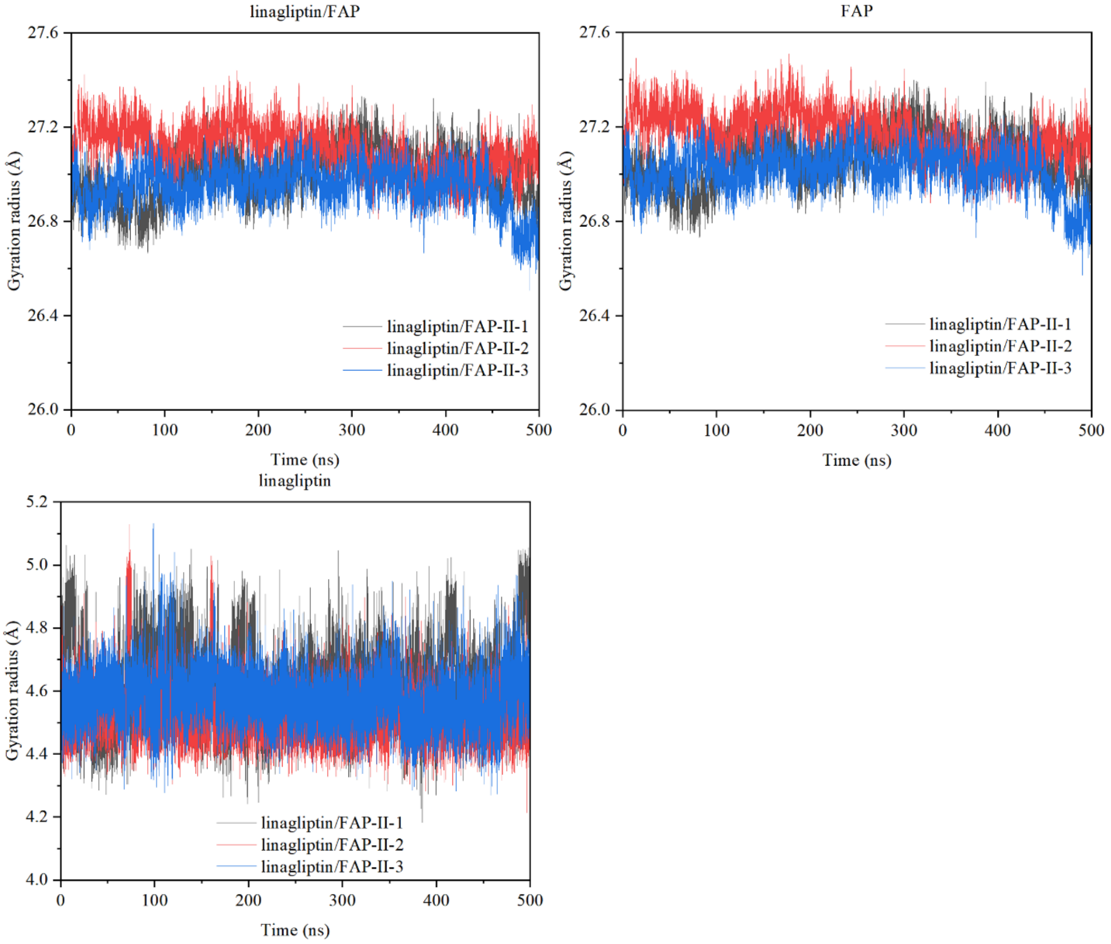


**Figure S36. Gyration radius vs Time plot for the 500 ns MD simulation on linagliptin/FAP-II**

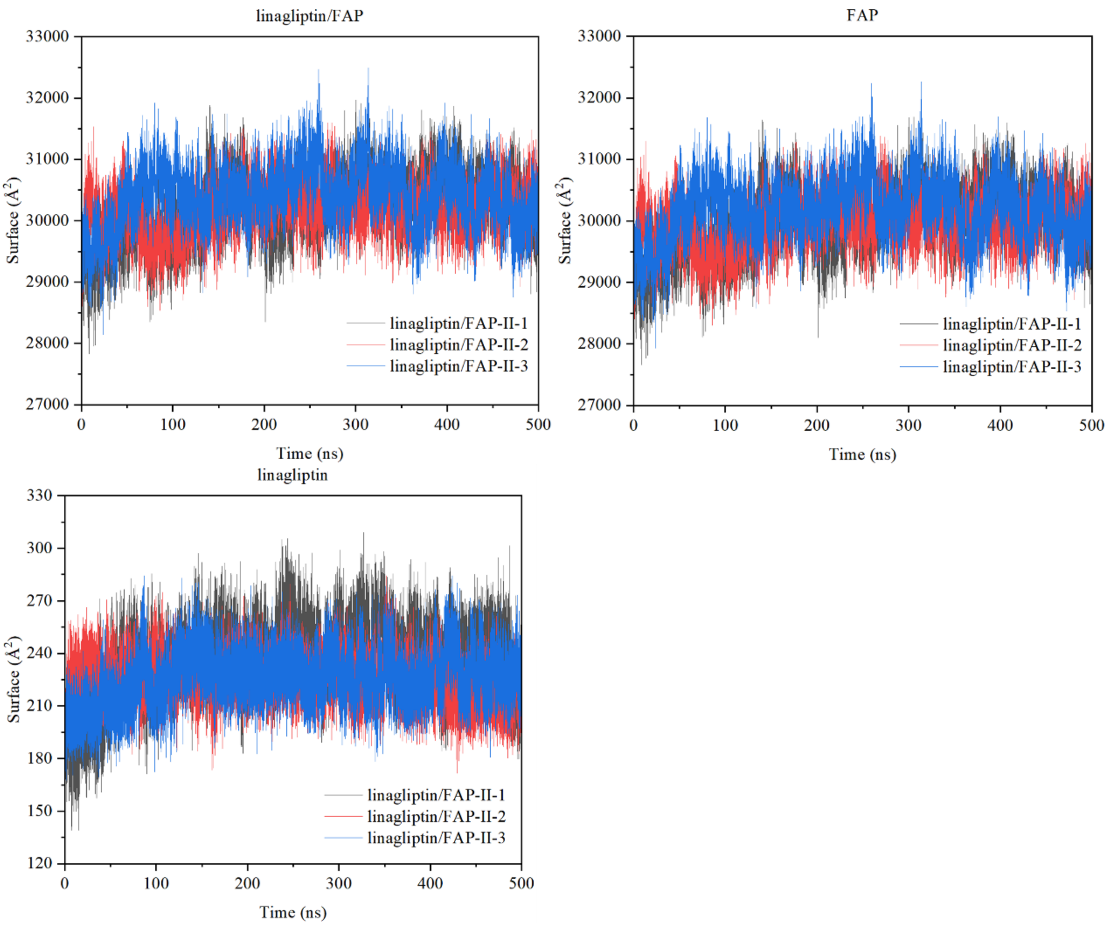


**Figure S37. Surface area vs Time plot for the 500 ns MD simulation on linagliptin/FAP-II**

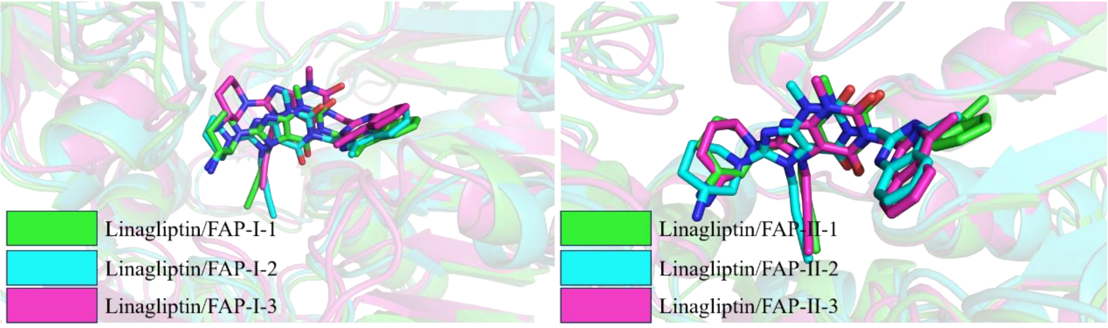


**Figure S38. Conformations for quinazoline group of linagliptin in linagliptin/FAP complex systems**

The 500$^{th}$ ns complex for every linagliptin/FAP complex systems was extracted. FAP shown with cartoon, and linagliptin shown with sticks.

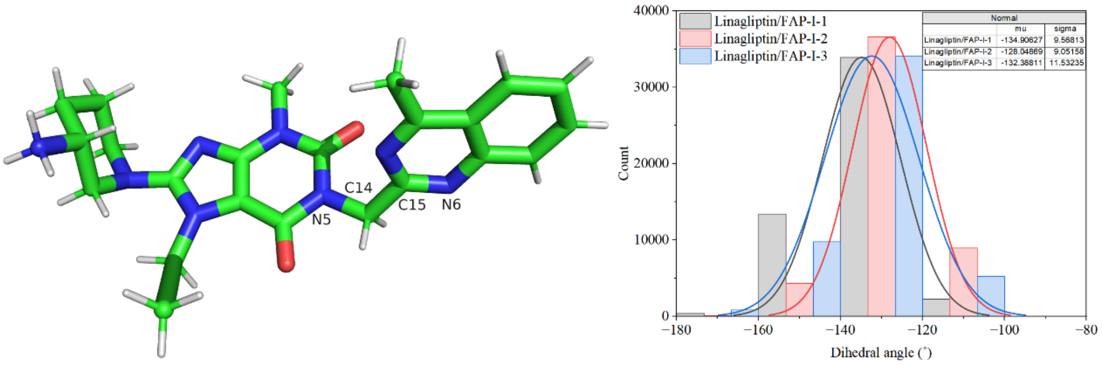


**Figure S39. Dihedral angle among N5, C14, C15 and N6 atoms for linagliptin/FAP-I**

There are 50000 conformations from the 500 ns simulations to analysis the dihedral angle among N5, C14, C15, and N6.

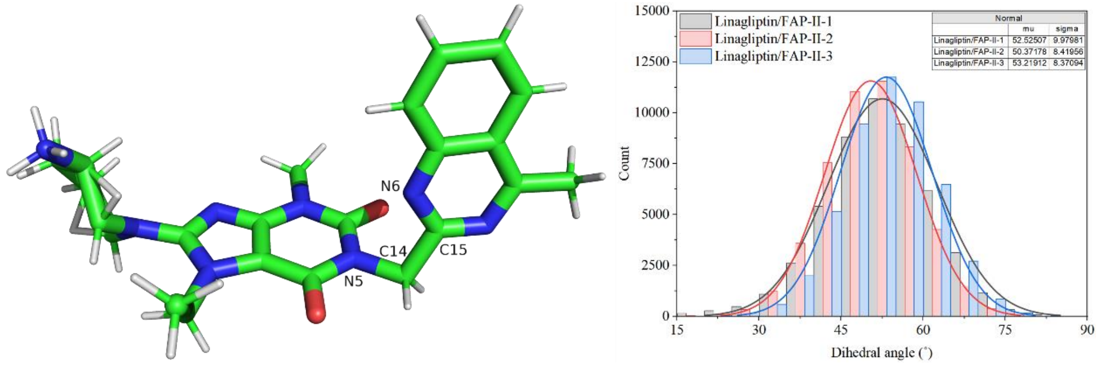


**Figure S40. Dihedral angle among N5, C14, C15 and N6 atoms for linagliptin/FAP-II**

There are 50000 conformations from the 500 ns simulations to analysis the dihedral angle among N5, C14, C15, and N6.

A

| Occupancy (%) | Linagliptin/FAP-II-1 | Linagliptin/FAP-II-2 | Linagliptin/FAP-II-3 |
|---|---|---|---|
| LIG@O1-Y625@N | 92.05 | 95.08 | 90.16 |
| E204@OE2-LIG@N3 | 93.16 | 88.63 | 72.91 |
| E204@OE1-LIG@N3 | 57.00 | 91.56 | 91.91 |
| E203@OE2-LIG@N3 | 63.90 | 9.95 | 26.53 |
| Y656@OH-LIG@N3 | 6.44 | 26.09 | 6.71 |
| E203@O-LIG@N3 | 78.41 | 24.91 | 32.28 |

B

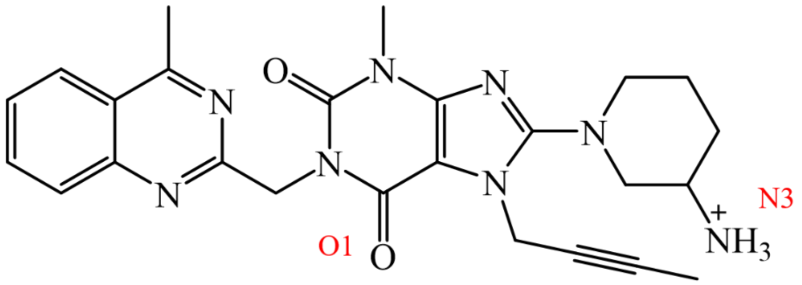


**Figure S41. Hydrogen bond analysis for linagliptin/FAP-II system**

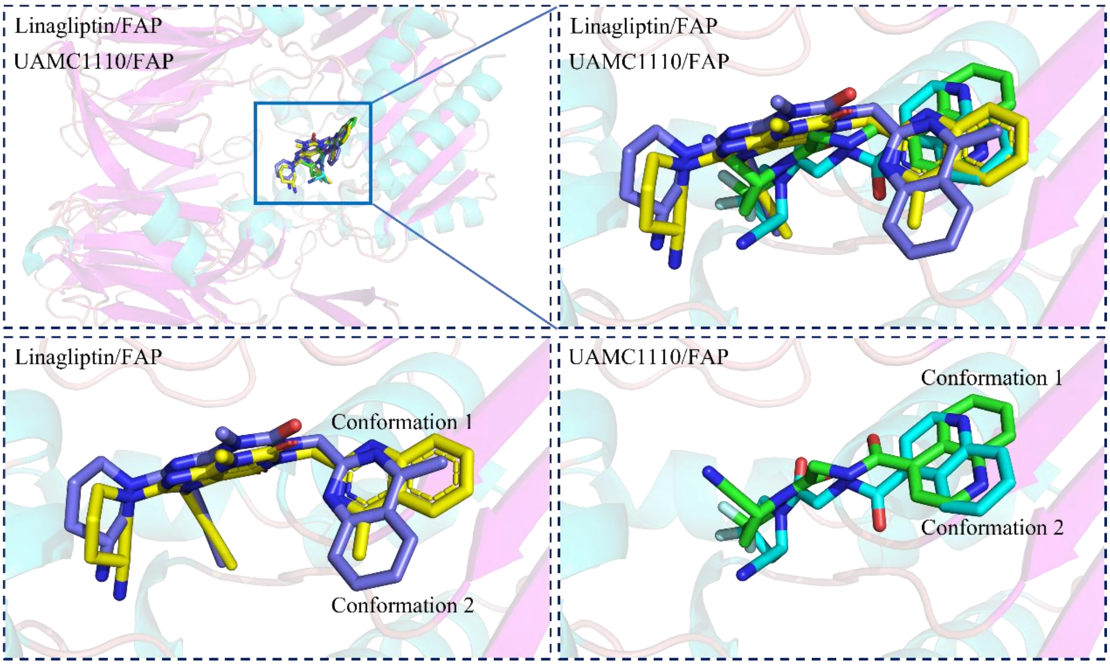


**Figure S42. Conformation analysis for UAMC1110 binding with FAP from molecular docking**

Molecular docking was performed with UAMC1110 binding with human FAP, which based on the binding model of linagliptin/FAP. The two conformations of quinoline group also have been found for UAMC1110 binding with FAP.

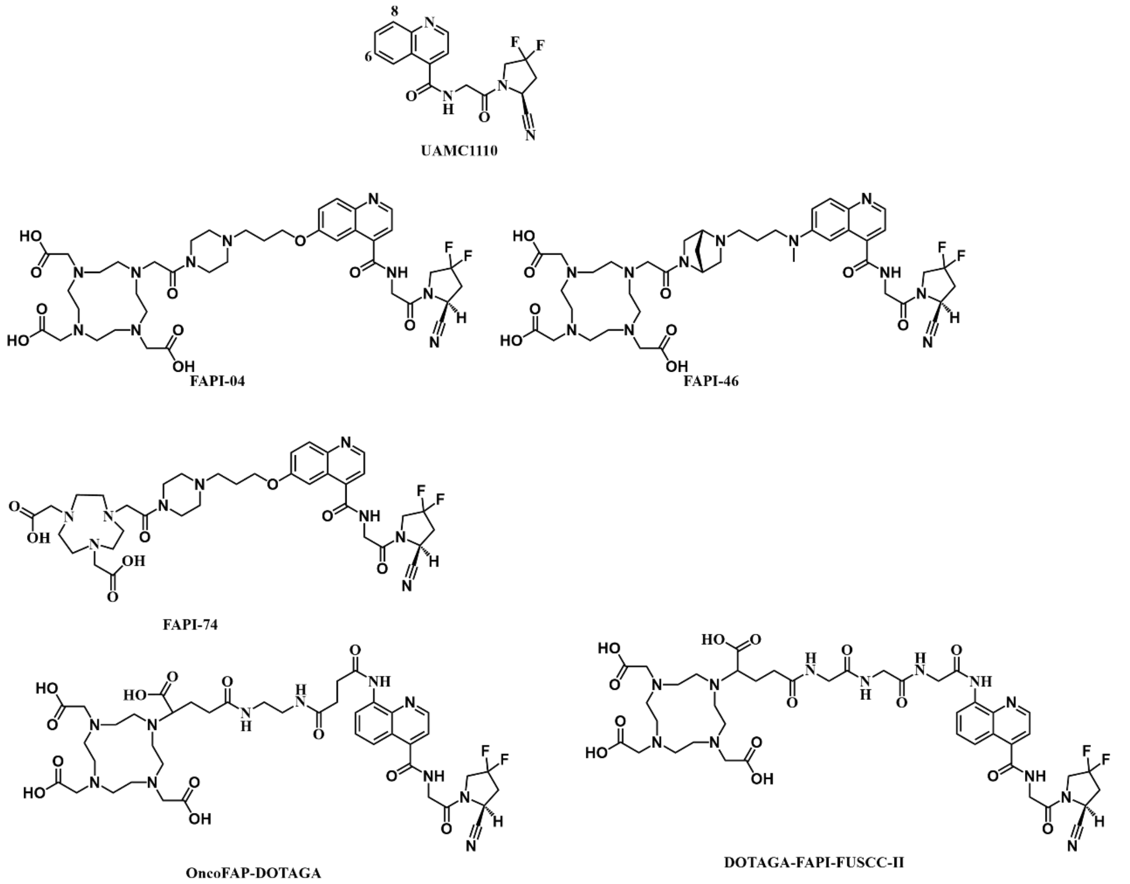


**Figure S43. Quinazoline ring of UAMC1110 are applied to design targeting FAP radiopharmaceutical**

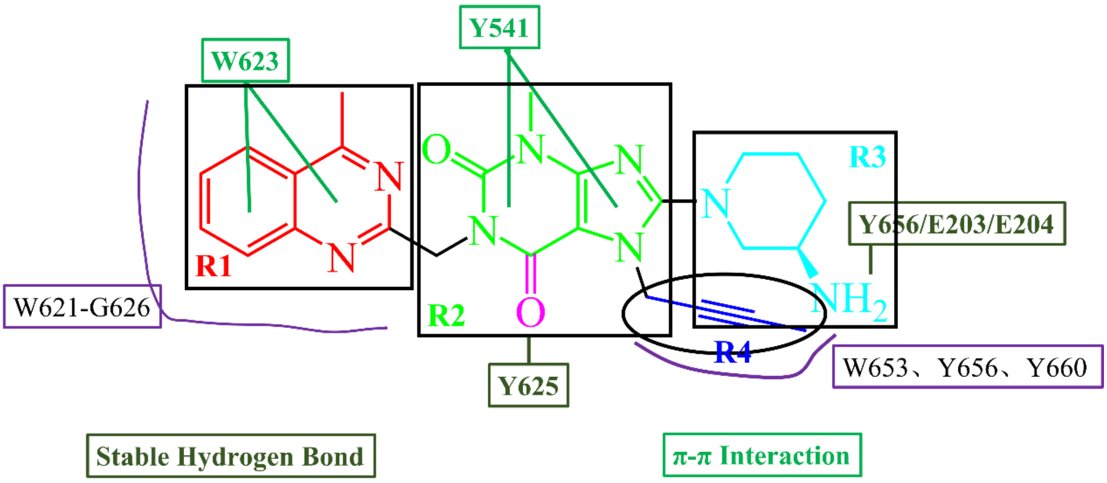


**Figure S44. Binding model for linagliptin with human FAP**

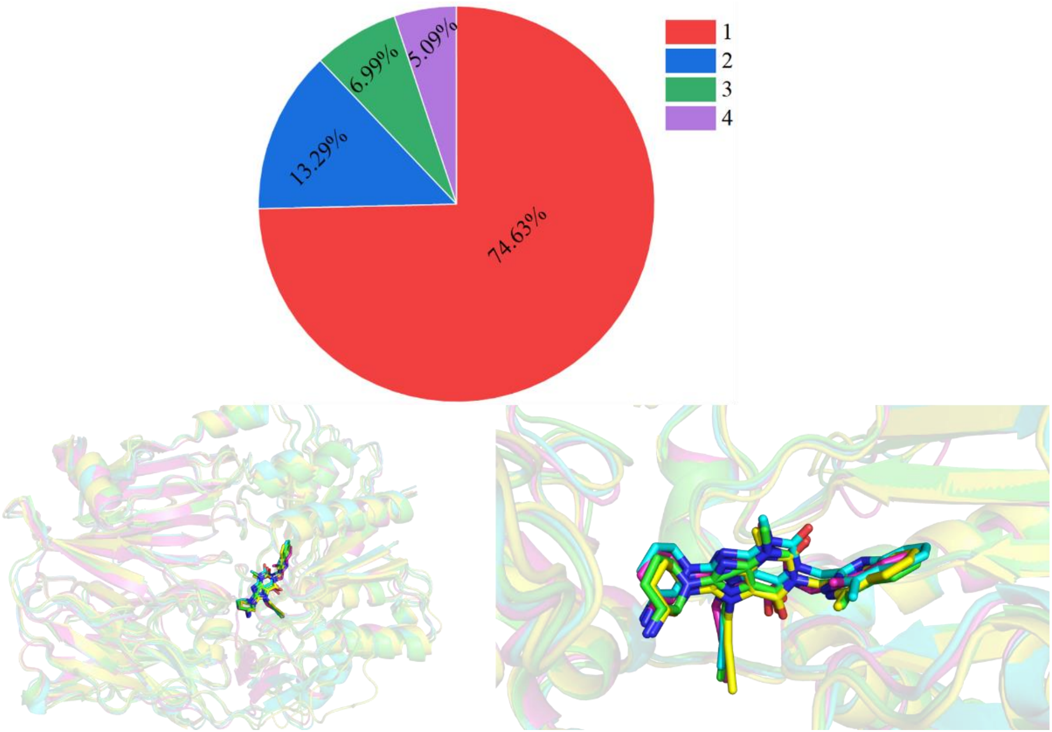


**Figure S45. Cluster analysis for linagliptin/FAP-I-1 system**

This cluster analysis was based on the 2000 frames extracted from the last 200 ns simulation and on the epsilon = 2.0 Å.

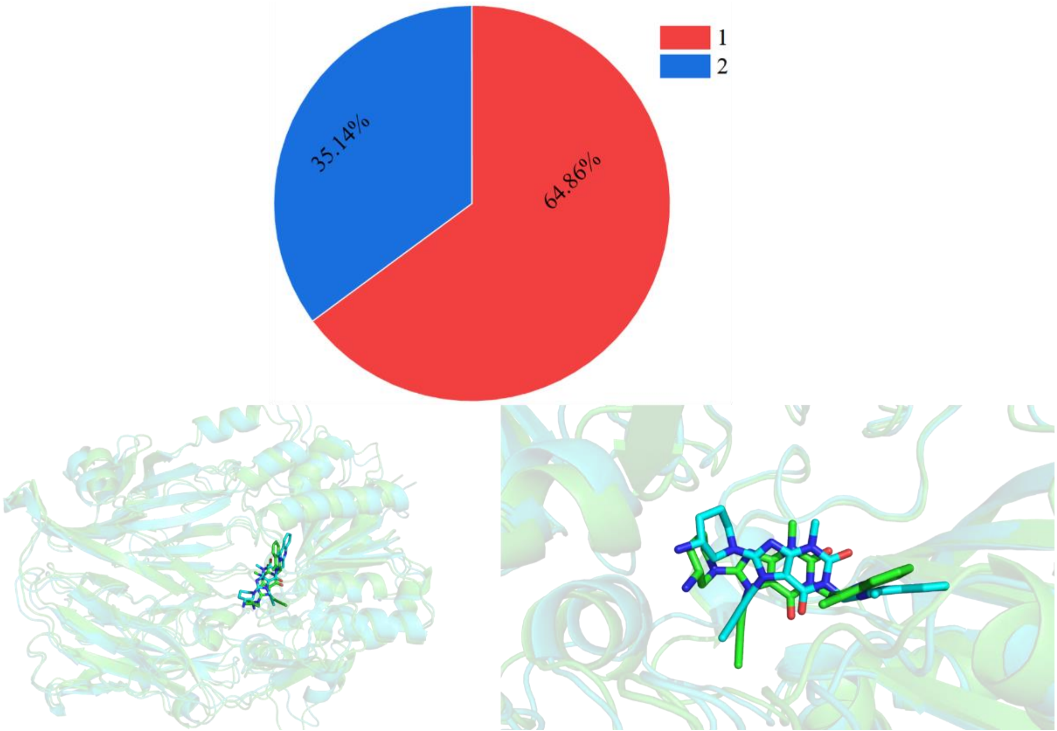


**Figure S46. Cluster analysis for linagliptin/FAP-I-2 system**

This cluster analysis was based on the 2000 frames extracted from the last 200 ns simulation and on the epsilon = 2.0 Å.

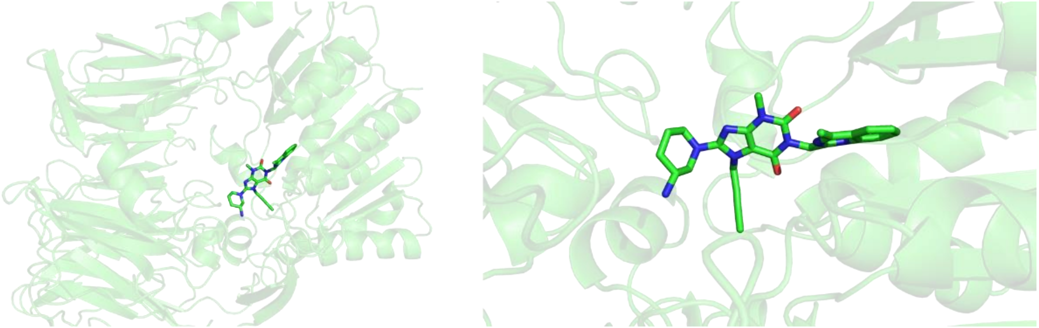

**Figure S47. Cluster analysis for linagliptin/FAP-I-3 system**

This cluster analysis was based on the 2000 frames extracted from the last 200 ns simulation and on the epsilon = 2.0 Å.

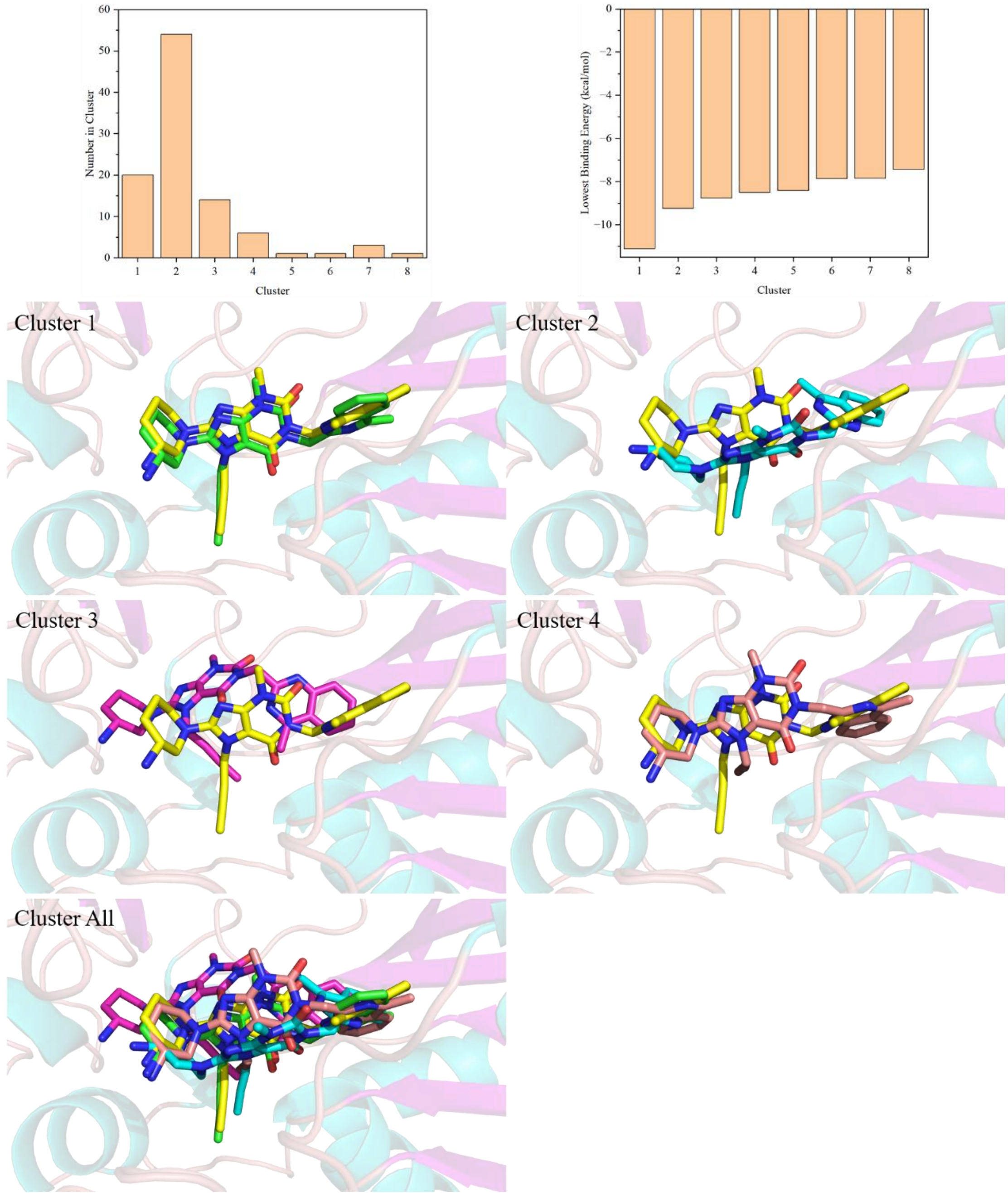


**Figure S48. Docking analysis for linagliptin/FAP-I-1 system**

There are 100 conformations which were generated from molecular docking. The RMSD value between the representative conformation from the first cluster (colored with C: yellow, N: blue, and O: red) and the conformation of the lowest binding energy from molecular docking is 1.03 Å and the binding energy is −11.11 kcal/mol.

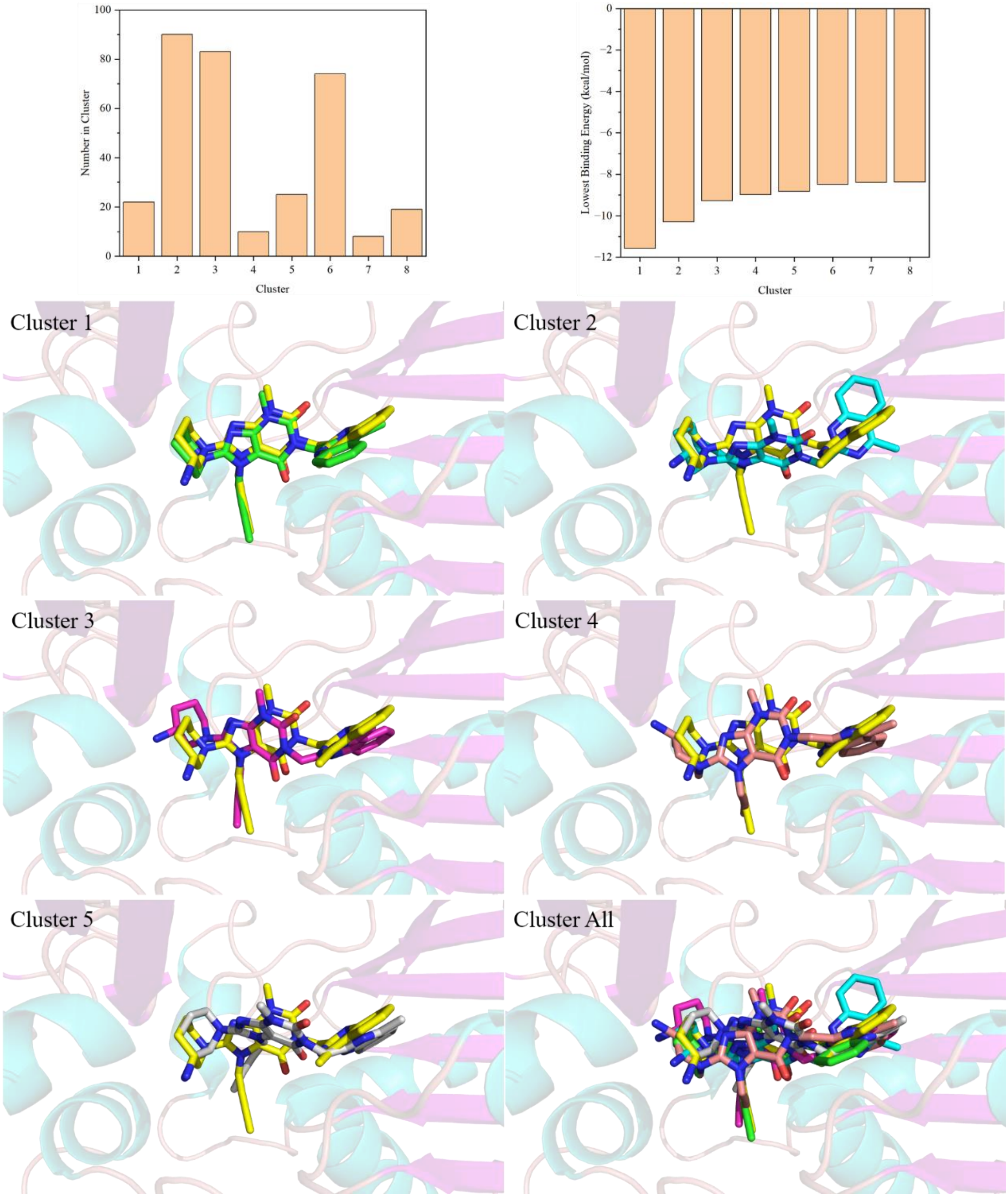


**Figure S49. Docking analysis for linagliptin/FAP-I-2 system**

There are 100 conformations which were generated from molecular docking. The RMSD value between the representative conformation from the first cluster (colored with C: yellow, N: blue, and O: red) and the conformation of the lowest binding energy from molecular docking is 0.66 Å and the binding energy is −11.58 kcal/mol.

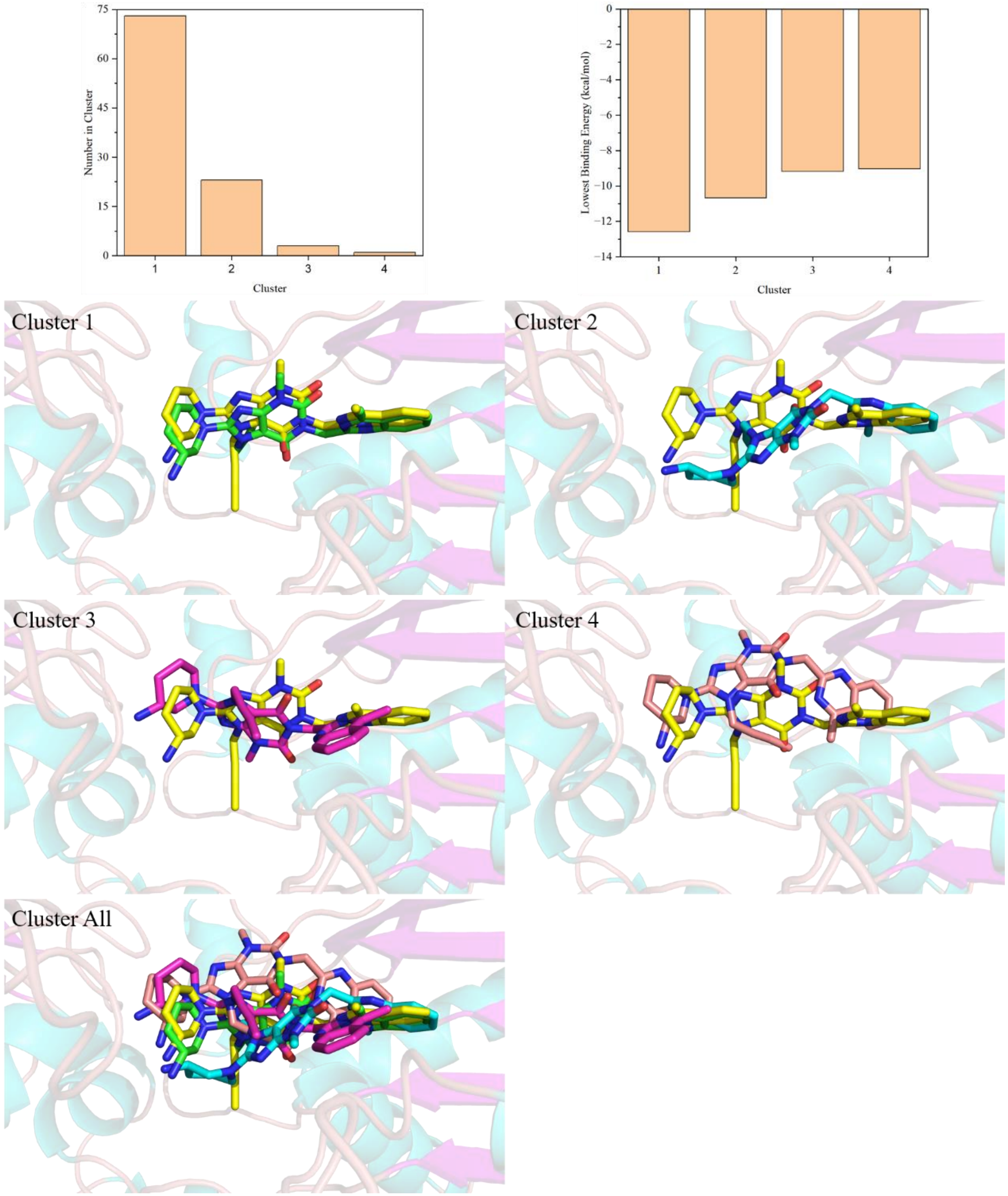


**Figure S50. Docking analysis for linagliptin/FAP-I-3 system**

There are 100 conformations which were generated from molecular docking. The RMSD value between the representative conformation from the first cluster (colored with C: yellow, N: blue, and O: red) and the conformation of the lowest binding energy from molecular docking is 1.12 Å and the binding energy is −12.59 kcal/mol.

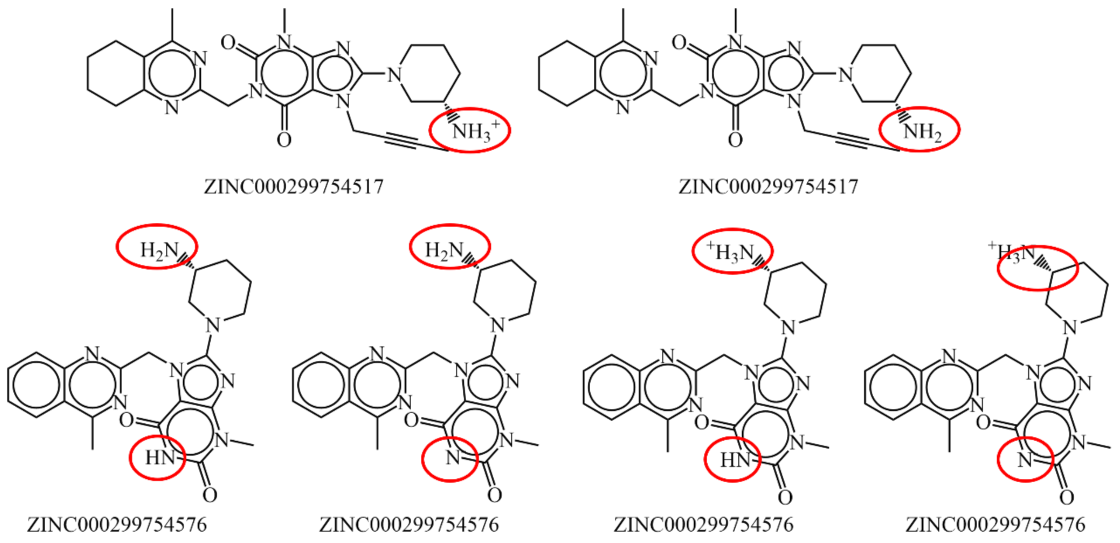


**Figure S51. Ionization states of ZINC000299754517 and ZINC000299754576 from prediction with Dimorphite-DL**

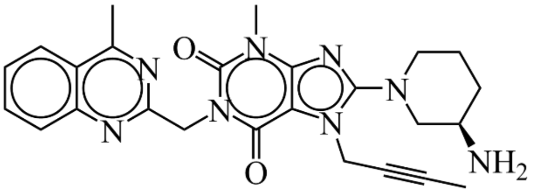

ZINC000003820029

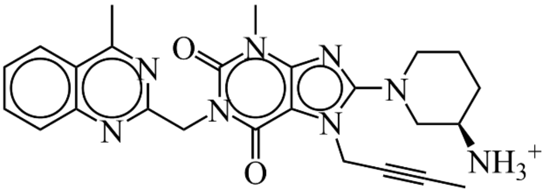

ZINC000003820029

| Name | Lowest binding energy | | | Binding energy |
|---|---|---|---|---|
| | kcal/mol | | | kcal/mol |
| | FAP-I-1 | FAP-I-2 | FAP-I-3 | |
| **Linagliptin** | -11.11 | -11.58 | -12.59 | -12.59 |
| **ZINC000003820029 (-$NH_3^+$)** | -10.11 | -10.20 | -11.41 | -11.41 |
| **ZINC000003820029 (-$NH_2$)** | -8.93 | -10.70 | -10.23 | -10.70 |

**Figure S52. Structure and docking score for linagliptin with different ionization states**

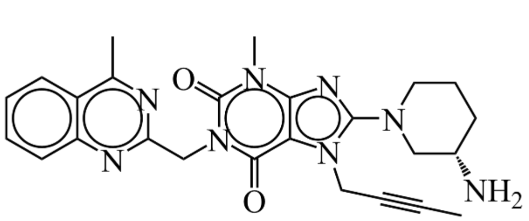

ZINC000059031486

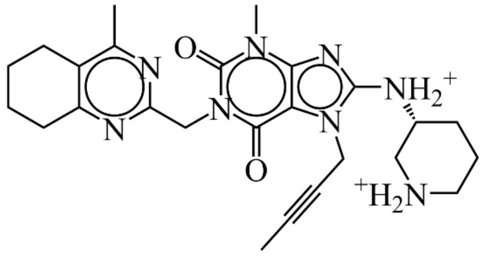

ZINC000299754593

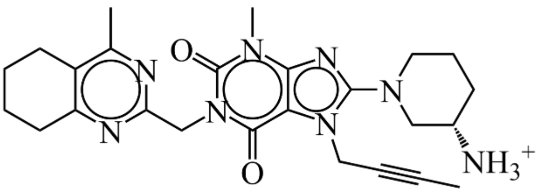

ZINC000299754517

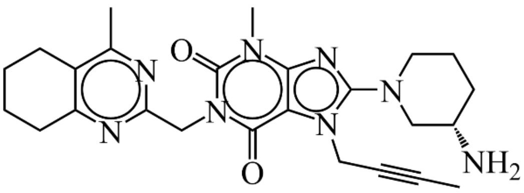

ZINC000299754517

| **Name** | **Lowest binding energy** | | | **Binding energy** |
|---|---|---|---|---|
| | kcal/mol | | | kcal/mol |
| | FAP-I-1 | FAP-I-2 | FAP-I-3 | |
| ZINC000059031486 | -9.09 | -10.12 | -11.40 | -11.40 |
| ZINC000299754517 (+1) | -9.38 | -9.29 | -11.13 | -11.13 |
| ZINC000299754517 | -9.31 | -10.90 | -10.70 | -10.90 |
| ZINC000299754593 | -9.13 | -10.75 | -10.94 | -10.94 |

**Figure S53. Structure and docking score for the top four compounds**

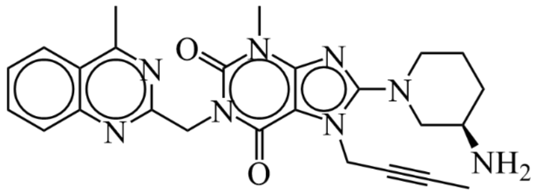

ZINC000003820029

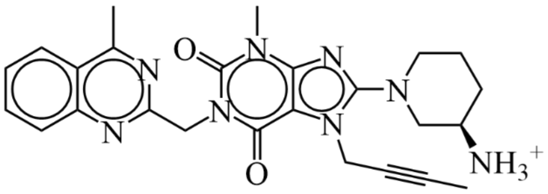

ZINC000003820029

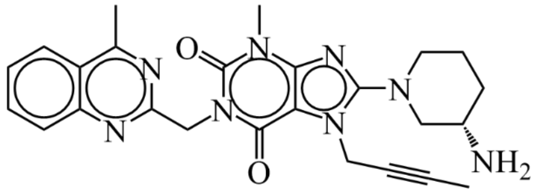

ZINC000059031486

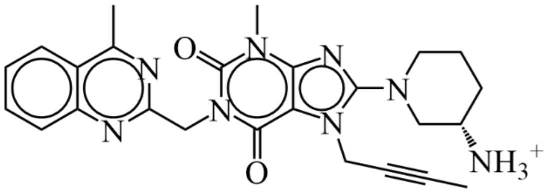

ZINC000059031486

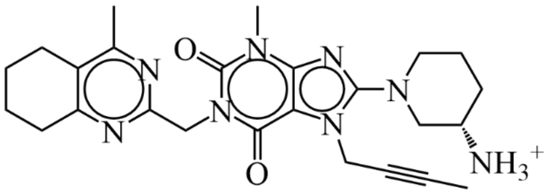

ZINC000299754517

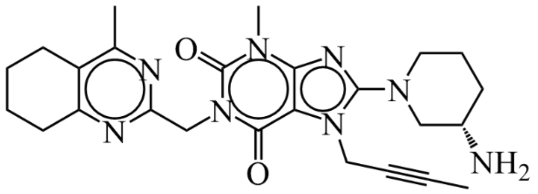

ZINC000299754517

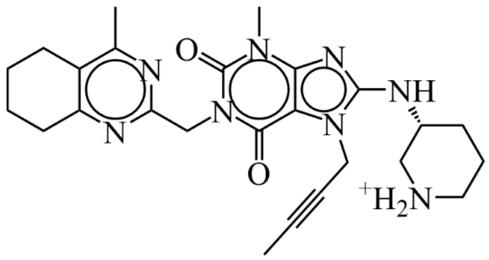

ZINC000299754593

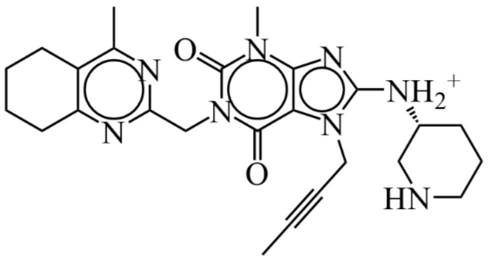

ZINC000299754593

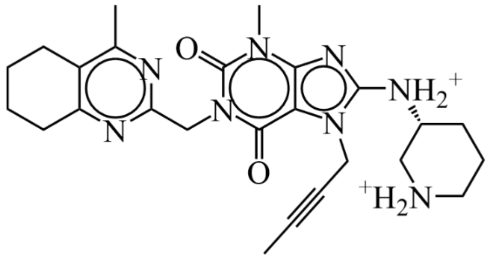

ZINC000299754593

**Figure S54. Class one small molecules as potential FAP inhibitors**

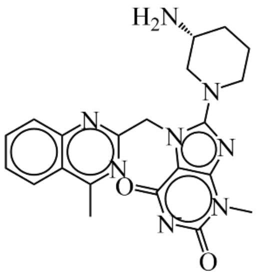

ZINC000299754576

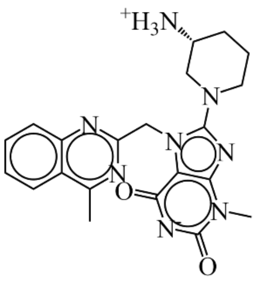

ZINC000299754576

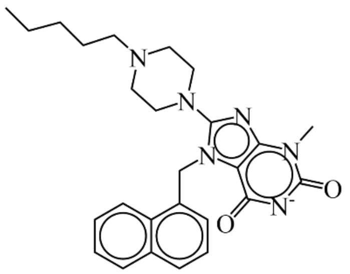

ZINC000009714540

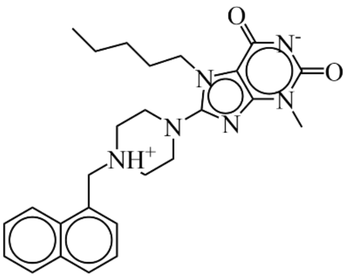

ZINC000022855256

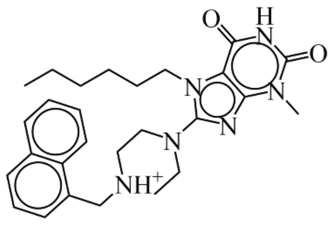

ZINC000022855258

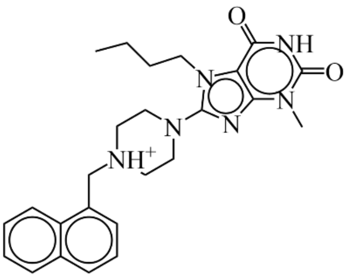

ZINC000022421631

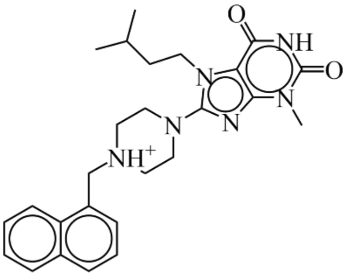

ZINC000022409193

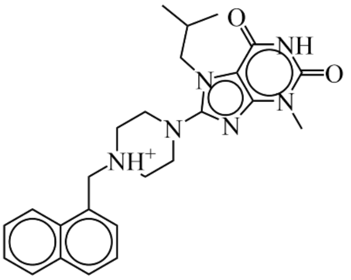

ZINC000020565829

**Figure S55. Class two small molecules as potential FAP inhibitors**

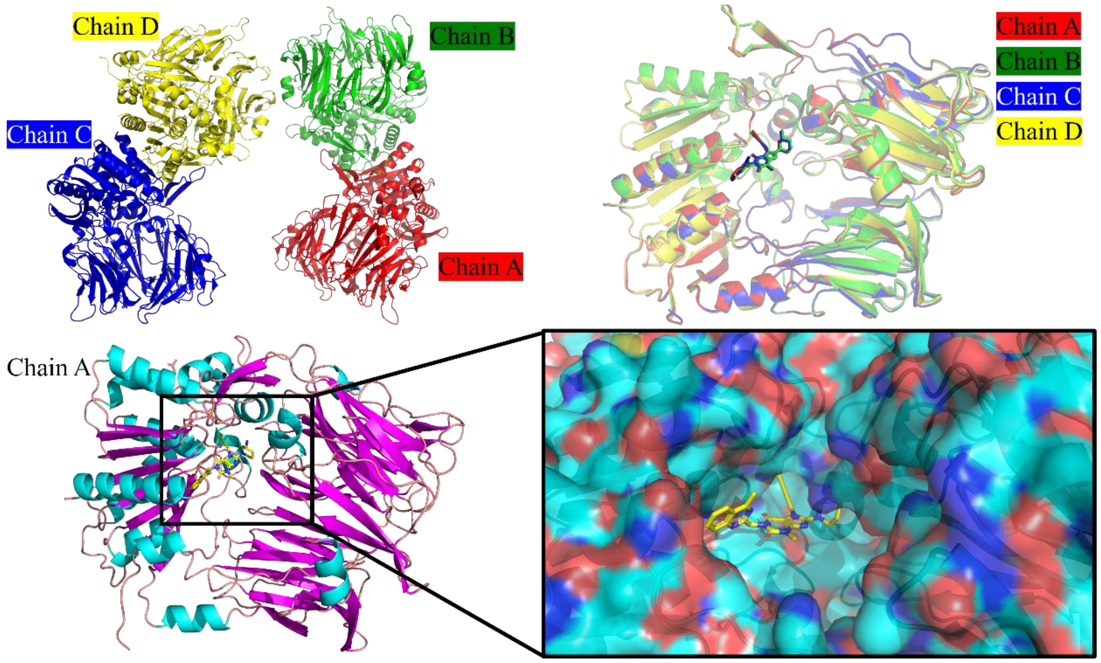


**Figure S56. Crystal structure for linagliptin binding with human fibroblast-activation protein**

Crystal structure obtained from Protein Data Bank with 6Y0F.

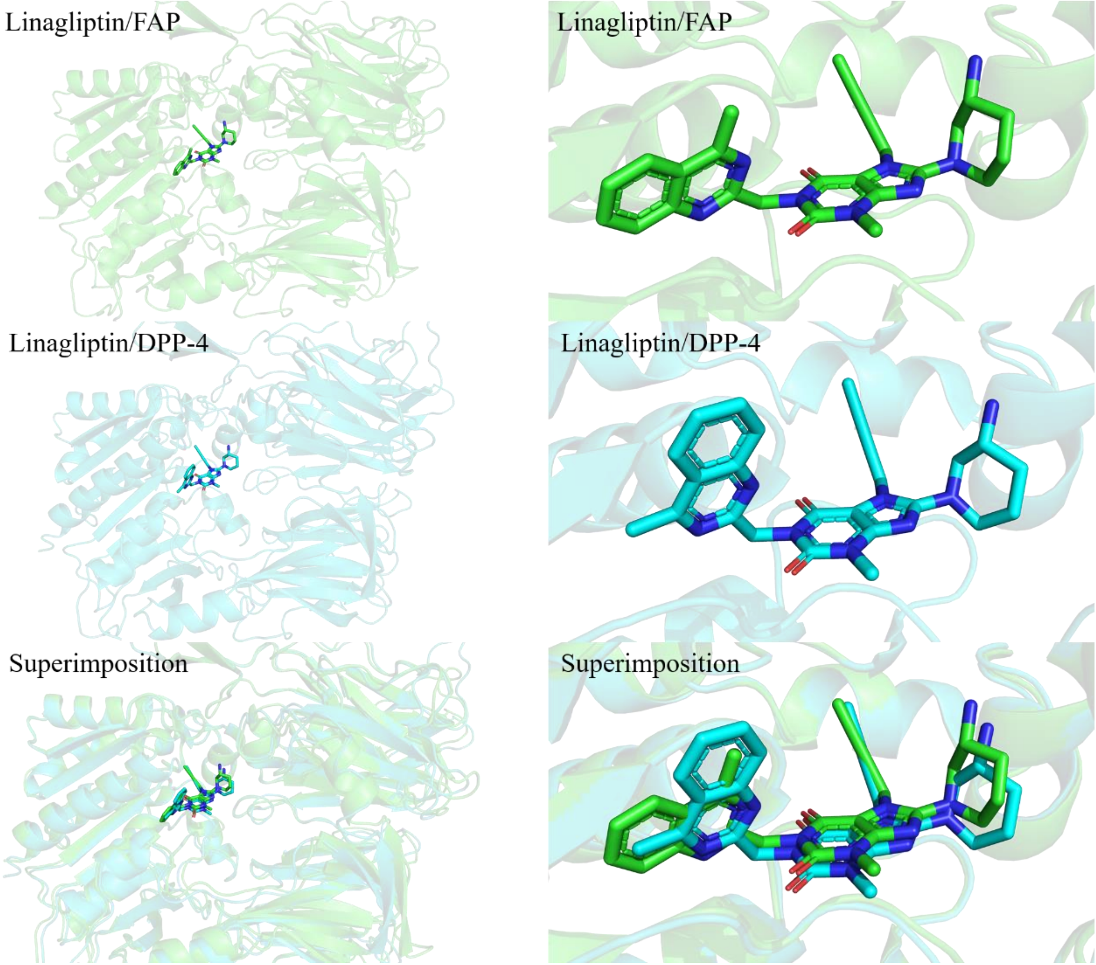


**Figure S57. Conformations of linagliptin binding with FAP and DPP-4**

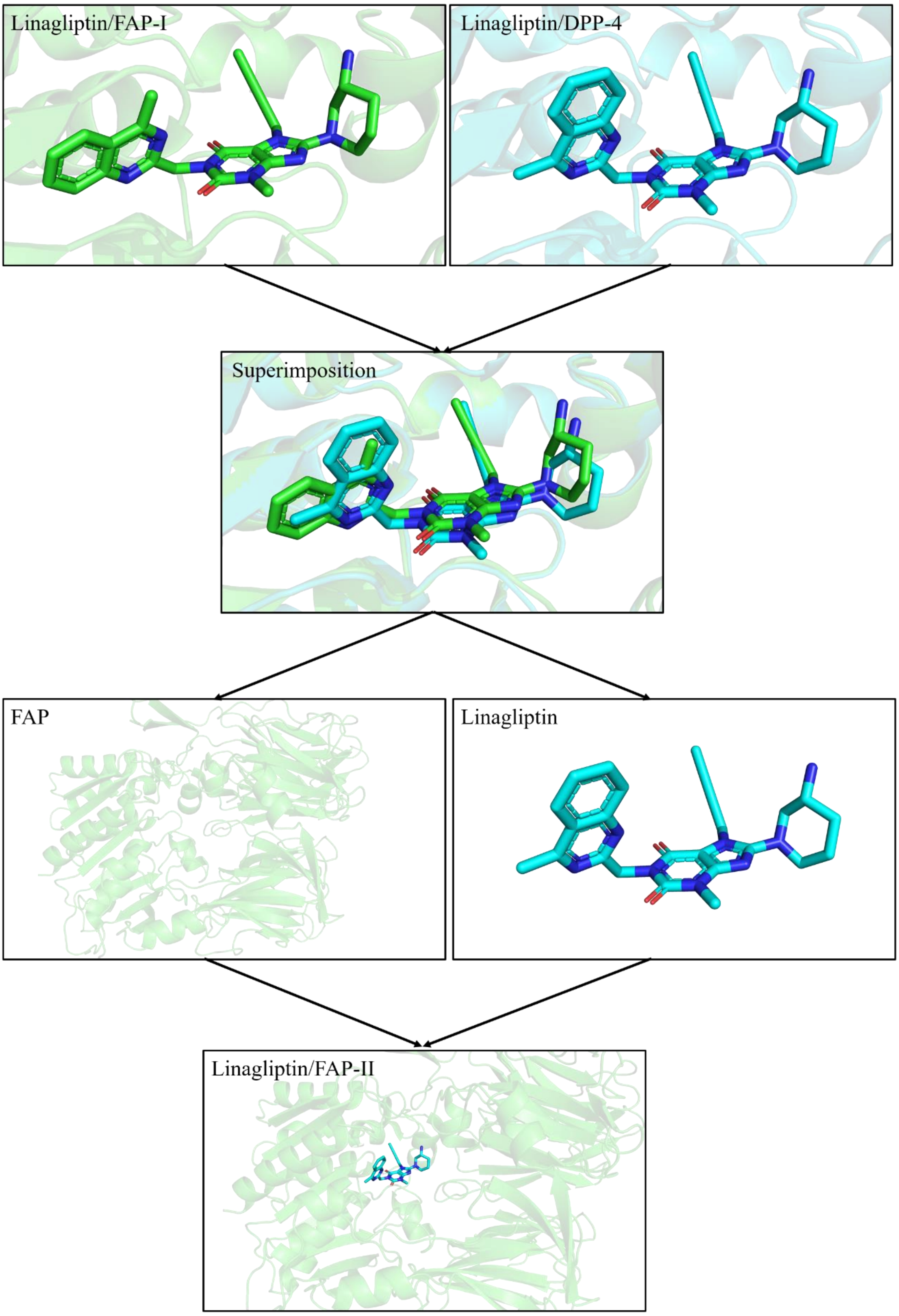


**Figure S58. Method for construction linagliptin/FAP-II system**

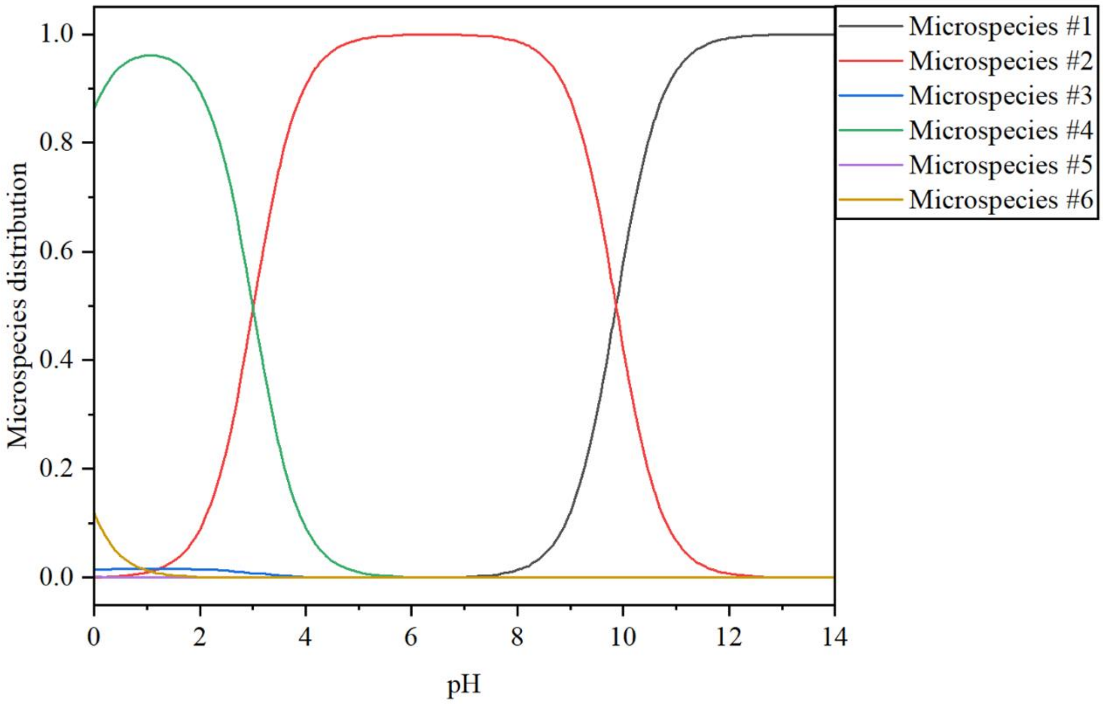


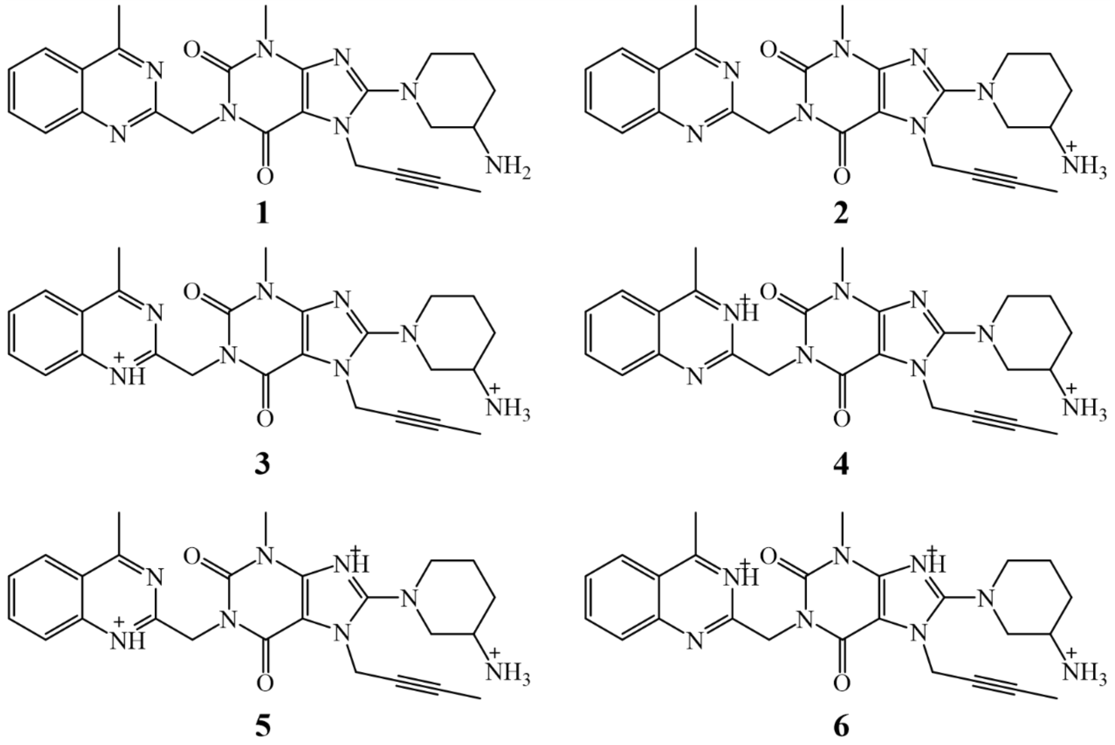


**Figure S59. Different charged state of linagliptin**

Calculator Plugins were used for structure property prediction and calculation, Marvin 22.13, 2022, ChemAxon (http://www.chemaxon.com)

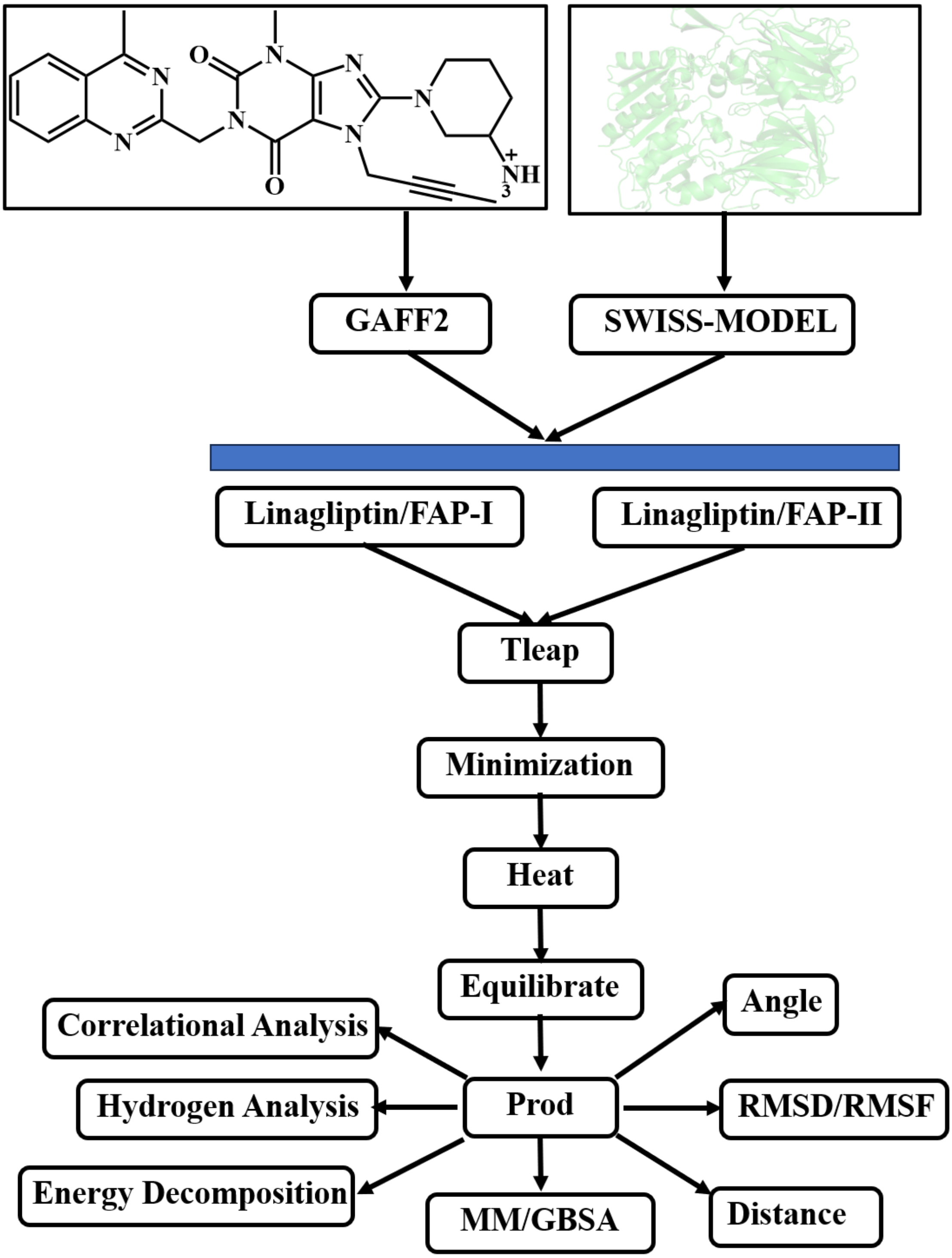


**Figure S60. Method for linagliptin/FAP systems**

**Table S1. RMSD value of the FAP and linagliptin for the linagliptin/FAP complex systems although 500 ns MD simulations**

| RMSD (Å) | FAP | Linagliptin |
|---|---|---|
| Linagliptin/FAP-I-1 | 1.61 ± 0.30* | 1.14 ± 0.22 |
| Linagliptin/FAP-I-2 | 2.11 ± 0.31 | 1.22 ± 0.29 |
| Linagliptin/FAP-I-3 | 1.67 ± 0.11 | 0.97 ± 0.27 |
| Linagliptin/FAP-II-1 | 1.96 ± 0.47 | 1.33 ± 0.35 |
| Linagliptin/FAP-II-1 | 2.08 ± 0.24 | 1.09 ± 0.26 |
| Linagliptin/FAP-II-1 | 1.78 ± 0.20 | 1.14 ± 0.27 |

*Data are represented as mean ± standard deviation.

**Table S2. Statistical hydrogen bond distance between linagliptin and FAP the linagliptin/FAP complex systems although 500 ns MD simulations**

| System | Hydrogen Bond Distance (Å) | | |
|---|---|---|---|
| | LIG@O1-Y625@N | E203@OE2-LIG@N3 | E204@OE1-LIG@N3 |
| Linagliptin/FAP-I-1 | 3.20 ± 0.20 | 3.34 ± 0.91 | 3.18 ± 0.49 |
| Linagliptin/FAP-I-2 | 3.17 ± 0.19 | 3.44 ± 0.93 | 3.33 ± 0.70 |
| Linagliptin/FAP-I-3 | 3.33 ± 0.25 | 2.79 ± 0.16 | 2.84 ± 0.20 |
| | E204@OE2-LIG@N3 | Y656@OH-LIG@N3 | |
| Linagliptin/FAP-I-1 | 2.79 ± 0.15 | 3.27 ± 0.51 | |
| Linagliptin/FAP-I-2 | 2.85 ± 0.21 | 3.75 ± 0.87 | |
| Linagliptin/FAP-I-3 | 2.95 ± 0.20 | 5.04 ± 0.45 | |

Data are represented as mean ± standard deviation.

**Table S3. Statistical hydrogen bond angle between linagliptin and FAP the linagliptin/FAP complex systems although 500 ns MD simulations**

| System | Hydrogen Bond Angle (˚) | | | |
|---|---|---|---|---|
| | LIG@O1-Y625@H-Y625@N | E203@OE2-LIG@H13-LIG@N3 | E203@OE2-LIG@H14-LIG@N3 | E203@OE2-LIG@H15-LIG@N3 |
| Linagliptin/FAP-I-1 | 148.60 ± 11.44 | 91.62 ± 44.85 | 94.41 ± 46.49 | 93.57 ± 46.89 |
| Linagliptin/FAP-I-2 | 148.11 ± 11.30 | 92.85 ± 48.19 | 91.27 ± 46.52 | 91.22 ± 46.52 |
| Linagliptin/FAP-I-3 | 142.93 ± 11.58 | 92.06 ± 46.88 | 89.78 ± 46.82 | 88.77 ± 45.98 |
| | E204@OE1-LIG@H13-LIG@N3 | E204@OE1-LIG@H14-LIG@N3 | E204@OE1-LIG@H15-LIG@N3 | |
| Linagliptin/FAP-I-1 | 83.52 ± 48.36 | 82.66 ± 47.50 | 86.45 ± 49.23 | |
| Linagliptin/FAP-I-2 | 85.24 ± 48.87 | 87.94 ± 48.98 | 84.17 ± 19.01 | |
| Linagliptin/FAP-I-3 | 87.18 ± 50.72 | 91.50 ± 52.23 | 89.49 ± 51.71 | |
| | E204@OE2-LIG@H13-LIG@N3 | E204@OE2-LIG@H14-LIG@N3 | E204@OE2-LIG@H15-LIG@N3 | |
| Linagliptin/FAP-I-1 | 91.56 ± 37.96 | 90.37 ± 37.39 | 92.82 ± 37.90 | |
| Linagliptin/FAP-I-2 | 89.86 ± 37.76 | 94.59 ± 39.99 | 89.93 ± 38.20 | |
| Linagliptin/FAP-I-3 | 90.29 ± 26.45 | 92.25 ± 27.19 | 92.07 ± 26.78 | |
| | Y656@OH-LIG@H13-LIG@N3 | Y656@OH-LIG@H14-LIG@N3 | Y656@OH-LIG@H15-LIG@N3 | |
| Linagliptin/FAP-I-1 | 89.52 ± 39.08 | 87.30 ± 38.73 | 86.97 ± 36.88 | |
| Linagliptin/FAP-I-2 | 90.17 ± 40.33 | 91.87 ± 40.18 | 93.32 ± 39.90 | |
| Linagliptin/FAP-I-3 | 97.98 ± 40.54 | 96.73 ± 39.81 | 100 ± 40.59 | |

Data are represented as mean ± standard deviation.

**Table S4. Binding free energies, decomposition and electrostatic interactions ($E_{ele}$), van der Waals interactions ($E_{vdW}$), solvation free energies ($E_{polar}$), nonpolar solvation energies ($E_{nonpolar}$), and entropy ($TS_{total}$)[#] of the linagliptin/FAP-I systems**

| Energy (kcal/mol) | Linagliptin/FAP-I-1 | Linagliptin/FAP-I-2 | Linagliptin/FAP-I-3 |
|---|---|---|---|
| $\Delta E_{vdW}$ | −52.02 | −52.56 | −53.97 |
| $\Delta E_{ele}$ | −208.66 | −204.50 | −234.72 |
| $\Delta E_{polar}$ | 214.83 | 210.87 | 242.74 |
| $\Delta E_{nonpolar}$ | −5.53 | −5.32 | −5.85 |
| $\Delta E_{gas}$ | −260.67 | −257.05 | −288.68 |
| $\Delta E_{solv}$ | 209.30 | 205.55 | 236.89 |
| $\Delta E_{gas} + \Delta E_{sol}$ | −51.37 | −51.51 | −51.79 |
| $T\Delta S_{total}$ | −37.65 | −37.95 | −38.10 |
| $\Delta G_{bind}^{cal}$ | −13.72 | −13.55 | −13.69 |

**Table S5. Binding free energies ($\Delta G_{bind}^{cal}$) for linagliptin/FAP-I-1 complex system**

| Energy (kcal/mol) | Complex | Receptor | Ligand | Delta |
|---|---|---|---|---|
| $E_{vdW}$ | −6142.91 | −6083.43 | −7.47 | −52.02 |
| $E_{ele}$ | −51831.71 | −51579.16 | −43.89 | −208.66 |
| $E_{polar}$ | −7955.35 | −8091.57 | −78.61 | 214.83 |
| $E_{nonpolar}$ | 217.33 | 218.38 | 4.48 | −5.53 |
| $E_{gas}$ | −12683.62 | −12216.29 | −206.66 | −260.67 |
| $E_{solv}$ | −7738.02 | −7873.19 | −74.13 | 209.30 |
| $E_{gas} + E_{sol}$ | −20421.64 | −20089.48 | −280.79 | −51.37 |
| $TS_{total}$ | 71.93 | 71.92 | 37.66 | −37.65 |
| $\Delta G_{bind}^{cal}$ | | | | −13.72 |

The binding free energies ($\Delta G_{bind}^{cal}$) for linagliptin/FAP complex and decomposition to electrostatic interaction ($E_{ele}$), van der Walls interaction ($E_{vdW}$), polar solvation free energies ($E_{polar}$), nonpolar solvation free energies ($E_{nonpolar}$), and entropy ($TS_{total}$). Energy values are presented in kcal/mol. Uncertainties were calculated as the root mean square error for all frames extracted from the trajectories.

**Table S6. Binding free energies ($\Delta G_{bind}^{cal}$) for linagliptin/FAP-I-2 complex system**

| Energy (kcal/mol) | Complex | Receptor | Ligand | Delta |
|---|---|---|---|---|
| $E_{vdW}$ | −6114.26 | −6053.65 | −8.05 | −52.56 |
| $E_{ele}$ | −51732.86 | −51483.89 | −44.46 | −204.50 |
| $E_{polar}$ | −8079.50 | −8211.44 | −78.93 | 210.87 |
| $E_{nonpolar}$ | 221.26 | 222.10 | 4.48 | −5.32 |
| $E_{gas}$ | −12542.43 | −12078.12 | −207.26 | −257.05 |
| $E_{solv}$ | −7858.24 | −7989.34 | −74.45 | 205.55 |
| $E_{gas} + E_{sol}$ | −20400.68 | −20067.46 | −281.71 | −51.51 |
| $TS_{total}$ | 71.96 | 71.95 | 37.96 | −37.95 |
| $\Delta G_{bind}^{cal}$ | | | | −13.55 |

The binding free energies ($\Delta G_{bind}^{cal}$) for linagliptin/FAP complex and decomposition to electrostatic interaction ($E_{ele}$), van der Walls interaction ($E_{vdW}$), polar solvation free energies ($E_{polar}$), nonpolar solvation free energies ($E_{nonpolar}$), and entropy ($TS_{total}$). Energy values are presented in kcal/mol. Uncertainties were calculated as the root mean square error for all frames extracted from the trajectories.

**Table S7. Binding free energies ($\Delta G_{bind}^{cal}$) for linagliptin/FAP-I-3 complex system**

| Energy (kcal/mol) | Complex | Receptor | Ligand | Delta |
|---|---|---|---|---|
| $E_{vdW}$ | −6214.40 | −6153.38 | −7.05 | −53.97 |
| $E_{ele}$ | −51768.62 | −51490.71 | −43.20 | −234.72 |
| $E_{polar}$ | −7911.57 | −8075.46 | −78.84 | 242.74 |
| $E_{nonpolar}$ | 208.44 | 209.82 | 4.47 | −5.85 |
| $E_{gas}$ | −12709.88 | −12214.35 | −206.85 | −288.68 |
| $E_{solv}$ | −7703.13 | −7865.65 | −74.38 | 236.89 |
| $E_{gas} + E_{sol}$ | −20413.01 | −20080.00 | −281.22 | −51.79 |
| $TS_{total}$ | 71.15 | 71.14 | 38.11 | −38.10 |
| $\Delta G_{bind}^{cal}$ | | | | −13.69 |

The binding free energies ($\Delta G_{bind}^{cal}$) for linagliptin/FAP complex and decomposition to electrostatic interaction ($E_{ele}$), van der Walls interaction ($E_{vdW}$), polar solvation free energies ($E_{polar}$), nonpolar solvation free energies ($E_{nonpolar}$), and entropy ($TS_{total}$). Energy values are presented in kcal/mol. Uncertainties were calculated as the root mean square error for all frames extracted from the trajectories.

**Table S8. Free energy decomposition for the FAP-linagliptin-I-1 complex on the individual residue basis, where decomposition is performed in terms of the contributions from van der Waals energy, the electrostatic interaction energy, the nonpolar solvation free energy, the polar solvation free energy, the backbone energy, and the side chain energy**

| Residues | $\Delta E_{vdW}$ | | $\Delta E_{ele}$ | | $\Delta E_{polar}$ | | $\Delta E_{nonpolar}$ | | $\Delta E_{subtotal}$ | | $S\Delta E_{subtotal}$ | | $B\Delta E_{subtotal}$ | |
|---|---|---|---|---|---|---|---|---|---|---|---|---|---|---|
| | Avg. | Std. Dev. | Avg. | Std. Dev. | Avg. | Std. Dev. | Avg. | Std. Dev. | Avg. | Std. Dev. | Avg. | Std. Dev. | Avg. | Std. Dev. |
| R123 | −1.53 | 0.26 | 33.01 | 1.63 | −29.11 | 0.93 | −0.25 | 0.04 | 2.12 | 1.05 | 1.94 | 1.03 | 0.18 | 0.04 |
| E203 | −0.69 | 0.69 | −49.05 | 2.22 | 42.41 | 1.71 | −0.07 | 0.02 | −7.40 | 1.38 | −6.48 | 1.51 | −0.92 | 1.10 |
| E204 | −0.80 | 0.75 | −54.07 | 1.98 | 49.39 | 1.93 | −0.11 | 0.02 | −5.58 | 1.39 | −5.05 | 1.37 | −0.53 | 0.14 |
| F350 | −0.26 | 0.37 | −0.57 | 0.23 | 0.70 | 0.17 | −0.04 | 0.07 | −0.18 | 0.34 | −0.19 | 0.34 | 0.01 | 0.01 |
| Y541 | −4.22 | 0.47 | −0.74 | 0.39 | 1.48 | 0.35 | −0.50 | 0.04 | −3.98 | 0.51 | −4.04 | 0.52 | 0.06 | 0.05 |
| W623 | −3.56 | 0.47 | −0.67 | 0.35 | 0.37 | 0.26 | −0.35 | 0.03 | −4.20 | 0.44 | −2.88 | 0.38 | −1.32 | 0.30 |
| S624 | −1.75 | 0.36 | 0.16 | 0.69 | 0.32 | 0.51 | −0.22 | 0.03 | −1.49 | 0.50 | −0.56 | 0.50 | −0.92 | 0.17 |
| Y625 | −1.91 | 0.25 | −1.12 | 0.38 | 0.27 | 0.22 | −0.07 | 0.02 | −2.83 | 0.31 | −1.37 | 0.22 | −1.46 | 0.23 |
| Y656 | −1.30 | 0.45 | 1.45 | 0.98 | −1.08 | 0.57 | −0.14 | 0.03 | −1.07 | 0.77 | −1.11 | 0.78 | 0.04 | 0.05 |
| Y660 | −2.14 | 0.25 | 1.74 | 0.52 | −0.94 | 0.43 | −0.22 | 0.04 | −1.55 | 0.35 | −1.60 | 0.35 | 0.05 | 0.05 |
| N704 | −0.55 | 0.12 | 5.61 | 1.02 | −3.24 | 0.60 | −0.01 | 0.01 | 1.80 | 0.74 | 1.55 | 0.66 | 0.25 | 0.11 |

Energies are in kcal/mol.

**Table S9. Free energy decomposition for the FAP-linagliptin-I-2 complex on the individual residue basis, where decomposition is performed in terms of the contributions from van der Waals energy, the electrostatic interaction energy, the nonpolar solvation free energy, the polar solvation free energy, the backbone energy, and the side chain energy**

| Residues | $\Delta E_{vdW}$ | | $\Delta E_{ele}$ | | $\Delta E_{polar}$ | | $\Delta E_{nonpolar}$ | | $\Delta E_{subtotal}$ | | $S\Delta E_{subtotal}$ | | $B\Delta E_{subtotal}$ | |
|---|---|---|---|---|---|---|---|---|---|---|---|---|---|---|
| | Avg. | Std. Dev. | Avg. | Std. Dev. | Avg. | Std. Dev. | Avg. | Std. Dev. | Avg. | Std. Dev. | Avg. | Std. Dev. | Avg. | Std. Dev. |
| R123 | −1.47 | 0.38 | 32.52 | 2.33 | −28.85 | 1.42 | −0.24 | 0.06 | 1.97 | 0.99 | 1.79 | 0.96 | 0.18 | 0.05 |
| E203 | −0.86 | 0.62 | −47.73 | 4.36 | 42.13 | 3.06 | −0.08 | 0.02 | −6.54 | 1.79 | −5.12 | 1.74 | −1.42 | 1.54 |
| E204 | −0.85 | 0.74 | −54.19 | 2.06 | 49.54 | 1.85 | −0.13 | 0.02 | −5.64 | 1.24 | −5.03 | 1.21 | −0.60 | 0.19 |
| F350 | −0.09 | 0.06 | −0.69 | 0.13 | 0.79 | 0.13 | −0.02 | 0.04 | −0.02 | 0.07 | −0.04 | 0.07 | 0.02 | 0.01 |
| Y541 | −4.28 | 0.47 | −0.68 | 0.35 | 1.43 | 0.32 | −0.51 | 0.03 | −4.04 | 0.61 | −4.11 | 0.62 | 0.07 | 0.04 |
| W623 | −3.86 | 0.49 | −0.42 | 0.44 | 0.43 | 0.28 | −0.37 | 0.03 | −4.22 | 0.49 | −2.89 | 0.41 | −1.33 | 0.31 |
| S624 | −1.87 | 0.40 | −0.32 | 0.66 | 0.50 | 0.49 | −0.24 | 0.02 | −1.93 | 0.46 | −0.93 | 0.48 | −1.01 | 0.17 |
| Y625 | −1.84 | 0.26 | −1.27 | 0.37 | 0.31 | 0.23 | −0.07 | 0.02 | −2.85 | 0.30 | −1.35 | 0.22 | −1.51 | 0.21 |
| Y656 | −1.43 | 0.29 | 1.75 | 0.76 | −1.26 | 0.44 | −0.13 | 0.02 | −1.07 | 0.51 | −1.10 | 0.51 | 0.04 | 0.05 |
| Y660 | −1.80 | 0.27 | 1.74 | 0.58 | −1.00 | 0.47 | −0.21 | 0.03 | −1.27 | 0.33 | −1.30 | 0.33 | 0.02 | 0.04 |
| N704 | −0.45 | 0.12 | 4.78 | 0.94 | −3.06 | 0.59 | −0.01 | 0.01 | 1.26 | 0.53 | 1.06 | 0.47 | 0.20 | 0.09 |

Energies are in kcal/mol.

**Table S10. Free energy decomposition for the FAP-linagliptin-I-3 complex on the individual residue basis, where decomposition is performed in terms of the contributions from van der Waals energy, the electrostatic interaction energy, the nonpolar solvation free energy, the polar solvation free energy, the backbone energy, and the side chain energy**

| Residues | $\Delta E_{vdW}$ | | $\Delta E_{ele}$ | | $\Delta E_{polar}$ | | $\Delta E_{nonpolar}$ | | $\Delta E_{subtotal}$ | | $S\Delta E_{subtotal}$ | | $B\Delta E_{subtotal}$ | |
|---|---|---|---|---|---|---|---|---|---|---|---|---|---|---|
| | Avg. | Std. Dev. | Avg. | Std. Dev. | Avg. | Std. Dev. | Avg. | Std. Dev. | Avg. | Std. Dev. | Avg. | Std. Dev. | Avg. | Std. Dev. |
| R123 | −1.42 | 0.27 | 32.26 | 1.37 | −28.66 | 0.77 | −0.24 | 0.03 | 1.93 | 0.90 | 1.74 | 0.88 | 0.19 | 0.04 |
| E203 | −0.72 | 0.68 | −49.18 | 2.09 | 43.44 | 1.57 | −0.08 | 0.02 | −6.54 | 1.14 | −5.88 | 1.09 | −0.66 | 0.79 |
| E204 | −0.82 | 0.72 | −54.12 | 1.94 | 50.62 | 1.66 | −0.13 | 0.01 | −4.44 | 0.94 | −3.91 | 0.90 | −0.53 | 0.12 |
| F350 | −1.49 | 0.33 | −0.43 | 0.26 | 0.83 | 0.28 | −0.21 | 0.03 | −1.30 | 0.29 | −1.30 | 0.29 | −0.00 | 0.02 |
| Y541 | −4.38 | 0.46 | −0.47 | 0.35 | 1.49 | 0.35 | −0.48 | 0.03 | −3.84 | 0.50 | −3.90 | 0.51 | 0.06 | 0.08 |
| W623 | −3.44 | 0.50 | −0.60 | 0.35 | 0.24 | 0.27 | −0.35 | 0.03 | −4.16 | 0.47 | −2.73 | 0.45 | −1.43 | 0.33 |
| S624 | −1.89 | 0.36 | 0.02 | 0.70 | 0.29 | 0.59 | −0.24 | 0.03 | −1.82 | 0.45 | −1.04 | 0.41 | −0.78 | 0.19 |
| Y625 | −1.93 | 0.24 | −1.30 | 0.42 | 0.48 | 0.21 | −0.09 | 0.02 | −2.84 | 0.31 | −1.37 | 0.19 | −1.48 | 0.25 |
| Y656 | −0.54 | 0.24 | 2.50 | 0.40 | −1.83 | 0.30 | −0.11 | 0.02 | 0.02 | 0.29 | −0.02 | 0.28 | 0.04 | 0.02 |
| Y660 | −1.86 | 0.28 | 1.75 | 0.48 | −1.08 | 0.43 | −0.16 | 0.02 | −1.34 | 0.27 | −1.38 | 0.27 | 0.04 | 0.04 |
| N704 | −0.51 | 0.14 | 5.67 | 1.49 | −3.79 | 1.04 | −0.02 | 0.01 | 1.35 | 0.66 | 1.17 | 0.63 | 0.17 | 0.06 |

Energies are in kcal/mol.

**Table S11. Binding free energies ($\Delta G_{bind}^{cal}$) for linagliptin/FAP-II-1 complex system**

| Energy (kcal/mol) | Complex | Receptor | Ligand | Delta |
|---|---|---|---|---|
| $E_{vdW}$ | −6155.20 | −6096.34 | −7.99 | −50.88 |
| $E_{ele}$ | −51686.60 | −51426.41 | −43.57 | −216.63 |
| $E_{polar}$ | −8010.76 | −8153.53 | −78.70 | 221.47 |
| $E_{nonpolar}$ | 218.74 | 219.52 | 4.51 | −5.29 |
| $E_{gas}$ | −12583.96 | −12109.33 | −207.12 | −267.50 |
| $E_{solv}$ | −7792.02 | −7934.01 | −74.19 | 216.18 |
| $E_{gas} + E_{sol}$ | −20375.98 | −20043.34 | −281.31 | −51.33 |
| $TS_{total}$ | 71.59 | 71.57 | 39.35 | −39.33 |
| $\Delta G_{bind}^{cal}$ | | | | −12.00 |

The binding free energies ($\Delta G_{bind}^{cal}$) for inhibitor/FAP complex and decomposition to electrostatic interaction ($E_{ele}$), van der Walls interaction ($E_{vdW}$), polar solvation free energies ($E_{polar}$), nonpolar solvation free energies ($E_{nonpolar}$), and entropy ($TS_{total}$). Energy values are presented in kcal/mol. Uncertainties were calculated as the root mean square error for all frames extracted from the trajectories.

**Table S12. Binding free energies ($\Delta G_{bind}^{cal}$) for linagliptin/FAP-II-2 complex system**

| Energy (kcal/mol) | Complex | Receptor | Ligand | Delta |
|---|---|---|---|---|
| $E_{vdW}$ | −6151.47 | −6089.46 | −8.61 | −53.41 |
| $E_{ele}$ | −51679.30 | −51425.57 | −45.18 | −208.56 |
| $E_{polar}$ | −8061.29 | −8199.63 | −78.35 | 216.69 |
| $E_{nonpolar}$ | 217.80 | 218.75 | 4.49 | −5.44 |
| $E_{gas}$ | −12529.73 | −12058.79 | −208.98 | −261.96 |
| $E_{solv}$ | −7843.49 | −7980.88 | −73.86 | 211.24 |
| $E_{gas} + E_{sol}$ | −20373.22 | −20039.67 | −282.84 | −50.72 |
| $TS_{total}$ | 71.66 | 71.65 | 37.49 | −37.47 |
| $\Delta G_{bind}^{cal}$ | | | | −13.24 |

The binding free energies ($\Delta G_{bind}^{cal}$) for inhibitor/FAP complex and decomposition to electrostatic interaction ($E_{ele}$), van der Walls interaction ($E_{vdW}$), polar solvation free energies ($E_{polar}$), nonpolar solvation free energies ($E_{nonpolar}$), and entropy ($TS_{total}$). Energy values are presented in kcal/mol. Uncertainties were calculated as the root mean square error for all frames extracted from the trajectories.

**Table S13. Binding free energies ($\Delta G_{bind}^{cal}$) for linagliptin/FAP-II-3 complex system**

| Energy (kcal/mol) | Complex | Receptor | Ligand | Delta |
|---|---|---|---|---|
| $E_{vdW}$ | −6125.10 | −6064.85 | −8.00 | −52.25 |
| $E_{ele}$ | −51861.47 | −51591.99 | −43.26 | −226.22 |
| $E_{polar}$ | −7888.75 | −8042.42 | −78.93 | 232.61 |
| $E_{nonpolar}$ | 218.06 | 218.93 | 4.49 | −5.36 |
| $E_{gas}$ | −12684.23 | −12198.93 | −206.84 | −278.46 |
| $E_{solv}$ | −7670.69 | −7823.49 | −74.45 | 227.24 |
| $E_{gas} + E_{sol}$ | −20354.92 | −20022.42 | −281.29 | −51.22 |
| $TS_{total}$ | 71.90 | 71.89 | 38.21 | −38.20 |
| $\Delta G_{bind}^{cal}$ | | | | −13.02 |

The binding free energies ($\Delta G_{bind}^{cal}$) for inhibitor/FAP complex and decomposition to electrostatic interaction ($E_{ele}$), van der Walls interaction ($E_{vdW}$), polar solvation free energies ($E_{polar}$), nonpolar solvation free energies ($E_{nonpolar}$), and entropy ($TS_{total}$). Energy values are presented in kcal/mol. Uncertainties were calculated as the root mean square error for all frames extracted from the trajectories.

**Table S16. System information for linagliptin binding with FAP**

| Number | Linagliptin/FAP-I | Linagliptin/FAP-II |
|---|---|---|
| $Na^+$ (Atom) | 102 | 102 |
| $Cl^-$ (Atom) | 95 | 95 |
| Water (Residue) | 36487 | 36487 |
| Solute (Residue) | 722 | 722 |
